# Facile, Regio- and Diastereoselective Synthesis of Spiro-Pyrrolidine and Pyrrolizine Derivatives and Evaluation of Their Antiproliferative Activities

**DOI:** 10.3390/molecules190710033

**Published:** 2014-07-10

**Authors:** Abdulrahman I. Almansour, Raju Suresh Kumar, Farzana Beevi, Amir Nasrolahi Shirazi, Hasnah Osman, Rusli Ismail, Tan Soo Choon, Brian Sullivan, Kellen McCaffrey, Alaa Nahhas, Keykavous Parang, Mohamed Ashraf Ali

**Affiliations:** 1 Department of Chemistry, College of Science, King Saud University, P.O.Box 2455, Riyadh 11451, Saudi Arabia; 2 New Drug Discovery Research, Department of Medicinal Chemistry, Sunrise University, Alwar, Rajasthan-301030, India; 3 Department of Biomedical and Pharmaceutical Sciences, College of Pharmacy, University of Rhode Island, Kingston, RI 02881, USA; 4 School of Chemical Sciences, Universiti Sains Malaysia, Minden 11800, Penang, Malaysia; 5 Institute for Research in Molecular Medicine, Universiti Sains Malaysia, Minden 11800, Penang, Malaysia; 6 School of Pharmacy, Chapman University, Irvine, CA 92618, USA; 7 Centre of Excellence for Research in AIDS, University Malaya, Kuala Lumpur 50603, Malaysia

**Keywords:** antiproliferative activity, diastereoselective synthesis, pyrrolizine, regio-selective synthesis, spiro-pyrolidine

## Abstract

A number of novel spiro-pyrrolidines/pyrrolizines derivatives were synthesized through [3+2]-cycloaddition of azomethine ylides with 3,5-bis[(*E*)-arylmethylidene]tetrahydro-4(1*H*)-pyridinones **2a**–**n**. Azomethine ylides were generated *in situ* from the reaction of 1*H*-indole-2,3-dione (isatin, **3**) with *N*-methylglycine (sarcosine), phenylglycine, or proline. All compounds (50 μM) were evaluated for their antiproliferative activity against human breast carcinoma (MDA-MB-231), leukemia lymphoblastic (CCRF-CEM), and ovarian carcinoma (SK-OV-3) cells. *N*-α-Phenyl substituted spiro-pyrrolidine derivatives (**5a**–**n**) showed higher antiproliferative activity in MDA-MB-231 than other cancer cell lines. Among spiro-pyrrolizines **6a**–**n**, a number of derivatives including **6a**–**c** and **6i**–**m** showed a comparable activity with doxorubicin in all three cell lines. Among all compounds in three classes, **6a**, **6b**, and **6m**, were found to be the most potent derivatives showing 64%, 87%, and 74% antiproliferative activity in MDA-MB-231, SK-OV-3, and CCRF-CEM cells, respectively. Compound **6b** showed an IC_50_ value of 3.6 μM in CCRF-CEM cells. These data suggest the potential antiproliferative activity of spiro-pyrrolidines/pyrrolizines.

## 1. Introduction

Multicomponent reactions (MCRs) [1,2,3,4,5] constitute an efficient and powerful tool for the synthesis of novel organic compounds. MCRs take advantage of several distinct properties including low cost, accelerated reaction time, and eco-friendly reaction conditions [6,7,8,9], and provide an expeditious and elegant access to libraries of complex structures and diversified compounds. Thus, MCRs are widely used in combinatorial chemistry and complicated synthetic procedures [10,11,12,13,14,15].

Functionalized pyrrolidines and pyrrolizines are the central skeleton of numerous alkaloids and constitute classes of compounds with significant biological properties, such as anticancer activity [16,17,18]. Heterocycles containing piperidine sub-structures display important biological activities, such as anticancer activity as well as being useful as synthons in the construction of alkaloid natural products [19,20,21]. Dimmock *et al.* reported the synthesis of 3,5-bis[(*E*)-arylmethyl idene] tetrahydro-4(1*H*)-pyridinones and their corresponding substituted analogues as potential anticancer agents with modest to high activity [22].

Several spiro-compounds have shown very promising biologically activity with potential applications as anticancer [21,22,23,24,25], antibacterial [26,27], anticonvulsant [28,29,30], anti-tuberculosis [31], and anti-Alzheimer’s disease agents [31]. Spiro compounds have also been recently used as antioxidant agents [32,33].

[3+2]-Cycloaddition of azomethine ylides with olefinic dipolarophiles has been reported as one of the main methods for generating highly functionalized heterocyclic scaffolds as diversified chemical libraries [33,34,35,36,37,38,39]. Inspired by the previously reported biological potency of spiro compounds and as a part of our ongoing research in the construction of novel hybrid heterocycles, herein we report the synthesis of three different classes of spiro-pyrrolidines and spiro-pyrrolizines derivatives by the [3+2]-cycloaddition of azomethine ylides with 3,5-bis[(*E*)-arylmethylidene]tetrahydro-4(1*H*)-pyridinones and evaluation of their anticancer activities.

## 2. Results and Discussion

### 2.1. Chemistry

**Scheme 1:**
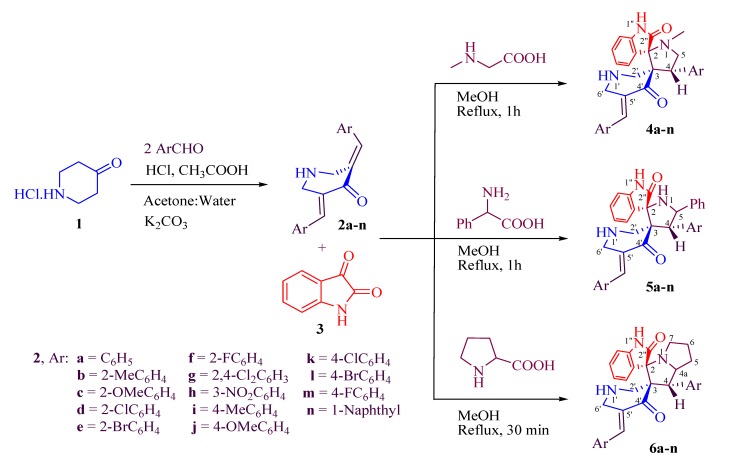
Synthesis of piperidone grafted spiroheterocycles.

The synthesis of the prerequisite 3,5-bis[(*E*)-arylmethyl idene]tetrahydro-4(1*H*)-pyridinones **2** was carried out according to the previously reported procedure from the reaction of an appropriate aryl aldehyde (2 mmol) with 4-piperidone hydrochloride monohydrate (**1**, 1 mmol) in acetic acid [25]. Azomethine ylides were generated *in situ* from the reaction of 1*H*-indole-2,3-dione (isatin, **3**) with (i) *N*-methylglycine (sarcosine); (ii) phenylglycine; and (iii) proline. The [3+2]-cycloaddition of azomethine ylides with the exocyclic dipolarophiles (2) afforded the novel *N*-methyl substituted spiro-pyrrolidines **4a**–**n**, *N*-α-phenyl substituted spiro-pyrrolidine derivatives **5a**–**n**, and spiro-pyrrolizines **6a**–**n**, respectively, in reasonable yields. All the reactions were performed by heating the mixture of **2a**–**n**, 1*H*-indole-2,3-dione (3) and *N*-methylglycine/phenylglycine/proline in a molar ratio 1:1.1:1.1 under reflux in methanol. All compounds were isolated as in a form of racemic mixtures in 82%–94% (Scheme 1).

For instance, the synthesis of spiro-pyrrolidine derivatives **4** and **5** by using *N*-methylglycine or phenylglycine, respectively, was completed within 1 h. In comparison, the reaction with proline took 30 min to afford the corresponding spiro-pyrrolizine derivatives **6**. All substrates carrying aromatic rings with electron-withdrawing and electron-donating substituents afforded the product in high to excellent yields within approximately similar time ranges (Table S1, Supporting Information). The cycloaddition reaction worked well regardless of the position and electronic or steric properties of the substituents at the aromatic rings of **2**. The reaction of 1*H*-indole-2,3-dione with *N*-methylglycine, phenylglycine, or proline afforded the corresponding azomethine ylide, which was added to one of the exocyclic C=C bonds of the bisdipolarophile **2** to form the corresponding cycloadducts **4**, **5**, or **6**, respectively. The structures of cycloadducts **4**–**6** were characterized using elemental analysis, FT-IR, ^1^H, ^13^C and 2D-NMR spectroscopic analysis.

**Scheme 2:**
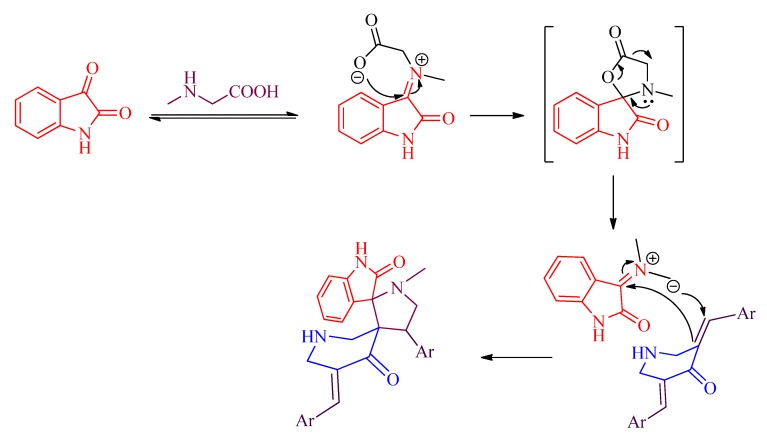
Mechanism for the formation of spiro-pyrrolidines.

A proposed mechanism for the formation of spiro-pyrrolidines is shown in Scheme 2. It is noteworthy to mention that all reactions proceeded chemoselectively since the dipole addition was occurred only to the available C=C bond rather than C=O functional group of **2**. All reactions were also found to be regioselective, which can be viewed as the result of a preferential attack of the nucleophilic carbon of the azomethine ylide to the end of the enone fragment of the dipolarophile **2** to give **4**, **5**, or **6**. Another significant advantage of this method is that all the above reactions proceeded *via* complete stereoselectivity leading to the production of a single stereoisomer despite the presence of many stereocenters in the cycloadducts.

### 2.2. Biological Evaluation

Starting building blocks **2a**–**n** and three classes of synthesized compounds **3a**–**n**, **4a**–**n**, and **6a**–**n** (50 μM) were evaluated for their effect on proliferation of human ovarian adenocarcinoma (SK-OV-3), breast adenocarcinoma (MDA-MB-231), and lymphoblastic leukemia (CCRF-CEM). Doxorubicin (Dox) and DMSO were used as positive and negative controls, respectively.

**Figure 1:**
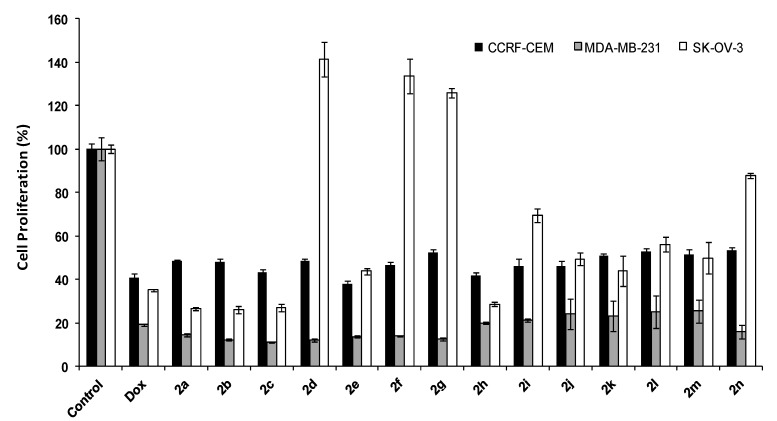
Antiproliferative activity of 3,5-bis[(*E*)-arylmethylidene] tetrahydro -4(1*H*)-pyridinones **2a**–**n**.

The results for cell proliferation at 50 μM after 72 h for 3,5-bis[(*E*)-arylmethylidene]tetrahydro-4(1*H*)-pyridinones **2a**–**n** are shown in Figure 1. Compounds **2a**–**g** inhibited the cell proliferation of MDA-MB-231 cells by 85%–88%. The presence of a wide range of substituents on two side aromatic rings was tested. The majority of derivatives containing electron donating groups including methyl and methoxy, showed higher antiproliferative actvity than compounds with electron withdrawing groups e.g., nitro in MDA-MB-231 cells after 72 h incubation. Compounds with substitutions on *para* positions were exhibited slightly lower inhibition activity. This trend was also observed in SK-OV-3 and CCRF-CEM cells. All the compounds in this class showed higher antiproliferative activity in MDA-MB-231 than CCRF-CEM and SK-OV-3 cells. Thus, MDA-MB-231 cells were found to be the most sensitive cell line to these compounds among the three that have tested.

**Figure 2:**
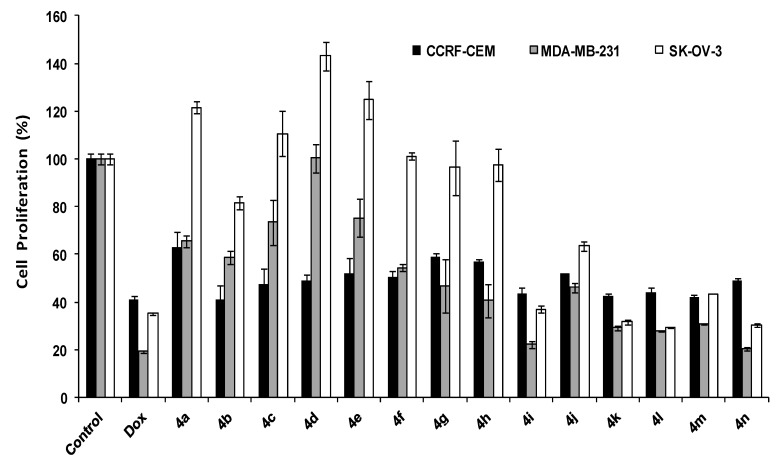
Antiproliferative activity of *N*-methylspiro-pyrrolidine derivatives **4a**–**n**.

*N*-Methyl spiro-pyrrolidine derivatives **4a**–**n** antiproliferative activities are shown in Figure 2. Among all compounds in this class, **4k**, **4n**, and **4l** showed the highest antiproliferative activity in CCRF-CEM, MDA-MB-231, and SK-OV-3 cells by 58%, 80%, and 70% inhibition, respectively. Most of the compounds showed higher antiproliferative activity in MDA-MB-231 cells than other cancer cell lines. However, some compounds including **4i**, **4k**, **4l**, **4m**, and **4n** exhibited a consistent potency in all cell lines. The position of substituents on aromatic rings was found to be critical in antiproliferative activity against SK-OV-3 and MDA-MB-231 cells. Compounds **4i**–**m** with the *para* substituents on the aromatic rings showed significantly higher activity when compared with compounds **4a**–**h** with substituents on the *ortho* and *meta* positions.

**Figure 3:**
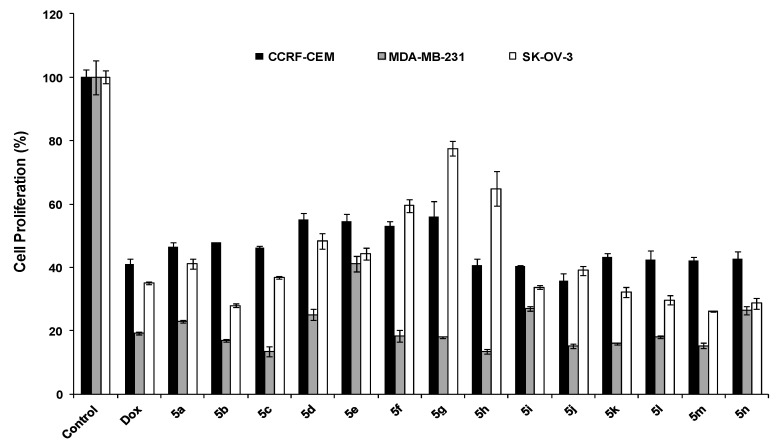
Antiproliferative activity of *N*-α-phenyl substituted spiro-pyrrolidines derivatives **5a**–**n**.

*N*-α-Phenyl substituted spiro-pyrrolidines derivatives **5a**–**n** were evaluated for their antiproliferative potency against SK-OV-3, MDA-MB-231, and CCRF-CEM cells. All compounds showed higher antiproliferative potency in MDA-MB-231 than other cancer cell lines (Figure 3).

**Figure 4:**
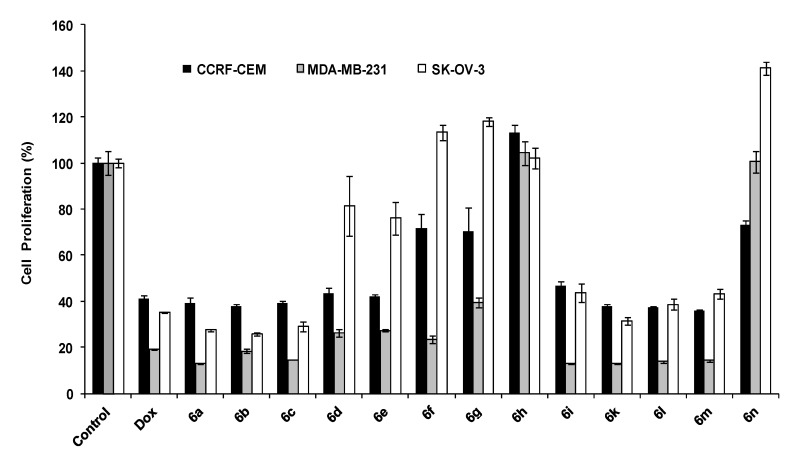
Antiproliferative activity of **6a**–**n**.

The majority of compounds showed modest to modest antiproliferative activity in MDA-MB-231 cells by inhibiting the proliferation in a range of 59% to 87%. The potency of compound **5g** was found to be cell-specific since this compound inhibited the proliferation of MDA-MB-231, CCRF-CEM, and SK-OV-3 by 82%, 44%, and 22%, respectively. In general, the *N*-α-phenyl substituted spiro-pyrrolidines derivatives **5a**–**n** showed higher antiproliferative activity when compared with the corresponding *N*-methyl spiro-pyrrolidine derivatives **4a**–**n**. For instance, 2-chlorosubstituted **4d** did not inhibit the proliferation of SK-OV-3 and MDA-MB-231 cells. However, after the replacement of hydrogen with a phenyl ring at position 5 in the corresponding 2-chlorosubstituted compound **5d**, the antiproliferative potency elevated significantly by 52%, 75% in SK-OV-3 and MDA-MB-231, respectively, suggesting that the presence of phenyl group contributes to the improvement of the antiproliferative potency of the compound. Finally, a class of spiro-pyrrolizines derivatives was examined for their antiproliferative activity against SK-OV-3, MDA-MB-231, and CCRF-CEM cells (Figure 4).

A number of derivatives including **6a**–**c** and **6i**–**m** compounds showed a comparable activity with Dox in all three cell lines. Among all derivatives, compound **6m** inhibited the proliferation of CCRF-CEM cells by 64%. Similarly, compound **6a** inhibited MDA-MB-231 proliferation by 87%. However, compound **6b** exhibited higher activity in SK-OV-3 cells by inhibiting their growth up to 74%, respectively. The antiproliferative activity of compounds **6f**, **6h** and **6n** were decreased significantly when compared to their corresponding compounds **5f**, **5h** and **5n**. The other spiro-pyrrolizines derivatives in this class showed comparable or higher antiproliferative activity when compared with spiro-pyrrolidines derivatives, suggesting that the less rigidity of the chemical structure contributes possibly to their antiproliferative activity. Compounds **6a** and **6b** exhibited IC_50 _values of 25.2 and 3.6 μM in CCRF-CEM cells and 38.9 and 35.8 μM in SK-OV-3 cells after 72 h incubation.

## 3. Experimental

### 3.1. General Methods

The melting points were measured using open capillary tubes and are uncorrected. ^1^H, ^13^C andtwo-dimensional NMR spectra were recorded on a Bruker 500, 400 and 300 MHz instruments in CDCl_3, _MeOD, and DMSO using TMS as internal standard. Chemical shifts are given in parts per million (-scale) and the coupling constants are given in Hertz. IR spectra were recorded on a JASCO FT IR instrument (KBr pellets). Elemental analyses were performed on a Perkin Elmer 2400 Series II Elemental CHNS analyzer. Column chromatography was performed on silica gel (230–400 mesh) using petroleum ether-ethyl acetate as eluents.

### 3.2. General Procedure for the Synthesis of 3,5-bis[(E)-Arylmethylidene]tetrahydro-4(1H)-pyridinones **2**

Following the literature reported procedure by Dimmock *et al*., an appropriate aryl aldehyde (2 mmol) was added to a suspension of 4-piperidone hydrochloride monohydrate (1 mmol) in acetic acid (40 mL). Dry hydrogen chloride was passed through this mixture for 30 min during which time a clear solution was obtained and the stirring continued for 24 h. The precipitate obtained was collected and added to a mixture of saturated aqueous potassium carbonate solution and acetone. The resultant mixture was stirred for 30 min, the free base collected was washed with water and dried and crystalized with ethyl acetate to afford **2** in good yield.

### 3.3. General Procedure for the Synthesis of 1-Methyl-4-(aryl)pyrrolo-(spiro[2.3″]oxindole)-spiro[3.3′]-5′(arylmethylidene)-piperidin-4′-ones **4**

A mixture of 3,5-bis[(*E*)-arylmethylidene]tetrahydro-4(1*H*)-pyridinone (1 mmol), isatin (1.1 mmol), and sarcosine (1.1 mmol) were dissolved in methanol (5 mL) and heated under reflux for 1 h. After completion of the reaction as evident from TLC, the mixture was poured into water (50 mL). The precipitated solid was filtered and washed with water to obtain the corresponding product **4** in good yield.

### 3.4. Spectral Data

*1-Methyl-4-(phenyl)pyrrolo-(spiro[2.3″]oxindole)-spiro[3.3′]-5′-(phenylmethylidene)-piperidin-4′-one* (**4a**). Obtained as a pale yellow solid, (0.150 g, 92%); mp = 169–171°C; IR (KBr): 1604, 1620, 1703, 3407 cm^−1^; ^1^H-NMR (300 MHz, CDCl_3_): δ_H_ 2.05 (d, 1H, *J =* 13.2 Hz, 2ꞌ-CH_2_), 2.14 (s, 1H, N-CH_3_), 3.35 (dd, 1H, *J* = 7.2, 7.2 Hz, 5-CH_2_), 3.50–3.58 (m, 2H, 6ꞌ-CH_2_), 3.64 (d, 1H, *J* = 13.2 Hz, 2ꞌ-CH_2_), 3.94 (dd, 1H, *J* = 9.0, 8.7 Hz, 5-CH_2_), 4.85 (dd, 1H, *J* = 7.2, 7.2 Hz, 4-CH), 6.66–7.43 (m, 15H, Ar-H), 8.41 (s, 1H, 1ꞌꞌ-NH). ^13^C-NMR (75 MHz, CDCl_3_): δ_C_ 35.07, 46.43, 48.66, 50.38, 57.35, 66.75, 76.29, 109.46, 122.55, 127.22, 127.99, 128.29, 128.59, 128.66, 128.97, 129.26, 130.01, 130.24, 135.16, 135.63, 137.75, 138.97, 142.15, 178.69, 199.98. Anal. calcd for C_29_H_27_N_3_O_2_: C, 77.48; H, 6.05; N, 9.35; found: C, 77.34; H, 6.23; N, 9.28.

*1-Methyl-4-(2-methylphenyl)pyrrolo-(spiro[2.3″]oxindole)-spiro[3.3′]-5′-(2-methylphenylmethylidene) piperidin-4′-one* (**4b**). Obtained as a pale yellow solid, (0.140 g, 90%); mp = 163–164 °C; IR (KBr): 1602, 1615, 1711, 3405 cm^−^^1^; ^1^H-NMR (400 MHz, CDCl_3_): δ_H_ 2.06–2.09 (m, 1H, 2'-CH_2_), 2.15 (s, 3H, N-CH_3_), 2.17 (s, 3H, CH_3_), 2.32 (s, 3H, CH_3_), 3.25–3.56 (m, 4H, 5-CH_2_, 6'-CH_2_ and 2'-CH_2_), 4.00 (t, 1H, *J* = 9.2 Hz, 5-CH_2_), 4.98 (t, 1H, *J* = 8.0 Hz, 4-CH), 6.72–7.57 (m, 11H, Ar-H), 7.70 (d, 1H, *J* = 6.8 Hz, Ar-H), 7.83 (d, 1H, *J* = 8.0 Hz, Ar-H), 8.23 (s, 1H, 1"-NH). ^13^C-NMR (100 MHz, CDCl_3_): δ_C_ 20.44, 21.55, 35.06, 42.46, 48.66, 51.44, 58.81, 64.72, 76.04, 109.60, 122.98, 125.69, 126.21, 126.99, 128.38, 128.85, 128.99, 129.18, 129.74, 129.83, 130.47, 130.56, 130.61, 131.15, 134.12, 134.54, 137.53, 138.09, 142.30, 178.21, 199.75. Anal. calcd for C_31_H_31_N_3_O_2_: C, 77.96; H, 6.54; N, 8.80; found: C, 77.81; H, 6.65; N, 8.69.

*1-Methyl-4-(2-methoxyphenyl)pyrrolo-(spiro[2.3″]oxindole)-spiro[3.3′]-5′-(2-methoxyphenylmethylid ene)piperidin-4′-one* (**4c**). Obtained as a white solid, (0.130 g, 86%); mp = 159–160 °C; IR (KBr): 1599, 1617, 1705, 3410 cm^−^^1^; ^1^H-NMR (300 MHz, CDCl_3_): δ_H_ 2.15 (s, 3H, N-CH_3_), 2.30 (d, 1H, *J* = 13.8 Hz, 2'-CH_2_), 3.24–3.71 (m, 4H, 5-CH_2_, 6'-CH_2_ and 2'-CH_2_), 3.73 (s, 3H, OCH_3_), 3.81 (s, 3H, OCH_3_), 4.10 (dd, 1H, *J* = 9.3, 9.0 Hz, 5-CH_2_), 4.93 (dd, 1H, *J* = 8.1, 7.5 Hz, 4-CH), 6.68–7.65 (m, 13H, Ar-H), 8.37 (s, 1H, 1"-NH). ^13^C-NMR (75 MHz, CDCl_3_): δ_C_ 35.28, 40.39, 48.42, 55.07, 55.34, 55.71, 56.28, 64.03, 76.44, 109.66, 110.25, 110.94, 120.18, 120.89,122.50, 122.89, 124.90, 126.47, 127.96, 128.72, 128.98, 129.18, 130.48, 130.63, 133.59, 134.32, 142.28, 158.34, 158.48, 178.51, 199.07. Anal. calcd for C_31_H_31_N_3_O_4_: C, 73.06; H, 6.13; N, 8.25; found: C, 73.28; H, 6.29; N, 8.32.

*1-Methyl-4-(2-chlorophenyl)pyrrolo-(spiro[2.3″]oxindole)-spiro[3.3′]-5′-(2-chlorophenylmethylidene)piperidin-4′-one* (**4d**). Obtained as a white solid, (0.137 g, 91%); mp = 168–169 °C; IR (KBr): 1598, 1615, 1706, 3409 cm^−^^1^; ^1^H-NMR (400 MHz, CDCl_3_): δ_H_ 2.03 (d, 1H, *J* = 14.2 Hz, 2'-CH_2_), 2.13 (s, 3H, N-CH_3_), 3.25–3.57 (m, 4H, 5-CH_2_, 6'-CH_2_ and 2'-CH_2_), 3.99 (t, 1H, *J* = 8.8 Hz, 5-CH_2_), 5.14 (t, 1H, *J* = 8.8 Hz, 4-CH), 6.72 (d, 1H, *J* = 7.6 Hz, Ar-H), 6.87–7.31 (m, 8H, Ar-H), 7.34 (d, 2H, *J* = 8.0 Hz, Ar-H), 7.64 (s, 1H, C=CH), 8.00 (d, 1H, *J =* 8.0 Hz, Ar-H), 8.31 (s, 1H, 1"-NH). ^13^C-NMR (100 MHz, CDCl_3_): δ_C_ 35.12, 43.14, 48.52, 50.83, 57.97, 64.01, 77.67, 109.62, 123.54, 126.56, 126.83, 127.12, 128.37, 128.58, 129.32, 129.59, 129.64, 130.10, 130.48, 131.29, 134.06, 135.05, 135.30, 135.61, 136.27, 137.30, 141.98, 178.20, 198.60. Anal. calcd for C_29_H_25_Cl_2_N_3_O_2_: C, 67.19; H, 4.86; N, 8.11; found: C, 67.30; H, 4.70; N, 8.04.

*1-Methyl-4-(2-bromophenyl)pyrrolo-(spiro[2.3ꞌꞌ]oxindole)-spiro[3.3′]-5′-(2-bromophenylmethylidene)piperidin-4′-one* (**4e**). Obtained as a white solid, (0.126 g, 90%); mp = 143–144 °C; IR (KBr): 1605, 1614, 1702, 3412 cm^−^^1^; ^1^H-NMR (400 MHz, CDCl_3_): δ_H_ 1.99 (d, 1H, *J =* 14.8 Hz, 2'-CH_2_), 2.13 (s, 3H, N-CH_3_), 3.24–3.57 (m, 4H, 5-CH_2_, 6'-CH_2_ and 2'-CH_2_), 3.96 (t, 1H, *J =* 8.8 Hz, 5-CH_2_), 5.08 (t, 1H, *J =* 8.4 Hz, 4-CH), 6.74 (d, 1H, *J =* 7.6 Hz, Ar-H), 6.89 (d, 1H, *J =* 7.2 Hz, Ar-H), 6.98–7.61 (m, 8H, Ar-H), 7.65 (s, 1H, C=CH), 7.94 (t, 1H, *J =* 8.8 Hz, Ar-H), 8.05 (d, 1H, *J =* 7.6 Hz, Ar-H), 8.42 (s, 1H, 1"-NH). ^13^C-NMR (100 MHz, CDCl_3_): δ_C_ 35.10, 46.09, 48.27, 50.78, 58.47, 63.85, 76.14, 109.68, 123.64, 125.46, 126.81, 127.19, 127.64, 127.70, 128.59, 128.69, 129.33, 130.24, 130.46, 131.73, 132.95, 133.01, 133.35, 135.85, 137.26, 139.08, 142.05, 178.22, 198.58. Anal. calcd for C_29_H_25_Br_2_N_3_O_2_: C, 57.35; H, 4.15; N, 6.92; found: C, 57.52; H, 4.01; N, 6.84.

*1-Methyl-4-(2-fluorophenyl)pyrrolo-(spiro[2.3″]oxindole)-spiro[3.3′]-5′-(2-fluorophenylmethylidene)piperidin-4′-one* (**4f**). Obtained as a white solid, (0.143 g, 92%); mp = 165–166 °C; IR (KBr): 1603, 1617, 1705, 3408 cm^−^^1^; ^1^H-NMR (400 MHz, CDCl_3_): δ_H _ 2.14 (s, 3H, N-CH_3_), 2.30 (d, 1H, *J =* 13.6 Hz, 2'-CH_2_), 3.27–3.55 (m, 4H, 5-CH_2_, 6'-CH_2_ and 2'-CH_2_), 3.98 (t, 1H, *J =* 9.6 Hz, 5-CH_2_), 5.08 (t, 1H, *J =* 9.6 Hz, 4-CH), 6.68 (d, 1H, *J =* 7.6 Hz, Ar-H), 6.83–7.32 (m, 11H, Ar-H), 7.76 (t, 1H, *J =* 7.2 Hz, Ar-H), 8.01 (s, 1H, 1"-NH). ^13^C-NMR (100 MHz, CDCl_3_): δ_C_ 35.07, 38.61, 48.80, 50.68, 56.97, 65.03, 76.89, 109.42, 115.45 (^2^*J*_CF_ = 22.6 Hz), 116.00 (^2^*J*_CF_ = 21.8 Hz), 122.99, 123.92, 123.96, 126.49, 127.15, 128.25, 128.59, 128.73, 129.15, 129.30, 130.68, 130.79, 130.87, 136.23, 141.90, 160.68, 162.23, 178.20, 198.37. Anal. calcd for C_29_H_25_F_2_N_3_O_2_: C, 71.74; H, 5.19; N, 8.65; found: C, 71.95; H, 5.08; N, 8.78.

*1-Methyl-4-(2,4-dichlorophenyl)pyrrolo-(spiro[2.3″]oxindole)-spiro[3.3′]-5′-(2,4-dichlorophenylmeth ylidene)piperidin-4′-one* (**4g**). Obtained as a white solid, (0.127 g, 90%); mp = 171–172 °C; IR (KBr): 1600, 1619, 1708, 3406 cm^−^^1^; ^1^H-NMR (300 MHz, CDCl_3_): δ_H_ 2.11 (s, 3H, N-CH_3_), 2.22–2.25 (m, 1H, 2'-CH_2_), 3.27–3.56 (m, 4H, 6'-CH_2_,2'-CH_2 _and 5-CH_2_), 3.91 (t, 1H, *J =* 9.0 Hz, 5-CH_2_), 5.07 (t, 1H, *J =* 8.7 Hz, 4-CH), 6.69–7.44 (m, 8H, Ar-H), 7.54 (s, 1H, Ar-H), 7.86 (d, 1H, *J =* 8.4 Hz, Ar-H), 7.95 (d, 1H, *J =* 8.7 Hz, Ar-H), 8.77 (s, 1H, 1"-NH). ^13^C-NMR (75 MHz, CDCl_3_): δ_C_ 35.10, 42.65, 48.11, 49.95, 57.10, 63.92, 76.54, 109.20, 123.47, 125.93, 126.55, 127.03, 128.36, 129.34, 129.48, 130.06, 130.87, 132.36, 132.54, 133.46, 133.93, 134.41, 135.45, 135.80, 136.04, 136.78, 142.15, 178.33, 198.39. Anal. calcd for C_29_H_23_Cl_4_N_3_O_2_: C, 59.30; H, 3.95; N, 7.15; found: C, 59.45; H, 3.77; N, 7.01.

*1-Methyl-4-(3-nitrophenyl)pyrrolo-(spiro[2.3″]oxindole)-spiro[3.3′]-5′-(3-nitrophenylmethylidene)piperidin-4′-one* (**4h**). Obtained as a pale yellow solid, (0.131 g, 89%); mp = 185–186 °C; IR (KBr): 1599, 1617, 1705, 3408 cm^−1^; ^1^H-NMR (400 MHz, MeOH): δ_H_ 2.09–2.13 (m, 1H, 2'-CH_2_), 2.17 (s, 3H, N-CH_3_), 3.44 (t, 1H, *J =* 7.6 Hz, 5-CH_2_), 3.51–3.60 (m, 2H, 6'-CH_2_), 3.64 (d, 1H, *J =* 12.8 Hz, 2'-CH_2_) 3.94 (t, 1H, *J =* 9.6 Hz, 5-CH_2_), 4.90 (dd, 1H, *J =* 7.6, 7.2 Hz, 4-CH), 6.74 (d, 1H, *J =* 7.6 Hz, Ar-H), 6.92–8.12 (m, 11H, Ar-H), 8.34 (s, 1H, 1"-NH). ^13^C-NMR (100 MHz, MeOH): δ_C_ 34.92, 45.92, 48.40, 50.39, 57.54, 66.71, 76.10, 109.75, 122.50, 122.62, 123.56, 124.40, 124.74, 127.41, 128.20, 129.59, 129.68, 129.73, 134.98, 135.67, 136.39, 137.03, 137.12, 141.23, 142.22, 148.50, 148.75, 178.18, 199.03. Anal. calcd for C_29_H_25_N_5_O_6_: C, 64.56; H, 4.67; N, 12.98; found: C, 64.68; H, 4.59; N, 12.85.

*1-Methyl-4-(4-methylphenyl)pyrrolo-(spiro[2.3″]oxindole)-spiro[3.3′]-5′-(4-methylphenylmethylidene)piperidin-4′-one* (**4i**). Obtained as a white solid, (0.146 g, 93%); mp = 180–181 °C; IR (KBr): 1607, 1621, 1710, 3412 cm^−^^1^; ^1^H-NMR (400 MHz, CDCl_3_): δ_H _2.14 (s, 3H, N-CH_3_), 2.20 (d, 1H, *J =* 13.6 Hz,2'-CH_2_), 2.30 (s, 3H, CH_3_), 2.31 (s, 3H, CH_3_), 3.15–3.63 (m, 4H, 5-CH_2_, 6'-CH_2_ and 2'-CH_2_), 3.91 (t, 1H, *J =* 9.6 Hz, 5-CH_2_), 4.80 (t, 1H, *J =* 9.6 Hz, 4-CH), 6.63–7.32 (m, 13H, Ar-H), 8.08 (s, 1H, 1"-NH). ^13^C-NMR (100 MHz, CDCl_3_): δ_C_ 21.43, 21.69, 35.04, 46.18, 48.73, 50.29, 57.51, 66.51, 76.40, 109.33, 122.55, 128.33, 129.19, 129.54, 129.71, 129.89, 130.16, 130.41, 132.84, 134.38, 135.83, 136.77, 137.80, 139.27, 142.02, 178.64, 200.02. Anal. calcd for C_31_H_31_N_3_O_2_: C, 77.96; H, 6.54; N, 8.80; found: C, 78.10; H, 6.46; N, 8.73.

*1-Methyl-4-(4-methoxyphenyl)pyrrolo-(spiro[2.3″]oxindole)-spiro[3.3′]-5′-(4-methoxyphenylmethylidene)piperidin-4′-one* (**4j**). Obtained as a pale yellow solid, (0.129 g, 85%); mp = 187–188 °C; IR (KBr): 1600, 1615, 1706, 3410 cm^−^^1^; ^1^H-NMR (400 MHz, CDCl_3_): δ_H _2.14 (s, 3H, N-CH_3_), 2.21 (d, 1H, *J =* 13.2 Hz, 2'-CH_2_), 3.35 (dd, 1H, *J =* 7.2, 7.2 Hz, 5-CH_2_), 3.55–3.76 (m, 2H, 6'-CH_2_), 3.77 (s, 3H, OCH_3_), 3.78 (s, 3H, OCH_3_), 3.80–3.91 (m, 2H, 5-CH_2 _and 2'-CH_2_), 4.78 (dd, 1H, *J =* 7.2, 7.2 Hz, 4-CH), 6.65 (d, 1H, *J =* 7.6 Hz, Ar-H), 6.77–7.09 (m, 8H, Ar-H), 7.13 (d, 2H, *J =* 7.6 Hz, Ar-H), 7.35 (d, 2H, *J =* 8.8 Hz, Ar-H), 8.27 (s, 1H, 1"-NH). ^13^C-NMR (100 MHz, CDCl_3_): δ_C_ 34.61, 45.52, 48.36, 49.82, 55.18, 55.22, 57.37, 65.85, 76.12, 108.86, 113.60, 113.74, 122.05, 127.61, 127.90, 128.71, 130.58, 131.84, 132.37, 132.72, 137.27, 141.66, 158.45, 159.97, 178.22, 199.52. Anal. calcd for C_31_H_31_N_3_O_4_: C, 73.06; H, 6.13; N, 8.25; found: C, 73.17; H, 6.01; N, 8.34.

*1-Methyl-4-(4-chlorophenyl)pyrrolo-(spiro[2.3″]oxindole)-spiro[3.3′]-5′-(4-chlorophenylmethylidene)piperidin-4′-one* (**4k**). Obtained as a pale yellow solid, (0.140 g, 93%); mp = 169–170°C; IR (KBr): 1602, 1617, 1709, 3410 cm^−^^1^; ^1^H-NMR (500 MHz, CDCl_3_): δ_H_ 2.07–2.10 (m, 1H, 2'-CH_2_), 2.13 (s, 3H, N-CH_3_), 3.34 (t, 1H, *J =* 8.0 Hz, 5-CH_2_), 3.48–3.54 (m, 2H, 6'-CH_2_), 3.60 (d, 1H, *J =* 13.0 Hz, 2'-CH_2_), 3.86 (t, 1H, *J =* 10.0 Hz, 5-CH_2_), 4.79 (dd, 1H, *J =* 7.5, 7.5 Hz, 4-CH), 6.67 (d, 1H, *J =* 8.0 Hz, Ar-H), 6.91 (d, 2H, *J =* 8.0 Hz, Ar-H), 6.97 (t, 1H, *J =* 7.5 Hz, Ar-H), 7.00 (s, 1H, C=CH), 7.07–7.13 (m, 2H, Ar-H), 7.21–7.28 (m, 4H, Ar-H), 7.35 (d, 2H, *J =* 8.5 Hz, Ar-H), 8.45 (s, 1H, 1"-NH). ^13^C-NMR (125 MHz, CDCl_3_): δ_C_ 34.60, 45.32, 48.21, 49.92, 56.99, 66.17, 75.81, 109.17, 122.15, 127.81, 128.42, 128.53, 128.78, 129.00, 130.97, 131.07, 132.69, 133.50, 134.71, 134.98, 136.16, 137.00, 141.77, 178.30, 199.24. Anal. calcd for C_29_H_25_Cl_2_N_3_O_2_: C, 67.19; H, 4.86; N, 8.11; found: C, 67.07; H, 4.73; N, 8.19.

*1-Methyl-4-(4-bromophenyl)pyrrolo-(spiro[2.3″]oxindole)-spiro[3.3′]-5′-(4-bromophenylmethylidene)piperidin-4′-one* (**4l**). Obtained as a pale yellow solid, (0.127 g, 91%); mp = 172–173 °C; IR (KBr): 1598, 1619, 1710, 3409 cm^−^^1^; ^1^H-NMR (300 MHz, MeOH): δ_H_ 2.11 (s, 3H, N-CH_3_), 2.17 (d, 1H, *J =* 13.2 Hz, 2'-CH_2_), 3.32–3.56 (m, 4H, 5-CH_2_,6'-CH_2 _and 2'-CH_2_), 3.90 (dd, 1H, *J =* 9.0, 9.0 Hz, 5-CH_2_), 4.64 (s, 1H, 1"-NH), 4.76 (dd, 1H, *J =* 7.5, 7.2 Hz, 4-CH), 6.72 (d, 1H, *J =* 7.8 Hz, Ar-H), 6.98–7.18 (m, 6H, Ar-H), 7.34 (d, 2H, *J =* 8.4 Hz, Ar-H), 7.45–7.48 (m, 4H, Ar-H). ^13^C-NMR (75 MHz, MeOH): δ_C_ 33.86, 45.84, 48.28, 50.01, 57.10, 65.86, 76.31, 109.55, 120.81, 121.91, 122.96, 127.15, 127.74, 129.36, 131.43, 131.56, 131.69, 131.77, 134.43, 135.74, 136.13, 138.06, 143.25, 178.53, 199.78. Anal. calcd for C_29_H_25_Br_2_N_3_O_2_: C, 57.35; H, 4.15; N, 6.92; found: C, 57.48; H, 4.27; N, 6.99.

*1-Methyl-4-(4-fluorophenyl)pyrrolo-(spiro[2.3″]oxindole)-spiro[3.3′]-5′-(4-fluorophenylmethylidene)piperidin-4′-one* (**4m**). Obtained as a white solid, (0.146 g, 94%); mp = 174–175 °C; IR (KBr): 1601, 1618, 1709, 3411 cm^−^^1^; ^1^H-NMR (300 MHz, MeOH): δ_H_ 2.10 (s, 3H, N-CH_3_), 2.18 (d, 1H, *J =* 13.2 Hz, 2'-CH_2_), 3.26–3.57 (m, 4H, 5-CH_2_, 6'-CH_2 _and 2'-CH_2_), 3.89 (dd, 1H, *J =* 9.3, 9.0 Hz, 5-CH_2_), 4.65 (s, 1H, 1"-NH), 4.78 (dd, 1H, *J =* 7.5, 7.2 Hz, 4-CH), 6.73 (d, 1H, *J =* 7.5 Hz, Ar-H), 6.89–7.17 (m, 11H, Ar-H), 7.39–7.44 (m, 1H, Ar-H). ^13^C-NMR (75 MHz, MeOH): δ_C_ 33.90, 45.73, 48.22, 50.02, 57.40, 65.75, 76.42, 109.54, 114.64 (^2^*J*_CF_ = 21.2 Hz), 115.30 (^2^*J*_CF_ = 21.8 Hz), 121.93, 127.22, 127.75, 129.32, 131.30 (*J*_CF_ = 77.3 Hz), 131.73 (*J*_CF_ = 3.3 Hz), 132.25 (*J*_CF_ = 83.3 Hz), 134.64 (^2^*J*_CF_ = 3.2 Hz), 134.87, 136.42, 143.22, 162.32 (^1^*J*_CF_ = 242.7 Hz), 163.14 (^1^*J*_CF_ = 247.4 Hz), 178.58, 199.98. Anal. calcd for C_29_H_25_F_2_N_3_O_2_: C, 71.74; H, 5.19; N, 8.65; found: C, 71.87; H, 5.31; N, 8.59.

*1-Methyl-4-(1-naphthyl)pyrrolo-(spiro[2.3″]oxindole)-spiro[3.3′]-5′-(1-naphthylmethylidene)piperidin-4′-one* (**4n**). Obtained as a pale yellow solid, (0.132 g, 90%); mp = 160–161 °C; IR (KBr): 1698, 1620, 1706, 3410 cm^−1^; ^1^H-NMR (300 MHz, DMSO): δ_H_ 1.66 (d, 1H, *J =* 12.6 Hz, 2'-CH_2_), 2.00 (s, 3H, N-CH_3_), 3.07 (d, 1H, *J =* 15.8 Hz, 6'-CH_2_), 3.19 (d, 1H, *J =* 15.8 Hz, 6'-CH_2_), 3.35 (t, 1H, *J =* 7.8 Hz, 5-CH_2_), 3.55 (d, 1H, *J =* 12.6 Hz, 2'-CH_2_), 4.05 (t, 1H, *J =* 9.3 Hz, 5-CH_2_), 5.50 (t, 1H, *J =* 8.4 Hz, 4-CH), 6.66 (d, 1H, *J =* 6.6 Hz, Ar-H), 7.02–7.96 (m, 17H, Ar-H), 8.12 (d, 1H, *J =* 8.7 Hz, Ar-H), 10.46 (s, 1H, 1"-NH). ^13^C-NMR (75 MHz, DMSO): δ_C_ 34.95, 40.63, 49.23, 51.52, 58.72, 64.93, 76.59, 109.95, 122.19, 124.48, 125.49, 125.89, 126.17, 126.33, 127.12, 127.32, 127.47, 127.61, 127.79, 128.03, 129.23, 129.36, 129.56, 129.74, 129.80, 131.64, 132.54, 133.63, 133.74, 134.37, 135.14, 135.84, 137.0, 144.41, 177.57, 199.76. Anal. calcd for C_37_H_31_N_3_O_2_: C, 80.85; H, 5.68; N, 7.64; found: C, 80.96; H, 5.82; N, 7.55.

*4,5-Diphenylpyrrolo(spiro[2.3′′]-oxindole)-spiro-[3.3′]-5′-(phenylmethylidene)piperidin-4′-one* (**5a**). Obtained as a white solid, (0.170 g, 93%); mp = 162–163 °C; IR (KBr): 1593, 1613, 1698, 3179, 3344 cm^−^^1^; ^1^H-NMR (500 MHz, CDCl_3_): δ_H_ 2 .31 (d, 1H, *J =* 13.0 Hz, 2'-CH_2_), 3.52 (dd, 1H, *J =* 14.5, 2.5 Hz, 6'-CH_2_), 3.64 (d, 1H, *J =* 15.0 Hz, 6'-CH_2_), 3.77 (dd, 1H, *J =* 13.0, 1.0 Hz, 2'-CH_2_), 4.73 (d, 1H, *J =* 11.0 Hz, 4-CH), 5.43 (d, 1H, *J =* 11.0 Hz, 5-CH), 6.67 (d, 1H, *J =* 7.5 Hz, Ar-H), 6.98–7.04 (m, 4H, Ar-H), 7.12–7.28 (m, 11H, Ar-H), 7.41 (d, 2H, *J =* 7.0 Hz, Ar-H), 7.53 (d, 2H, *J =* 7.5 Hz, Ar-H), 7.99 (s, 1H, 1"-NH). ^13^C-NMR (125 MHz, CDCl_3_): δ_C_48.19, 49.74, 56.85, 64.24, 67.34, 71.82, 109.21, 122.20, 126.85, 127.02, 127.57, 127.66, 128.25, 128.33, 128.70, 128.99, 129.21, 129.89, 129.93, 134.86, 135.12, 137.09, 137.38, 140.74, 141.00, 180.91, 200.14. Anal. calcd for C_34_H_29_N_3_O_2_: C, 79.82; H, 5.71; N, 8.21; found: C, 79.70; H, 5.85; N, 8.15.

*4-(2-Methylphenyl)-5-phenylpyrrolo(spiro[2.3″]oxindole)spiro[3.3′]-5′-(2-methylphenylmethylidene) piperidin-4′-one* (**5b**). Obtained as a pale yellow solid, (0.160 g, 90%); mp = 150–151 °C; IR (KBr): 1595, 1618, 1697, 3168, 3340 cm^−^^1^; ^1^H-NMR (300 MHz, CDCl_3_): δ_H_ 2.11 (s, 1H, CH_3_), 2.20 (s, 1H, CH_3_), 2.27–2.32 (m, 1H, 2'-CH_2_), 3.41 (d, 1H, *J =* 15.3 Hz, 6'-CH_2_), 3.51 (dd, 1H, *J =* 15.3, 2.4 Hz, 6'-CH_2_), 3.58 (d, 1H, *J =* 13.2 Hz, 2'-CH_2_), 4.90 (d, 1H, *J =* 9.9 Hz, 4-CH), 5.54 (d, 1H, *J =* 9.9 Hz, 5-CH), 6.73 (d, 1H, *J =* 7.5 Hz, Ar-H), 6.82 (d, 1H, *J =* 7.5 Hz, Ar-H), 6.92–7.55 (m, 15H, Ar-H), 7.63 (s, 1H, C=CH), 8.42 (s, 1H, 1"-NH). ^13^C-NMR (75 MHz, CDCl_3_): δ_C_ 20.49, 21.44, 48.43, 51.42, 54.54, 65.44, 66.53, 74.25, 109.94, 122.91, 125.80, 125.98, 126.29, 126.98, 127.83, 127.91, 128.81, 128.94, 129.42, 129.64, 130.58, 130.78, 134.36, 134.54, 135.46, 135.60, 136.82, 137.44, 138.21, 138.31, 138.63, 141.79, 180.90, 201.06. Anal. calcd for C_36_H_33_N_3_O_2_: C, 80.12; H, 6.16; N, 7.79; found: C, 80.28; H, 6.27; N, 7.70.

*4-(2-Methoxyphenyl)-5-phenylpyrrolo(spiro[2.3″]oxindole)spiro[3.3′]-5′-(2-methoxyphenylmethylidene)piperidin-4′-one* (**5c**). Obtained as a pale yellow solid, (0.144 g, 85%); mp = 155–156 °C; IR (KBr): 1597, 1614, 1695, 3173, 3342 cm^−^^1^; ^1^H-NMR (300 MHz, CDCl_3_): δ_H_ 2.43 (d, 1H, *J =* 13.8 Hz, 2'-CH_2_), 3.26–3.62 (m, 3H, 6'-CH_2_ and 2'-CH_2_), 3.65 (s, 3H, OCH_3_), 3.83 (s, 3H, OCH_3_), 4.89 (d, 1H, *J =* 9.9 Hz, 4-CH), 5.74 (d, 1H, *J =* 9.9 Hz, 5-CH), 6.67–7.86 (m, 18H, Ar-H and C=CH), 8.02 (s, 1H, 1"-NH). ^13^C-NMR (75 MHz, CDCl_3_): δ_C_ 48.50, 49.76, 54.32, 55.41, 55.80, 63.89, 66.56, 74.29, 110.36, 111.05, 111.12, 120.31, 120.51, 121.09, 122.83, 124.72, 127.33, 127.88, 128.07, 128.31, 128.85, 129.29, 130.53, 130.88, 131.03, 133.42, 141.81, 158.23, 158.76, 180.01, 201.03. Anal. calcd for C_36_H_33_N_3_O_4_: C, 75.64; H, 5.82; N, 7.35; found: C, 75.50; H, 5.97; N, 7.18.

*4-(2-Chlorophenyl)-5-phenylpyrrolo(spiro[2.3″]oxindole)spiro[3.3′]-5′-(2-chlorophenylmethylidene)piperidin-4′-one* (**5d**). Obtained as a white solid, (0.155 g, 92%); mp = 151–152 °C; IR (KBr): 1590, 1617, 1692, 3175, 3340 cm^−^^1^; ^1^H-NMR (300 MHz, CDCl_3_): δ_H _1.97 (br s, 1H, NH), 2.41 (d, 1H, *J =* 13.8 Hz, 2'-CH_2_), 3.27 (d, 1H, *J =* 13.8 Hz, 2'-CH_2_), 3.42 (d, 1H, *J =* 15.6 Hz, 6'-CH_2_), 3.61 (d, 1H, *J =* 15.6 Hz, 6'-CH_2_), 5.11 (d, 1H, *J =* 9.0 Hz, 4-CH), 5.66 (d, 1H, *J =* 9.0 Hz, 5-CH), 6.69 (d, 1H, *J =* 7.5 Hz, Ar-H), 6.97–7.39 (m, 12H, Ar-H), 7.58–7.66 (m, 3H, Ar-H), 7.84 (s, 1H, C=CH), 8.15 (d, 1H, *J =* 7.2 Hz, Ar-H), 8.68 (s, 1H, 1"-NH). ^13^C-NMR (75 MHz, CDCl_3_): δ_C_ 48.52, 50.71, 55.41, 64.74, 65.74, 75.18, 109.92, 123.44, 126.66, 127.17, 127.29, 127.72, 128.12, 128.18, 128.47, 129.00, 129.51, 129.71, 130.19, 130.32, 130.52, 131.09, 133.87, 135.11, 135.33, 135.58, 136.52, 136.70, 141.22, 141.64, 179.14, 200.63. Anal. calcd for C_34_H_27_Cl_2_N_3_O_2_: C, 70.35; H, 4.69; N, 7.24; found: C, 70.47; H, 4.61; N, 7.16.

*4-(2-Bromophenyl)-5-phenylpyrrolo(spiro[2.3″]oxindole)spiro[3.3′]-5′-(2-bromophenylmethylidene)piperidin-4′-one* (**5e**). Obtained as a white solid, (0.137 g, 89%); mp = 153–154 °C; IR (KBr): 1590, 1611, 1694, 3184, 3337 cm^−^^1^; ^1^H-NMR (300 MHz, CDCl_3_): δ_H_ 1.91 (br s, 1H, NH), 2.45 (d, 1H, *J =* 13.8 Hz, 2'-CH_2_), 3.23 (d, 1H, *J =* 13.8 Hz, 2'-CH_2_), 3.40 (d, 1H, *J =* 15.6 Hz, 6'-CH_2_), 3.62 (dd, 1H, *J =* 15.6, 2.1 Hz, 6'-CH_2_), 5.06 (d, 1H, *J =* 9.3 Hz, 4-CH), 5.67 (d, 1H, *J =* 9.3 Hz, 5-CH), 6.71 (d, 1H, *J =* 7.8 Hz, Ar-H), 6.95–7.44 (m, 8H, Ar-H), 7.48 (d, 2H, *J =* 7.5 Hz, Ar-H), 7.58 (d, 2H, *J =* 7.2 Hz, Ar-H), 7.67 (d, 2H, *J =* 7.2 Hz, Ar-H), 7.84 (s, 1H, C=CH), 8.21(d, 1H, *J =* 7.8 Hz, Ar-H), 8.67 (s, 1H, 1"-NH). ^13^C-NMR (75 MHz, CDCl_3_): δ_C_ 48.28, 50.66, 58.56, 64.64, 66.37, 75.47, 110.01, 123.57, 125.76, 127.16, 127.32, 127.59, 127.93, 128.13, 128.22, 128.54, 128.83, 129.04, 129.55, 130.54, 131.49, 133.07, 133.45, 135.14, 135.62, 137.38, 138.44, 141.13, 141.68, 178.95, 200.93. Anal. calcd for C_34_H_27_Br_2_N_3_O_2_: C, 61.00; H, 4.07; N, 6.28; found: C, 61.13; H, 4.26; N, 6.40.

*4-(2-Fluorophenyl)-5-phenylpyrrolo(spiro[2.3″]oxindole)spiro[3.3′]-5′-(2-fluorophenylmethylidene)piperidin-4′-one* (**5f**). Obtained as a white solid, (0.158 g, 90%); mp = 148–149 °C; IR (KBr): 1592, 1618, 1694, 3178, 3345 cm^−^^1^; ^1^H-NMR (300 MHz, CDCl_3_): δ_H_ 2.20 (br s, 1H, NH), 2.43 (d, 1H, *J =* 13.5 Hz, 2'-CH_2_), 3.31–3.66 (m, 3H, 2'-CH_2 _and 6'-CH_2_), 5.00 (d, 1H, *J =* 9.9 Hz, 4-CH), 5.57 (d, 1H, *J =* 9.9 Hz, 5-CH), 6.68 (d, 1H, *J =* 7.5 Hz, Ar-H), 6.90–7.63 (m, 16H, Ar-H), 7.87 (s, 1H, C=CH), 8.65 (s, 1H, 1"-NH). ^13^C-NMR (75 MHz, CDCl_3_): δ_C_ 48.79, 50.39, 63.10, 64.48, 65.97, 73.65, 109.92, 115.64 (^2^*J*_CF_ = 23.3 Hz), 116.08 (^2^*J*_CF_ = 21.7 Hz), 122.94, 123.39, 123.57, 124.07 (*J*_CF_ = 3.0 Hz), 124.48 (*J*_CF_ = 3.0 Hz), 127.28, 128.01, 128.18, 128.31, 128.92, 129.41, 129.56, 130.16, 130.61, 130.80, 131.16, 136.04, 140.98, 141.59, 161.15 (^1^*J*_CF_ = 250.6 Hz), 161.96 (^1^*J*_CF_ = 244.1 Hz), 180.24, 199.77. Anal. calcd for C_34_H_27_F_2_N_3_O_2_: C, 74.57; H, 4.97; N, 7.67; found: C, 74.45; H, 4.88; N, 7.74.

*4-(2,4-Dichlorophenyl)-5-phenylpyrrolo(spiro[2.3″]oxindole)spiro[3.3′]-5′-(2,4-dichlorophenyl methylidenedichlorophenylmethylidene)piperidin-4′-one* (**5g**). Obtained as a white solid, (0.144 g, 92%); mp = 160–161 °C; IR (KBr): 1599, 1618, 1704, 3182, 3338 cm^−^^1^; ^1^H-NMR (300 MHz, CDCl_3_): δ_H_ 2.35 (d, 1H, *J =* 13.5 Hz, 2'-CH_2_), 3.23–3.42 (m, 2H, 2'-CH_2 _and 6'-CH_2_), 3.56 (d, 1H, *J =* 15.3 Hz, 6'-CH_2_), 5.04 (d, 1H, *J =* 9.3 Hz, 4-CH), 5.58 (d, 1H, *J =* 9.3 Hz, 5-CH), 6.69 (d, 1H, *J =* 7.2 Hz, Ar-H), 6.88 (d, 1H, *J =* 8.1 Hz, Ar-H), 6.97 (d, 1H, *J =* 7.5 Hz, Ar-H), 7.12–7.29 (m, 5H, Ar-H), 7.40 (s, 1H, Ar-H), 7.54 (d, 2H, *J =* 7.2 Hz, Ar-H), 7.61 (d, 2H, *J =* 7.8 Hz, Ar-H), 7.76 (s, 1H, C=CH), 7.88 (d, 1H, *J =* 7.2 Hz, Ar-H), 8.09 (d, 1H, *J =* 8.1 Hz, Ar-H), 8.90 (s, 1H, 1"-NH). ^13^C-NMR (75 MHz, CDCl_3_): δ_C_ 48.49, 50.62, 54.85, 64.54, 65.89, 75.09, 110.16, 123.42, 127.13, 127.47, 127.64, 128.02, 128.43, 129.12, 129.42, 129.52, 130.18, 131.16, 131.93, 132.21, 133.64, 134.12, 134.92, 135.27, 135.51, 135.71, 136.34, 137.08, 140.77, 141.75, 179.13, 200.24. Anal. calcd for C_34_H_25_Cl_4_N_3_O_2_: C, 62.88; H, 3.88; N, 6.47; found: C, 62.75; H, 3.97; N, 6.40.

**4***-(3-Nitrophenyl)-5-phenylpyrrolo(spiro[2.3″]oxindole)spiro[3.3′]-5′-(3-nirophenylmethylidene) piperidin-4′-one* (**5h**). Obtained as a white solid, (0.149 g, 91%); mp = 154–155 °C; IR (KBr): 1596, 1612, 1702, 3186, 3336 cm^−^^1^; ^1^H-NMR (300 MHz, CDCl_3_): δ_H_ 2.26 (d, 1H, *J =* 12.9 Hz, 2'-CH_2_), 3.43–3.85 (m, 3H, 2'-CH_2 _and 6'-CH_2_), 4.78 (d, 1H, *J =* 10.5 Hz, 4-CH), 5.43 (d, 1H, *J =* 10.5 Hz, 5-CH), 6.70 (m, 11H, Ar-H), 7.80 (s, 1H, C=CH), 7.87–8.13 (m, 4H, Ar-H), 8.20 (s, 1H, Ar-H), 8.35 (s, 1H, Ar-H), 8.76 (s, 1H, 1"-NH). ^13^C-NMR (75 MHz, CDCl_3_): δ_C_ 48.33, 56.82, 63.88, 65.01, 67.37, 72.16, 110.16, 122.62, 123.71, 124.55, 125.02, 127.31, 127.95, 128.50, 129.01, 129.42, 129.71, 129.84, 130.17, 134.77, 135.81, 136.76, 136.88, 137.20, 140.01, 140.29, 141.81, 148.46, 148.64, 181.44, 199.69. Anal. calcd for C_34_H_27_N_5_O_6_: C, 67.88; H, 4.52; N, 11.64; found: C, 67.73; H, 4.73; N, 11.46.

*4-(4-Methylphenyl)-5-phenylpyrrolo(spiro[2.3″]oxindole)spiro[3.3′]-5′-(4-methylphenylmethylidene) piperidin-4′-one* (**5i**). Obtained as a pale yellow solid, (0.164 g, 92%); mp = 157–158 °C; IR (KBr): 1597, 1615, 1690, 3181, 3340 cm^−^^1^; ^1^H-NMR (300 MHz, CDCl_3_): δ_H_ 2.25 (s, 1H, CH_3_), 2.29 (s, 1H, CH_3_), 2.34 (d, 1H, *J =* 13.2 Hz, 2'-CH_2_), 3.50 (d, 1H, *J =* 15.0 Hz, 6'-CH_2_), 3.60 (d, 1H, *J =* 15.0 Hz, 6'-CH_2_), 3.70 (d, 1H, *J =* 13.2 Hz, 2'-CH_2_), 4.68 (d, 1H, *J =* 10.5 Hz, 4-CH), 5.40 (d, 1H, *J =* 10.5 Hz, 5-CH), 6.65 (d, 1H, *J =* 7.5 Hz, Ar-H), 6.76 (d, 1H, *J =* 7.8 Hz, Ar-H), 6.91 (d, 2H, *J =* 8.1 Hz, Ar-H), 6.97–7.30 (m, 12H, Ar-H), 7.52 (d, 2H, *J =* 6.9 Hz, Ar-H), 8.72 (s, 1H, 1"-NH). ^13^C-NMR (75 MHz, CDCl_3_): δ_C_ 21.49, 21.77, 48.63, 50.03, 57.33, 64.90, 67.44, 72.62, 109.86, 122.53, 127.31, 127.95, 128.11, 128.76, 129.24, 129.42, 129.46, 130.17, 130.58, 132.73, 134.46, 134.73, 135.52, 136.76, 137.66, 139.52, 141.30, 141.69, 181.55, 200.83. Anal. calcd for C_36_H_33_N_3_O_2_: C, 80.12; H, 6.16; N, 7.79; found: C, 80.03; H, 6.24; N, 7.85.

*4-(4-Methoxyphenyl)-5-phenylpyrrolo(spiro[2.3″]oxindole)spiro[3.3′]-5′-(4-methoxyphenylmethyl idene)piperidin-4′-one* (**5j**). Obtained as a pale yellow solid, (0.146 g, 86%); mp = 159–160 °C; IR (KBr): 1598, 1620, 1710, 3179, 3347 cm^−^^1^; ^1^H-NMR (300 MHz, CDCl_3_): δ_H_ 2.44 (d, 1H, *J =* 13.8 Hz, 2'-CH_2_), 3.28–3.63 (m, 3H, 6'-CH_2_ and 2'-CH_2_), 3.66 (s, 3H, CH_3_), 3.85 (s, 3H, OCH_3_), 4.89 (d, 1H, *J =* 9.9 Hz, 4-CH), 5.74 (d, 1H, *J =* 9.9 Hz, 5-CH), 6.69–7.87 (m, 18H, Ar-H), 8.20 (s, 1H, 1"-NH). ^13^C-NMR (75 MHz, CDCl_3_): δ_C_ 48.53, 50.07, 55.42, 55.79, 57.46, 64.93, 67.51, 72.69, 110.35, 111.05, 111.11, 120.30, 120.50, 121.09, 122.86, 124.74, 127.38, 127.87, 128.07, 128.31, 128.84, 129.41, 130.52, 130.85, 131.03, 141.81, 158.52, 158.76, 181.22, 199.93. Anal. calcd for C_36_H_33_N_3_O_4_: C, 75.64; H, 5.82; N, 7.35; found: C, 75.78; H, 5.93; N, 7.42.

*4-(4-Chlorophenyl)-5-phenylpyrrolo(spiro[2.3″]oxindole)spiro[3.3′]-5′-(4-chlorophenylmethylidene)piperidin-4′-one* (**5k**). Obtained as a pale yellow solid, (0.151 g, 90%); mp = 154–155 °C; IR (KBr): 1598, 1618, 1711, 3179, 3338 cm^−^^1^; ^1^H-NMR (300 MHz, CDCl_3_): δ_H_ 2.09 (s, 1H, NH), 2.27 (d, 1H, *J =* 13.2 Hz, 2'-CH_2_), 3.46–3.60 (m, 2H, 6'-CH_2_), 3.72 (d, 1H, *J =* 13.2 Hz, 2'-CH_2_), 4.67 (d, 1H, *J =* 10.8 Hz, 4-CH), 5.35 (d, 1H, *J =* 10.8 Hz, 5-CH), 6.67 (d, 1H, *J =* 7.2 Hz, Ar-H), 6.77 (d, 1H, *J =* 7.2 Hz, Ar-H), 6.90–7.54 (m, 16H, Ar-H), 8.49 (s, 1H, 1"-NH). ^13^C-NMR (75 MHz, CDCl_3_): δ_C_ 48.52, 50.03, 56.65, 64.81, 67.43, 72.25, 109.85, 122.58, 124.35, 127.32, 127.98, 128.34, 128.76, 128.87, 129.00, 129.31, 129.53, 131.57, 132.07, 133.16, 133.79, 135.25, 135.42, 136.33, 140.72, 141.61, 181.42, 200.18. Anal. calcd for C_34_H_27_Cl_2_N_3_O_2_: C, 70.35; H, 4.69; N, 7.24; found: C, 70.51; H, 4.80; N, 7.31.

*4-(4-Bromophenyl)-5-phenylpyrrolo(spiro[2.3″]oxindole)spiro[3.3′]-5′-(4-bromophenylmethylidene)pipe*ridin-4′-one (**5l**). Obtained as a white solid, (0.142 g, 92%); mp = 166–167 °C; IR (KBr): 1597, 1615, 1705, 3174, 3340 cm^−^^1^; ^1^H-NMR (300 MHz, CDCl_3_): δ_H_ 2.08 (s, 1H, NH), 2.27 (d, 1H, *J =* 12.9 Hz, 2'-CH_2_), 3.46–3.79 (m, 3H, 6'-CH_2_ and 2'-CH_2_), 4.66 (d, 1H, *J =* 10.8 Hz, 4-CH), 5.34 (d, 1H, *J =* 10.8 Hz, 5-CH), 6.66–6.72 (m, 2H, Ar-H), 6.84 (d, 2H, *J =* 8.1 Hz, Ar-H), 6.93 (s, 1H, C=CH), 6.97–7.51 (m, 13H, Ar-H), 8.39 (s, 1H, 1"-NH). ^13^C-NMR (75 MHz, CDCl_3_): δ_C_ 48.52, 50.03, 56.67, 64.73, 67.43, 72.21, 109.84, 121.36, 122.59, 123.59, 127.98, 128.24, 128.88, 129.54, 129.77, 130.18, 131.77, 131.85, 131.97, 132.27, 134.23, 135.53, 136.37, 136.79, 140.69, 141.58, 181.33, 200.14. Anal. calcd for C_34_H_27_Br_2_N_3_O_2_: C, 61.00; H, 4.07; N, 6.28; found: C, 61.17; H, 4.21; N, 6.35.

*4-(4-Fluorophenyl)-5-phenylpyrrolo(spiro[2.3″]oxindole)spiro[3.3′]-5′-(4-fluorophenylmethylidene) piperidin-4′-one* (**5m**). Obtained as a pale yellow solid, (0.164 g, 93%); mp = 159–160 °C; IR (KBr): 1596, 1617, 1700, 3181, 3346 cm^−1^; ^1^H-NMR (300 MHz, CDCl_3_): δ_H_ 2.13 (s, 1H, NH), 2.28 (d, 1H,*J =* 13.2 Hz, 2'-CH_2_), 3.48 (d, 1H, *J =* 14.7 Hz, 6'-CH_2_), 3.58 (d, 1H, *J =* 14.7 Hz, 6'-CH_2_), 3.70 (d, 1H, *J =* 13.2 Hz, 2'-CH_2_), 4.67 (d, 1H, *J =* 10.5 Hz, 4-CH), 5.34 (d, 1H, *J =* 10.5 Hz, 5-CH), 6.68 (d, 2H, *J =* 7.5 Hz, Ar-H), 6.89–7.55 (m, 16H, Ar-H), 8.58 (s, 1H, 1"-NH). ^13^C-NMR (75 MHz, CDCl_3_): δ_C_ 48.50, 50.05, 56.79, 65.10, 67.27, 72.40, 109.86, 115.56 (^2^*J*_CF_ = 21.0 Hz), 115.88 (^2^*J*_CF_ = 21.5 Hz), 122.56, 127.33, 127.99, 128.15, 128.83, 129.41, 129.46, 130.17, 131.76, 132.30, 133.43, 134.77, 136.55, 140.93, 141.65, 162.23 (^1^*J*_CF _= 244.1 Hz), 163.13 (^1^*J*_CF _= 248.9 Hz), 181.43, 200.42. Anal. calcd for C_34_H_27_F_2_N_3_O_2_: C, 74.57; H, 4.97; N, 7.67; found: C, 74.69; H, 4.90; N, 7.76.

*4-(1-Naphthyl)-5-phenylpyrrolo(spiro[2.3″]oxindole)spiro[3.3′]-5′-(1-naphthylmethylidene)piperidin-4′-one* (**5n**). Obtained as a yellow solid, (0.142 g, 87%); mp = 164–165 °C; IR (KBr): 1590, 1619, 1704, 3174, 3335 cm^−1^; ^1^H-NMR (300 MHz, CDCl_3_): δ_H_ 1.94 (s, 1H, NH), 2.07 (d, 1H, *J =* 12.9 Hz, 2'-CH_2_), 3.18–3.73 (m, 3H, 6'-CH_2_ and 2'-CH_2_), 5.58 (d, 1H, *J =* 9.9 Hz, 4-CH), 5.80 (d, 1H, *J =* 9.9 Hz, 5-CH), 6.71–8.06 (m, 24H, Ar-H), 8.50 (s, 1H, 1"-NH). ^13^C-NMR (75 MHz, CDCl_3_): δ_C_ 48.70, 50.15, 56.53, 64.71, 67.46, 72.20, 109.83, 122.96, 124.46, 125.04, 125.41, 126.45, 126.73, 127.12, 127.24, 127.58, 127.84, 127.98, 128.61, 128.82, 129.05, 129.20, 129.41, 129.99, 130.16, 132.41, 132.64, 133.24, 133.84, 134.84, 135.15, 135.62, 136.91, 137.01, 144.40, 181.38, 200.13. Anal. calcd for C_42_H_33_N_3_O_2_: C, 82.46; H, 5.44; N, 6.87; found: C, 82.39; H, 5.56; N, 6.80.

*Spiro[2.3″]oxindole-spiro[3.3′]-5′-(phenylmethylidene)-tetrahydro-4′(1H)-pyridinone-4-(phenyl) hexahydro-1H-pyrrolizine* (**6a**). Obtained as a white solid, (0.165 g, 94%); mp = 182–188 °C; IR (KBr): 1610, 1618, 1706, 3390 cm^−1^; ^1^H-NMR (400 MHz, CDCl_3_): δ_H_ 1.60–1.69 (m, 1H, 5-CH_2_), 1.82–2.01 (m, 2H, 6-CH_2_), 2.04–2.14 (m, 1H, 5-CH_2_), 2.33 (d, 1H, *J =* 13.2 Hz, 2'-CH_2_), 2.60 (td, 1H, *J =* 8.8, 2.4 Hz, 7-CH_2_), 3.07–3.13 (m, 1H, 7-CH_2_), 3.39 (d, 1H, *J =* 14.8 Hz, 6'-CH_2_), 3.62 (d, 1H, *J =* 14.8 Hz, 6'-CH_2_), 3.96 (d, 1H, *J =* 13.2 Hz, 2'-CH_2_), 4.38 (d, 1H, *J =* 11.2 Hz, 4-CH), 4.62–4.70 (m, 1H, 4a-CH), 6.73 (d, 1H, *J =* 6.8 Hz, Ar-H), 6.94–7.02 (m, 3H, Ar-H), 7.15–7.31 (m, 9H, Ar-H and arylmethylidene), 7.37 (d, 2H, *J =* 7.6 Hz, Ar-H), 8.87 (s, 1H, 1"-NH). ^13^C-NMR (100 MHz, CDCl_3_): δ_C_ 26.28, 29.29, 48.13, 48.35, 49.30, 53.64, 66.52, 71.88, 74.81, 109.97, 121.78, 127.23, 128.62, 128.69, 128.93, 129.36, 129.39, 130.03, 130.14, 135.58, 136.18, 136.30, 137.95, 142.06, 180.06, 200.63. Anal. calcd for C_31_H_29_N_3_O_2_: C, 78.29; H, 6.15; N, 8.84%; found: C, 78.12; H, 6.33; N, 8.95%.

*Spiro[2.3″]oxindole-spiro[3.3′]-5′-(2-methylphenylmethylidene)tetrahydro-4′(1H)-pyridinone-4-(2-methylphenyl)hexahydro-1H-pyrrolizine* (**6b**). Obtained as a white solid, (0.154 g, 93%); mp = 184–185 °C; IR (KBr): 1605, 1622, 1705, 3387 cm^−1^; ^1^H-NMR (300 MHz, CDCl_3_): δ_H_ 1.58–1.70 (m, 1H, 5-CH_2_), 1.78–2.04 (m, 3H, 6-CH_2 _and 5-CH_2_), 2.30 (s, 3H, CH_3_), 2.31 (s, 3H, CH_3_), 2.42 (d, 1H, *J =* 12.9 Hz, 2'-CH_2_), 2.64 (td, 1H, *J =* 8.1, 3.0 Hz, 7-CH_2_), 3.09–3.17 (m, 1H, 7-CH_2_), 3.61 (dd, 1H, *J =* 15.0, 2.1 Hz, 6'-CH_2_), 3.97 (d, 1H, *J =* 15.0 Hz, 6'-CH_2_), 4.19 (d, 1H, *J =* 12.9 Hz, 2'-CH_2_), 4.62 (d, 1H, *J* = 10.8 Hz, 4-CH), 4.82–4.86 (m, 1H, 4a-CH), 6.62 (d, 1H, *J =* 7.5 Hz, Ar-H), 6.87–7.25 (m, 10H, Ar-H), 7.53–7.63 (m, 2H, Ar-H and arylmethylidene), 8.03 (s, 1H, 1"-NH). ^13^C-NMR (75 MHz, CDCl_3_): δ_C_ 20.39, 21.12, 26.08, 28.59, 47.19, 48.92, 49.22, 53.22, 65.83, 70.05, 74.73, 110.12, 122.47, 125.78, 125.95, 127.34, 128.61, 129.30, 129.42, 129.59, 130.62, 130.80, 131.13, 133.45, 134.52, 135.11, 136.61, 138.26, 138.77, 141.61, 180.74, 202.27. Anal. calcd for C_33_H_33_N_3_O_2_: C, 78.70; H, 6.60; N, 8.34%; found: C, 78.59; H, 6.76; N, 8.25%.

*Spiro[2.3″]oxindole-spiro[3.3′]-5′-(2-methoxyphenylmethylidene)tetrahydro-4′(1H)-pyridinone-4-(2-methoxyphenyl)hexahydro-1H-pyrrolizine* (**6c**). Obtained as a brown solid, (0.140 g, 88%); mp = 187–188 °C; IR (KBr): 1603, 1619, 1702, 3389 cm^−1^; ^1^H-NMR (300 MHz, CDCl_3_): δ_H_ 1.69–2.00 (m, 4H, 5-CH_2 _and 6-CH_2_), 2.62 (d, 1H, *J =* 12.9 Hz, 2'-CH_2_), 2.83 (td, 1H, *J =* 8.1, 3.0 Hz, 7-CH_2_), 3.30–3.43 (m, 1H, 7-CH_2_), 3.65 (s, 3H, OCH_3_), 3.71–3.81 (m, 1H, 6'-CH_2_), 3.85 (s, 3H, OCH_3_), 3.99–4.15 (m, 2H, 6'-CH_2_ and 2'-CH_2_), 4.64 (d, 1H, *J =* 10.8 Hz, 4-CH), 4.82–4.87 (m, 1H, 4a-CH), 6.62–7.37 (m, 12H, Ar-H and arylmethylidene), 7.51 (d, 1H, *J =* 7.2 Hz, Ar-H), 8.02 (s, 1H, 1"-NH).^ 13^C-NMR (75 MHz, CDCl_3_): δ_C_ 25.31, 27.97, 48.65, 49.36, 50.51, 54.02, 54.97, 55.88, 65.45, 70.05, 74.18, 110.51, 111.11, 111.17, 120.49, 120.90, 122.44, 125.24, 126.17, 128.17, 128.33, 129.61, 130.69, 130.84, 131.03, 132.16, 135.25, 135.58, 142.01, 158.02, 158.79, 180.27, 201.51. Anal. calcd for C_33_H_33_N_3_O_4_: C, 74.00; H, 6.21; N, 7.84%; found: C, 74.21; H, 6.10; N, 7.98%.

*Spiro[2.3″]oxindole-spiro[3.3′]-5′-(2-chlorophenylmethylidene)tetrahydro-4′(1H)-pyridinone-4-(2-chlorophenyl)hexahydro-1H-pyrrolizine* (**6d**). Obtained as a pale yellow solid, (0.145 g, 92%); mp = 172–173 °C; IR (KBr): 1605, 1618, 1702, 3389 cm^−1^; ^1^H-NMR (300 MHz, CDCl_3_): δ_H_ 1.79–2.19 (m, 4H, 5-CH_2_ and 6-CH_2_), 2.39 (d, 1H, *J =* 12.9 Hz, 2'-CH_2_), 2.59–2.64 (m, 1H, 7-CH_2_), 3.22–3.28 (m, 1H, 7-CH_2_), 3.45–3.66 (m, 2H, 6'-CH_2_), 3.95 (d, 1H, *J =* 12.9 Hz, 2'-CH_2_), 4.55–4.60 (m, 1H, 4-CH), 4.82–4.87 (m, 1H, 4a-CH), 6.61–7.74 (m, 13H, Ar-H and arylmethylidene), 8.72 (s, 1H, 1"-NH). ^13^C-NMR (75 MHz, CDCl_3_): δ_C_ 25.81, 28.35, 48.10, 48.27, 48.81, 52.20, 64.19, 70.19, 74.78, 110.10, 122.62, 126.78, 126.86, 127.54, 128.74, 130.14, 130.54, 130.85, 131.06, 132.04, 132.92, 133.34, 133.60, 133.85, 135.47, 136.42, 136.66, 137.46, 141.69, 180.46, 201.23. Anal. calcd for C_31_H_27_Cl_2_N_3_O_2_: C, 68.38; H, 5.00; N, 7.72%; found: C, 68.23; H, 5.17; N, 7.61%.

*Spiro[2.3″]oxindole-spiro[3.3′]-5′-(2-bromophenylmethylidene)tetrahydro-4′(1H)-pyridinone-4-(2-bromophenyl)hexahydro-1H-pyrrolizine* (**6e**). Obtained as a light brown solid, (0.134 g, 92%); mp = 164–165 °C; IR (KBr): 1604, 1619, 1700, 3391 cm^−1^; ^1^H-NMR (300 MHz, CDCl_3_): δ_H_ 1.67–2.14 (m, 4H, 5-CH_2_ and 6-CH_2_), 2.24–2.28 (m, 1H, 2'-CH_2_), 2.52–2.65 (m, 1H, 7-CH_2_), 3.22–3.29 (m, 1H, 7-CH_2_), 3.46–3.61 (m, 2H, 6'-CH_2_), 3.92 (d, 1H, *J =* 12.9 Hz, 2'-CH_2_), 4.55–4.60 (m, 1H, 4-CH), 4.80–4.90 (m, 1H, 4a-CH), 6.60 (d, 1H, *J =* 7.2 Hz, Ar-H), 6.76–7.655 (m, 11H, Ar-H and arylmethylidene), 7.75 (d, 1H, *J =* 7.2 Hz, Ar-H), 8.99 (s, 1H, 1"-NH). ^13^C-NMR (75 MHz, CDCl_3_): δ_C_ 25.75, 28.46, 47.89, 48.09, 48.61, 51.55, 64.19, 70.91, 75.32, 110.23, 122.47, 125.66, 127.39, 127.50, 127.61, 129.09, 130.68, 130.87, 131.06, 133.32, 133.54, 134.05, 134.64, 135.58, 135.68, 135.93, 136.27, 137.16, 141.84, 180.50, 201.21. Anal. calcd for C_31_H_27_Br_2_N_3_O_2_: C, 58.79; H, 4.30; N, 6.63%; found: C, 58.93; H, 4.12; N, 6.75%.

*Spiro[2.3″]oxindole-spiro[3.3′]-5′-(2-fluorophenylmethylidene)-tetrahydro-4′(1H)-pyridinone-4-(2-fluorophenyl)hexahydro-1H-pyrrolizine* (**6f**). Obtained as a white solid; mp = 198–199 °C; IR (KBr): 1606, 1620, 1703, 3392 cm^−1^; ^1^H-NMR (500 MHz, CDCl_3_): δ_H_ 1.65–1.72 (m, 1H, 5-CH_2_), 1.82–1.88 (m, 1H, 6-CH_2_), 1.99–2.13 (m, 2H, 6-CH_2 _and 5-CH_2_), 2.35 (d, 1H, *J =* 13.0 Hz, 2'-CH_2_), 2.62 (td, 1H, *J =* 8.0, 2.0 Hz, 7-CH_2_), 3.31–3.36 (m, 1H, 7-CH_2_), 3.42–3.50 (m, 2H, 6'-CH_2_), 3.87 (d, 1H, *J =* 13.0 Hz, 2'-CH_2_), 4.64 (d, 1H, *J =* 10.5 Hz, 4-CH), 4.81–4.85 (m, 1H, 4a-CH), 6.72 (d, 1H, *J =* 7.5 Hz, Ar-H), 6.88 (t, 1H, *J =* 7.0 Hz, Ar-H), 6.96–7.04 (m, 4H, Ar-H), 7.12 (d, 1H, *J =* 8.0 Hz, Ar-H), 7.15 (d, 1H, *J =* 8.0 Hz, Ar-H), 7.18 (s, 1H, arylmethylidene), 7.20–7.25 (m, 3H, Ar-H), 7.29 (d, 1H, *J =* 8.0 Hz, Ar-H), 7.59 (t, 1H, *J =* 7.0 Hz, Ar-H), 8.74 (s, 1H, 1"-NH). ^13^C-NMR (125 MHz, CDCl_3_): δ_C_ 25.16, 28.59, 47.30, 47.87, 48.32, 49.88, 65.78, 69.35, 74.86, 109.50, 115.45 (^2^*J*_CF_ = 18.0 Hz), 115.59 (^2^*J*_CF_ = 17.0 Hz), 121.73, 123.10 (*J*_CF_ = 10.0 Hz), 123.58 (*J*_CF_ = 3.0 Hz), 123.96 (*J*_CF_ = 3.0 Hz), 124.87 (*J*_CF_ = 12.0 Hz), 126.08, 128.36 (123.10 (*J*_CF_ = 7.0 Hz), 128.95, 129.22, 129.65 (*J*_CF_ = 3.0 Hz), 130.36 (*J*_CF_ =2.0 Hz), 130.48 (*J*_CF_ =8.0 Hz), 130.76 (*J*_CF_ = 4.0 Hz), 136.29, 141.62, 160.60 (^1^*J*_CF_ = 200.0 Hz), 161.86 (^1^*J*_CF_ = 196.0 Hz), 179.57, 199.06. Anal. calcd for C_31_H_27_F_2_N_3_O_2_: C, 72.78; H, 5.32; N, 8.21%; found: C, 72.96; H, 5.51; N, 8.09%.

*Spiro[2.3″]oxindole-spiro[3.3′]-5′-(2,4-chlorophenylmethylidene)tetrahydro-4′(1H)-pyridinone-4-(2,4-chlorophenyl)hexahydro-1H-pyrrolizine* (**6g**). Obtained as a pale yellow solid, (0.141 g, 95%); mp = 210–211 °C; IR (KBr): 1604, 1620, 1702, 3391 cm^−1^; ^1^H-NMR (300 MHz, CDCl_3_): δ_H_ 1.73–2.29 (m, 4H, 5-CH_2_ and 6-CH_2_), 2.60 (d, 1H, *J =* 12.3 Hz, 2'-CH_2_), 2.64–2.79 (m, 1H, 7-CH_2_), 3.21–3.30 (m, 1H, 7-CH_2_), 3.52–3.68 (m, 1H, 6'-CH_2_), 3.97 (d, 1H, *J =* 15.6 Hz, 6'-CH_2_), 4.14 (d, 1H, *J =* 12.3 Hz, 2'-CH_2_), 4.54–4.60 (m, 1H, 4-CH), 4.83–4.89 (m, 1H, 4a-CH), 6.62–7.92 (m, 11H, Ar-H and arylmethylidene), 8.75 (s, 1H, 1"-NH). ^13^C-NMR (75 MHz, CDCl_3_): δ_C_ 25.82, 28.51, 48.14, 48.54, 49.25, 52.23, 65.17, 70.21, 74.73, 110.29, 122.77, 127.23, 127.31, 128.37, 130.10, 130.30, 130.73, 130.87, 131.48, 131.68, 132.26, 132.59, 133.93, 135.49, 135.90, 136.34, 137.15, 141.75, 180.37, 201.14. Anal. calcd for C_31_H_25_Cl_4_N_3_O_2_: C, 60.70; H, 4.11; N, 6.85%; found: C, 60.87; H, 4.23; N, 6.64%.

*Spiro[2.3″]oxindole-spiro[3.3′]-5′-(3-nitrophenylmethylidene)tetrahydro-4′(1H)-pyridinone-4-(3-nitrophenyl)hexahydro-1H-pyrrolizine* (**6h**). Obtained as a pale yellow solid, (0.144 g, 93%); mp = 204–205 °C; IR (KBr): 1608, 1621, 1707, 3386 cm^−1^; ^1^H-NMR (300 MHz, CDCl_3_): δ_H_ 1.58–2.02 (m, 4H, 5-CH_2_ and 6-CH_2_), 2.27 (d, 1H, *J =* 12.6 Hz, 2'-CH_2_), 2.60–2.63 (m, 1H, 7-CH_2_), 3.06–3.15 (m, 1H, 7-CH_2_), 3.46–3.51 (m, 1H, 6'-CH_2_), 3.62 (d, 1H, *J =* 14.7 Hz, 6'-CH_2_), 4.01 (d, 1H, *J =* 12.6 Hz, 2'-CH_2_), 4.48 (d, 1H, *J =* 10.8 Hz, 4-CH), 4.65–4.73 (m, 1H, 4a-CH), 6.70–8.25 (m, 13H, Ar-H and arylmethylidene), 9.10 (s, 1H, 1"-NH). ^13^C-NMR (75 MHz, CDCl_3_): δ_C_ 26.10, 29.10, 48.13, 48.86, 49.07, 51.06, 63.20, 71.89, 74.04, 110.72, 122.78, 123.02, 123.99, 124.68, 125.03, 125.41, 125.90, 127.47, 129.68, 130.04, 133.91, 135.68, 136.30, 136.93, 137.33, 137.90, 141.92, 148.45, 148.61, 180.54, 201.06. Anal. calcd for C_31_H_27_N_5_O_6_: C, 65.83; H, 4.81; N, 12.38%; found: C, 65.69; H, 4.94; N, 12.47%.

*Spiro[2.3″]oxindole-spiro[3.3′]-5′-(4-methylphenylmethylidene)tetrahydro-4′(1H)-pyridinone-4-(4-methylphenyl)hexahydro-1H-pyrrolizine* (**6i**). Obtained as a brown solid, (0.156 g, 94%); mp = 168–169 °C; IR (KBr): 1611, 1623, 1704, 3392 cm^−1^; ^1^H-NMR (300 MHz, CDCl_3_): δ_H_ 1.60–2.03 (m, 4H, 5-CH_2_ and 6-CH_2_), 2.29 (d, 1H, *J =* 12.9 Hz, 2'-CH_2_), 2.36 (s, 3H, CH_3_), 2.38 (s, 3H, CH_3_), 2.58–2.63 (m, 1H, 7-CH_2_), 3.10–3.16 (m, 1H, 7-CH_2_), 3.49 (d, 1H, *J =* 15.3 Hz, 6'-CH_2_), 3.78 (d, 1H, *J =* 15.3 Hz, 6'-CH_2_), 3.99–4.03 (m, 1H, 2'-CH_2_), 4.36 (d, 1H, *J =* 11.7 Hz, 4-CH), 4.50–4.55 (m, 1H, 4a-CH), 6.66 (d, 1H, *J =* 7.5 Hz, Ar-H), 6.84–7.54 (m, 12H, Ar-H and arylmethylidene), 8.56 (s, 1H, 1"-NH). ^13^C-NMR (75 MHz, CDCl_3_): δ_C_ 21.3, 21.8, 26.07, 28.83, 48.50, 48.78, 49.35, 53.42, 65.96, 71.84, 74.06, 110.25, 122.61, 126.02, 129.33, 129.66, 129.74, 130.51, 131.01, 131.04, 132.11, 132.78, 135.12, 136.49, 136.97, 139.82, 141.96, 181.00, 202.06. Anal. calcd for C_33_H_33_N_3_O_2_: C, 78.70; H, 6.60; N, 8.34%; found: C, 78.84; H, 6.42; N, 8.42%.

*Spiro[2.3″]oxindole-spiro[3.3′]-5′-(4-methoxyphenylmethylidene)tetrahydro-4′(1H)-pyridinone-4-(4-methoxyphenyl)hexahydro-1H-pyrrolizine* (**6j**). Obtained as a brown solid, (0.135 g, 85%); mp = 204–205 °C; IR (KBr): 1610, 1623, 1703, 3394 cm^−1^; ^1^H-NMR (300 MHz, CDCl_3_): δ_H_ 1.58–2.05 (m, 4H, 5-CH_2_ and 6-CH_2_), 2.32 (d, 1H, *J =* 12.6 Hz, 2'-CH_2_), 2.57–2.65 (m, 1H, 7-CH_2_), 3.08–3.15 (m, 1H, 7-CH_2_), 3.48 (d, 1H, *J =* 15.3 Hz, 6'-CH_2_), 3.62 (s, 3H, OCH_3_), 3.69 (s, 3H, OCH_3_), 3.82 (d, 1H, *J =* 15.3 Hz, 6'-CH_2_), 3.97–4.06 (m, 1H, 2'-CH_2_), 4.38 (d, 1H, *J =* 11.4 Hz, 4-CH), 4.52–4.56 (m, 1H, 4a-CH), 6.65–7.54 (m, 13H, Ar-H and arylmethylidene), 8.52 (s, 1H, 1"-NH). ^13^C-NMR (75 MHz, CDCl_3_): δ_C_ 26.12, 28.86, 47.50, 48.18, 49.63, 53.13, 55.20, 55.31, 64.41, 70.34, 74.39, 110.12, 113.45, 113.92, 122.64, 127.42, 127.86, 129.51, 130.57, 132.21, 132.98, 137.44, 141.53, 158.33, 159.85, 181.03, 201.06. Anal. calcd for C_33_H_33_N_3_O_4_: C, 74.00; H, 6.21; N, 7.84%; found: C, 74.26; H, 6.14; N, 7.76%.

*Spiro[2.3″]oxindole-spiro[3.3′]-5′-(4-chlorophenylmethylidene)tetrahydro-4′(1H)-pyridinone-4-(4-chlorophenyl)hexahydro-1H-pyrrolizine* (**6k**). Obtained as a pale yellow solid, (0.150 g, 95%); mp = 175–176 °C; IR (KBr): 1610, 1621, 1703, 3387 cm^−1^; ^1^H-NMR (300 MHz, CDCl_3_): δ_H_ 1.56–2.04 (m, 4H, 5-CH_2_ and 6-CH_2_), 2.29 (d, 1H, *J =* 12.9 Hz, 2'-CH_2_), 2.58–2.62 (m, 1H, 7-CH_2_), 3.07–3.15 (m, 1H, 7-CH_2_), 3.49 (d, 1H, *J =* 15.6 Hz, 6'-CH_2_), 3.76 (dd, 1H, *J =* 15.6, 2.1 Hz, 6'-CH_2_), 4.05–4.16 (m, 1H, 2'-CH_2_), 4.35 (d, 1H, *J =* 11.4 Hz, 4-CH), 4.51–4.56 (m, 1H, 4a-CH), 6.68 (d, 1H, *J =* 7.8 Hz, Ar-H), 6.88–7.55 (m, 12H, Ar-H and arylmethylidene), 8.66 (s, 1H, 1"-NH). ^13^C-NMR (75 MHz, CDCl_3_): δ_C_ 26.08, 28.96, 48.29, 48.85, 49.20, 53.41, 63.99, 71.86, 74.04, 110.43, 122.84, 125.96, 128.03, 128.82, 129.21, 129.29, 131.73, 132.07, 132.11, 133.63, 133.88, 135.37, 135.49, 136.05, 141.85, 180.74, 201.55. Anal. calcd for C_31_H_27_Cl_2_N_3_O_2_: C, 68.38; H, 5.00; N, 7.72%; found: C, 68.59; H, 5.15; N, 7.60%.

*Spiro[2.3″]oxindole-spiro[3.3′]-5′-(4-bromophenylmethylidene)tetrahydro-4′(1H)-pyridinone-4-(4-bromophenyl)hexahydro-1H-pyrrolizine* (**6l**). Obtained as a pale yellow solid, (0.136 g, 93%); mp = 207–208 °C; IR (KBr): 1612, 1619, 1702, 3389 cm^−1^; ^1^H-NMR (300 MHz, CDCl_3_): δ_H_ 1.52–2.07 (m, 4H, 5-CH_2_ and 6-CH_2_), 2.29 (d, 1H, *J =* 13.2 Hz, 2'-CH_2_), 2.58–2.62 (m, 1H, 7-CH_2_), 3.06–3.14 (m, 1H, 7-CH_2_), 3.47 (d, 1H, *J =* 15.6 Hz, 6'-CH_2_), 3.75 (dd, 1H, *J =* 15.6, 2.1 Hz, 6'-CH_2_), 4.05–4.15 (m, 1H, 2'-CH_2_), 4.33 (d, 1H, *J =* 11.1 Hz, 4-CH), 4.51–4.56 (m, 1H, 4a-CH), 6.69 (d, 1H, *J =* 7.8 Hz, Ar-H), 6.81–7.55 (m, 12H, Ar-H and arylmethylidene), 8.60 (s, 1H, 1"-NH). ^13^C-NMR (75 MHz, CDCl_3_): δ_C_ 26.10, 28.98, 48.35, 48.76, 49.25, 53.28, 64.98, 71.85, 74.12, 110.41, 121.80, 122.84, 123.92, 128.06, 129.83, 131.64, 131.77, 131.92, 132.17, 132.25, 132.39, 134.32, 135.44, 136.23, 141.81, 180.70, 201.52. Anal. calcd for C_31_H_27_Br_2_N_3_O_2_: C, 58.79; H, 4.30; N, 6.63%; found: C, 58.96; H, 4.47; N, 6.51%.

*Spiro[2.3″]oxindole-spiro[3.3′]-5′-(4-fluorophenylmethylidene)tetrahydro-4′(1H)-pyridinone-4-(4-fluorophenyl)hexahydro-1H-pyrrolizine* (**6m**). Obtained as a pale yellow solid, (0.154 g, 94%); mp = 192–193 °C; IR (KBr): 1611, 1620, 1701, 3390 cm^−1^; ^1^H-NMR (500 MHz, CDCl_3_): δ_H_ 1.50–2.03 (m, 4H, 5-CH_2_ and 6-CH_2_), 2.30 (d, 1H, *J =* 13.5 Hz, 2'-CH_2_), 2.57–2.65 (m, 1H, 7-CH_2_), 3.03–3.12 (m, 1H, 7-CH_2_), 3.37 (d, 1H, *J =* 15.0 Hz, 6'-CH_2_), 3.77 (d, 1H, *J =* 15.0, 2.1 Hz, 6'-CH_2_), 4.06–4.13 (m, 1H, 2'-CH_2_), 4.36 (d, 1H, *J =* 11.0 Hz, 4-CH), 4.52–4.55 (m, 1H, 4a-CH), 6.61–7.54 (m, 13H, Ar-H and arylmethylidene), 8.62 (s, 1H, 1"-NH). ^13^C-NMR (125 MHz, CDCl_3_): δ_C_ 25.67, 28.54, 47.84, 48.47, 48.70, 52.83, 64.51, 69.60, 74.93, 109.99, 115.12 (^2^*J*_CF_ = 20.0 Hz), 115.80 (^2^*J*_CF_ = 21.25 Hz), 122.41, 125.59, 127.73, 128.70, 130.40, 131.71, 132.13, 132.52, 133.61, 135.17, 141.52, 161.95 (^1^*J*_CF_ = 245.0 Hz), 163.01 (^1^*J*_CF_ =250.0 Hz), 180.62, 201.19. Anal. calcd for C_31_H_27_F_2_N_3_O_2_: C, 72.78; H, 5.32; N, 8.21%; found: C, 72.92; H, 5.54; N, 8.04%.

*Spiro[2.3″]oxindole-spiro[3.3′]-5′-(1-naphthylmethylidene)tetrahydro-4′(1H)-pyridinone-4-(1-naphthyl)hexahydro-1H-pyrrolizine* (**6n**). Obtained as a yellow solid, (0.139 g, 91%); mp = 158–159 °C; IR (KBr): 1607, 1619, 1705, 3391 cm^−1^; ^1^H-NMR (300 MHz, CDCl_3_): δ_H_ 1.82–2.09 (m, 4H, 5-CH_2_ and 6-CH_2_), 2.65–2.68 (m, 1H, 7-CH_2_), 2.78 (d, 1H, *J =* 12.9 Hz, 2'-CH_2_), 3.30–3.35 (m, 1H, 7-CH_2_), 3.44 (d, 1H, *J =* 15.6 Hz, 6'-CH_2_), 3.87 (d, 1H, *J =* 15.6 Hz, 6'-CH_2_), 4.22 (d, 1H, *J =* 12.6 Hz, 2'-CH_2_), 4.44–4.50 (m, 1H, 4-CH), 4.68–4.73 (m, 1H, 4a-CH), 6.27 (s, 1H, arylmethylidene), 6.41–8.07 (m, 18H, Ar-H), 9.02 (s, 1H, 1"-NH). ^13^C-NMR (75 MHz, CDCl_3_): δ_C_ 25.99, 28.74, 48.00, 48.68, 49.30, 51.14, 64.58, 70.98, 75.04, 110.30, 122.63, 124.62, 125.05, 125.30, 125.45, 125.75, 126.77, 127.16, 127.59, 127.91, 128.20, 128.85, 129.08, 129.22, 129.39, 129.64, 130.05, 132.45, 132.48, 133.64, 133.70, 133.96, 134.46, 134.90, 136.90, 137.73, 138.82, 141.09, 181.13, 202.63. Anal. calcd for C_39_H_33_N_3_O_2_: C, 81.37; H, 5.78; N, 7.30%; found: C, 81.59; H, 5.60; N, 7.43%.

### 3.5. Cell Culture and Cell Proliferation Assay

#### 3.5.1. Cell Culture

Human ovarian adenocarcinoma cell line (SK-OV-3, ATCC no. HTB-77), human breast carcinoma (MDA-MB-231, ATCC no. HTB-26), and human lymphoblastic leukemia cell line (CCRF-CEM, ATCC no. CCL-119) obtained from American Type Culture Collection. The cells were grown on 75 cm^2^ cell culture flasks with RPMI-16 medium for CCRF-CEM cells and EMEM (Eagle’s minimum essential medium) for SK-OV-3 and MDA-MB-231 cells, and supplemented with 10% fetal bovine serum, and 1% penicillin/streptomycin solution (10,000 units of penicillin and 10 mg of streptomycin in 0.9% NaCl) in a humidified atmosphere of 5% CO_2_, 95% air at 37 °C.

#### 3.5.2. Cell Proliferation Assay

The cell proliferation assay was carried out using CellTiter 96 aqueous one solution cell proliferation assay kit (Promega, Madison, WI, USA). Briefly, upon reaching about 75%–80% confluency, SK-OV-3 (5,000 cells/well), MDA-MB-231 (5,000 cells/well), or CCRF-CEM (40,000 cells/well) were plated in 96-well microplate in 100 µL media. After seeding for 24 h, the cells were treated with 50 µM compound in triplicate. Doxorubicin (10 µM) was used as the positive control. At the end of the sample exposure period (72 h), CellTiter 96 aqueous solution (20 µL) was added. The plate was returned to the incubator for 1 h in a humidified atmosphere at 37 °C. The absorbance of the formazan product was measured at 490 nm using a microplate reader. The blank control was recorded by measuring the absorbance at 490 nm with wells containing medium mixed with CellTiter 96 aqueous solution but no cells. Results were expressed as the percentage of the control (without compound set at 100%). The percentage of cell survival was calculated as [OD value of cells treated with the test compound − OD value of culture medium]/[(OD value of control cells − OD value of culture medium)] × 100%.

#### 3.5.3. IC_50_ Determination Assay

IC_50_ determination assay was performed by CellTiter 96 aqueous one solution cell proliferation assay kit (Promega). Briefly, SK-OV-3 (5,000 cells/well) and CCRF-CEM (40,000 cells/well) were seeded in 96-well plate in media (100 µL). After 24 h, the cells were treated with various concentrations of compounds (1–100 µM) in triplicate. After 72 h of incubation, 20 µL CellTiter 96 aqueous solution was added to wells. The plate was kept in the incubator for 1 h in a humidified atmosphere at 37 °C. The absorbance of the formazan product was measured at 490 nm using microplate reader. The blank control was recorded by measuring the absorbance at 490 nm with wells containing medium mixed with CellTiter 96 aqueous solution but no cells. The IC_50_ values were extrapolated from concentration–effect curves using non-linear regression analysis in GraphPad Prism^®^, version 5.03.

## 4. Conclusions

In conclusion, azomethine ylides generated *via* an *in situ* reaction between 1*H*-indole-2,3-dione with *N*-methylglycine, phenylglycine, or proline underwent [3+2]-cycloaddition with 3,5-bis[(*E*)-arylmethylidene]tetrahydro-4(1*H*)-pyridinones to afford different classes of spiropyrrolidines/pyrrolizines. A number of compounds exhibited antiproliferative activity against MDA-MB-231, CCRF-CEM, and SK-OV-3 cells. In general, most of the spiro-pyrrolizines derivatives showed higher antiproliferative activity when compared with *N*-methyl spiro-pyrrolidines and *N*-α-phenyl substituted spiro-pyrrolidines derivatives. Compounds **6a**, **6b**, and **6m** were found to be the most potent derivatives showing 64%, 87%, and 74% antiproliferative activity in MDA-MB-231, SK-OV-3, and CCRF-CEM cells, respectively. These lead compounds will be studied for their stereochemistry properties and have the potential as antiproliferative agents after further optimization.

## Supplementary Materials

Supplementary materials can be accessed at: http://www.mdpi.com/1420-3049/19/7/10033/s1.
