# Supplementary material for: Facile, Regio- and Diastereoselective Synthesis of Spiro-Pyrrolidine and Pyrrolizine Derivatives and Evaluation of Their Antiproliferative Activities
Source: Molecules. 2014 Jul 10;19(7):10033–55. doi: 10.3390/molecules190710033 (PMC6271256; doi:10.3390/molecules190710033)
Supplement: Supplementary file 1 [file molecules-19-10033-s001.pdf]

Supplementary Materials

Figure S1. ^{1}H-NMR spectrum of **4k**.

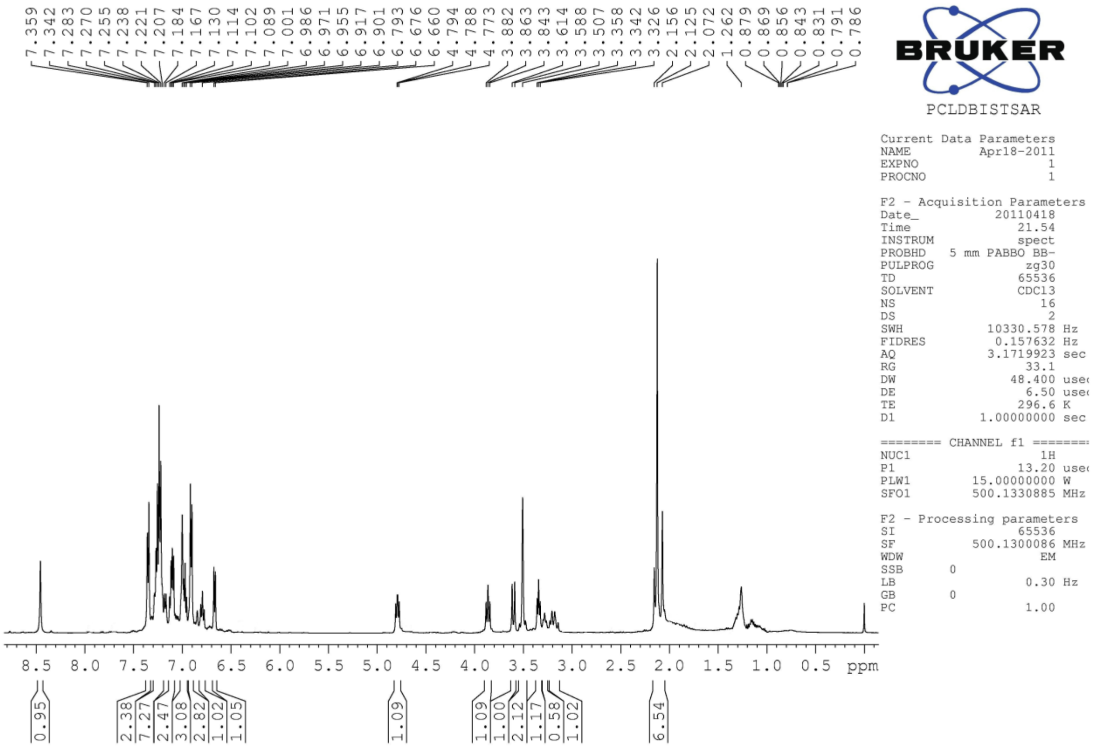

Figure S2. ^{1}H-NMR spectrum of **4k**.

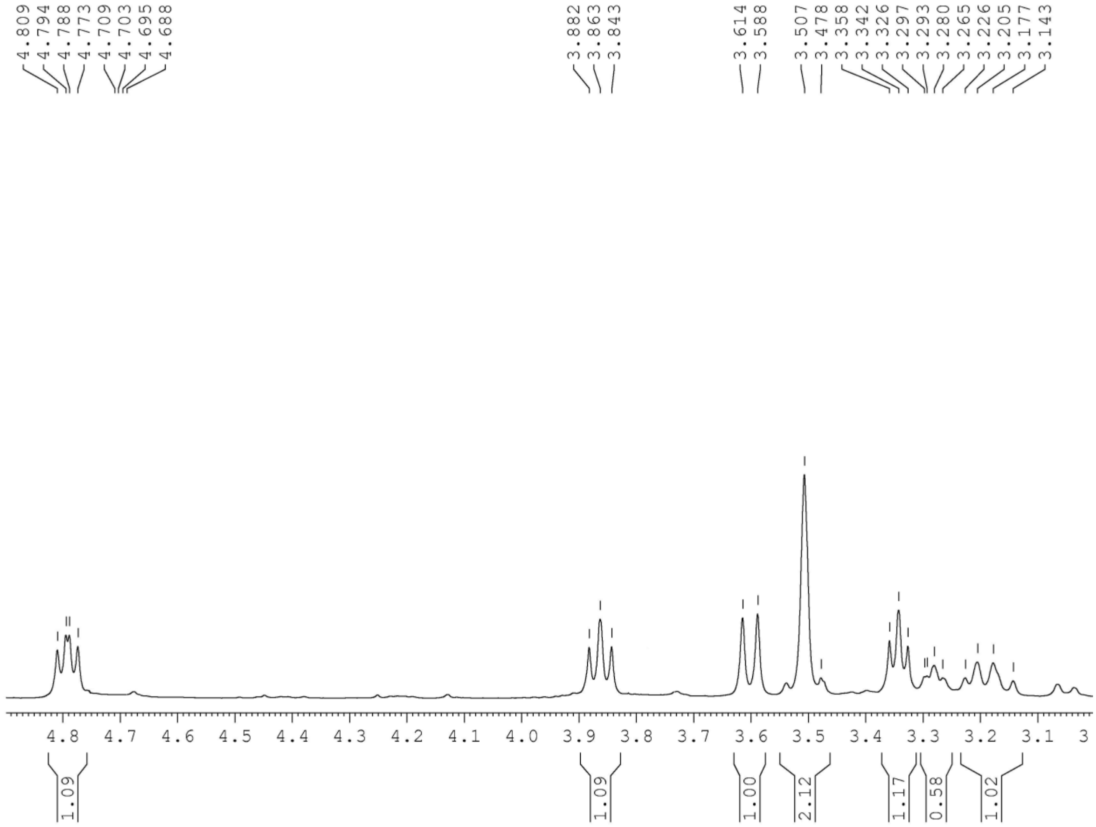

Figure S3. ^{13}C-NMR spectrum of **4k**.

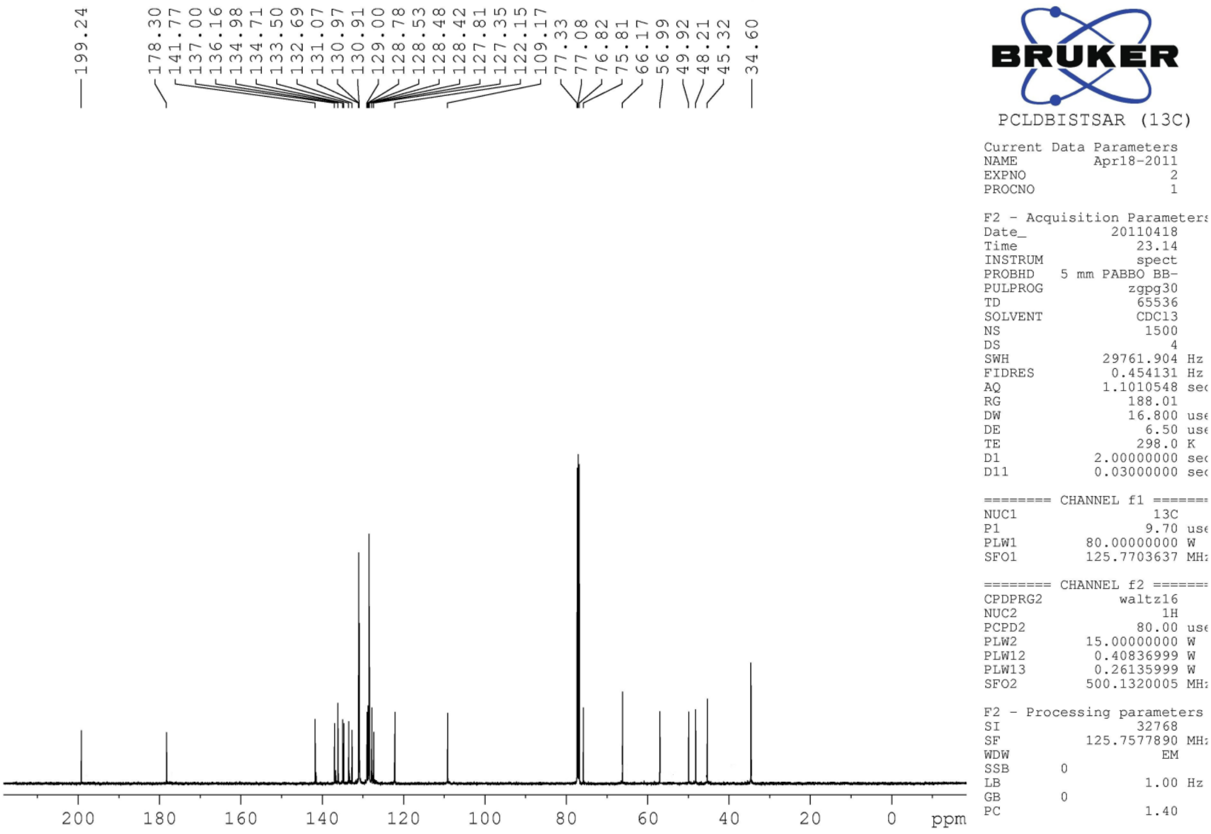

Figure S4. ^{13}C-NMR spectrum of **4k**.

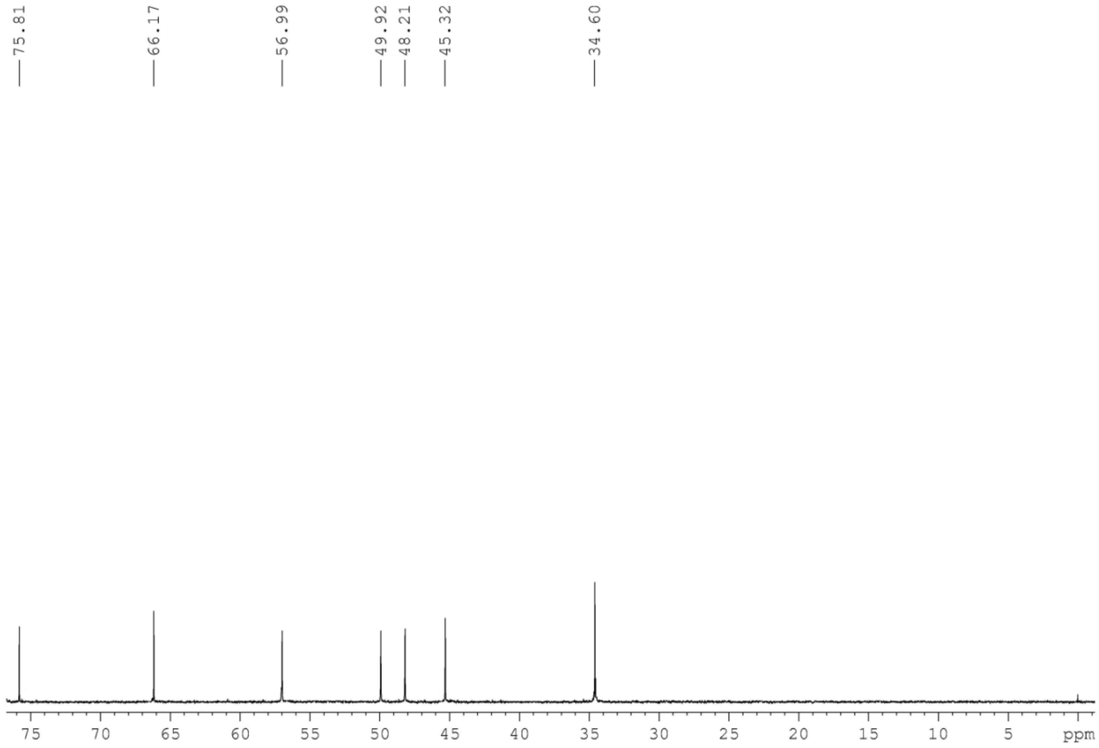

Figure S5. ^{13}C-NMR spectrum of **4k**.

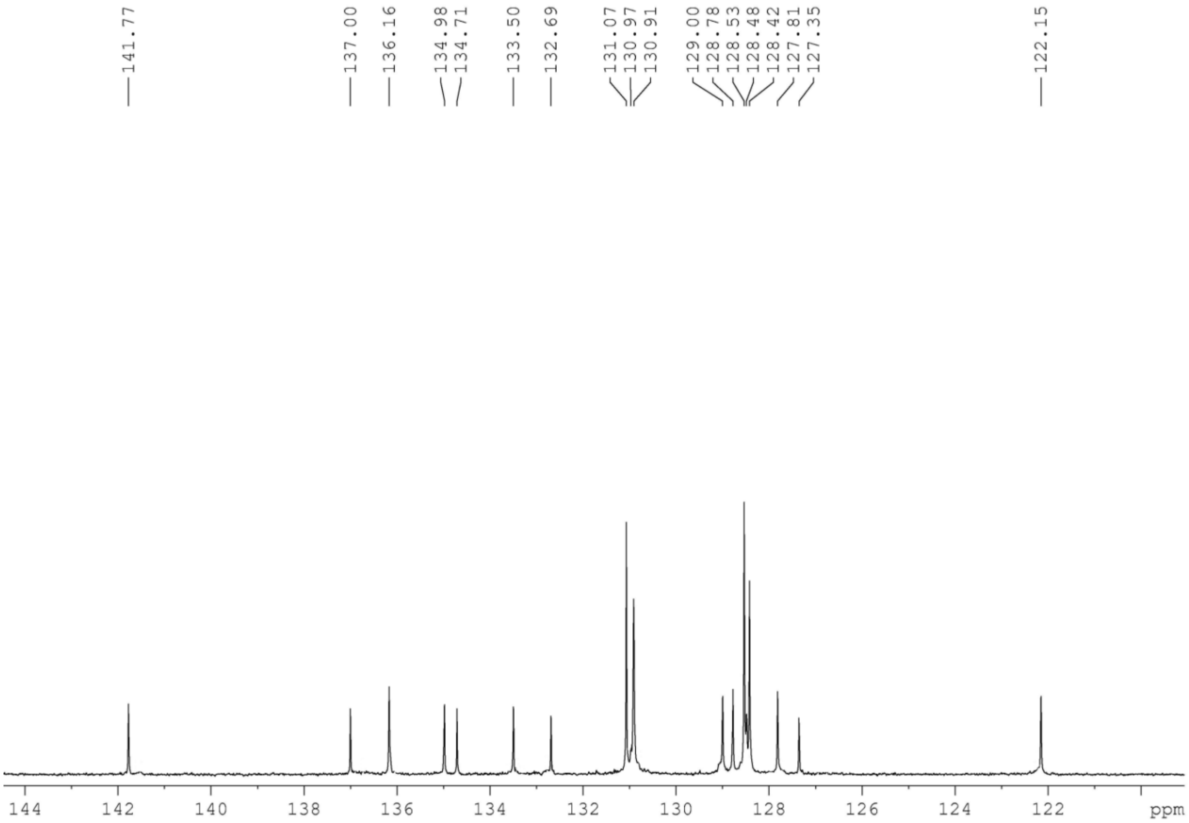

Figure S6. DEPT-135 NMR spectrum of **4k**.

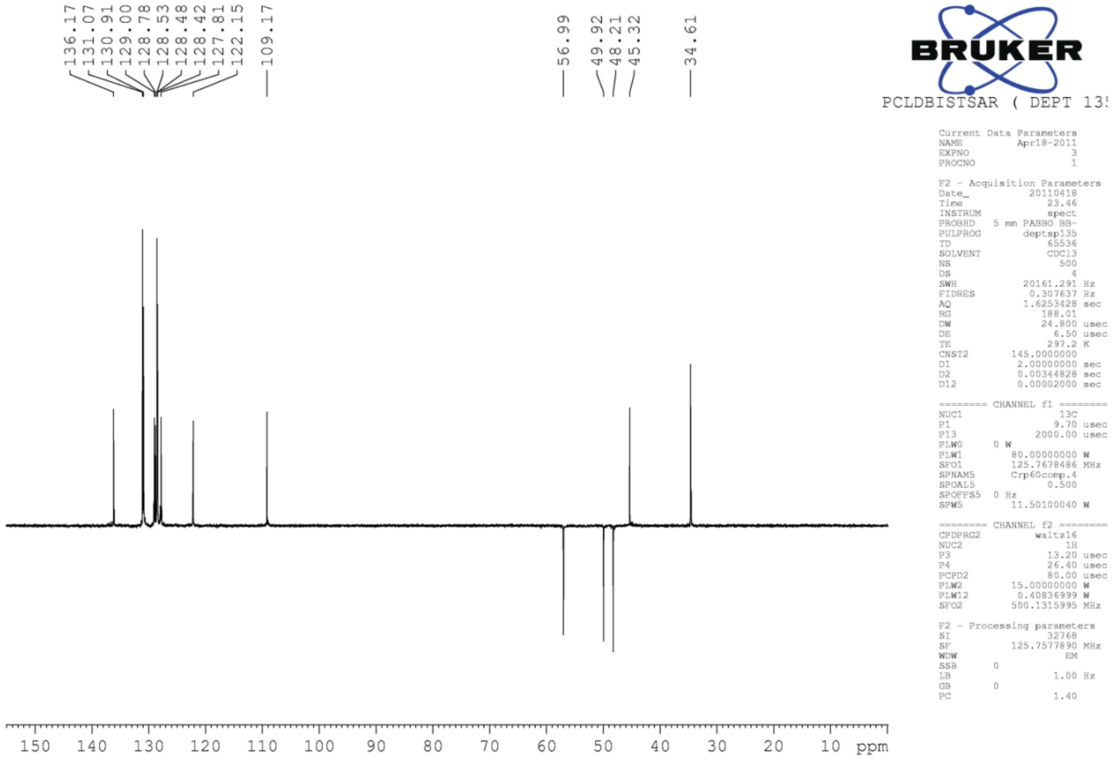

Figure S7. H,H-COSY spectrum of **4k**.

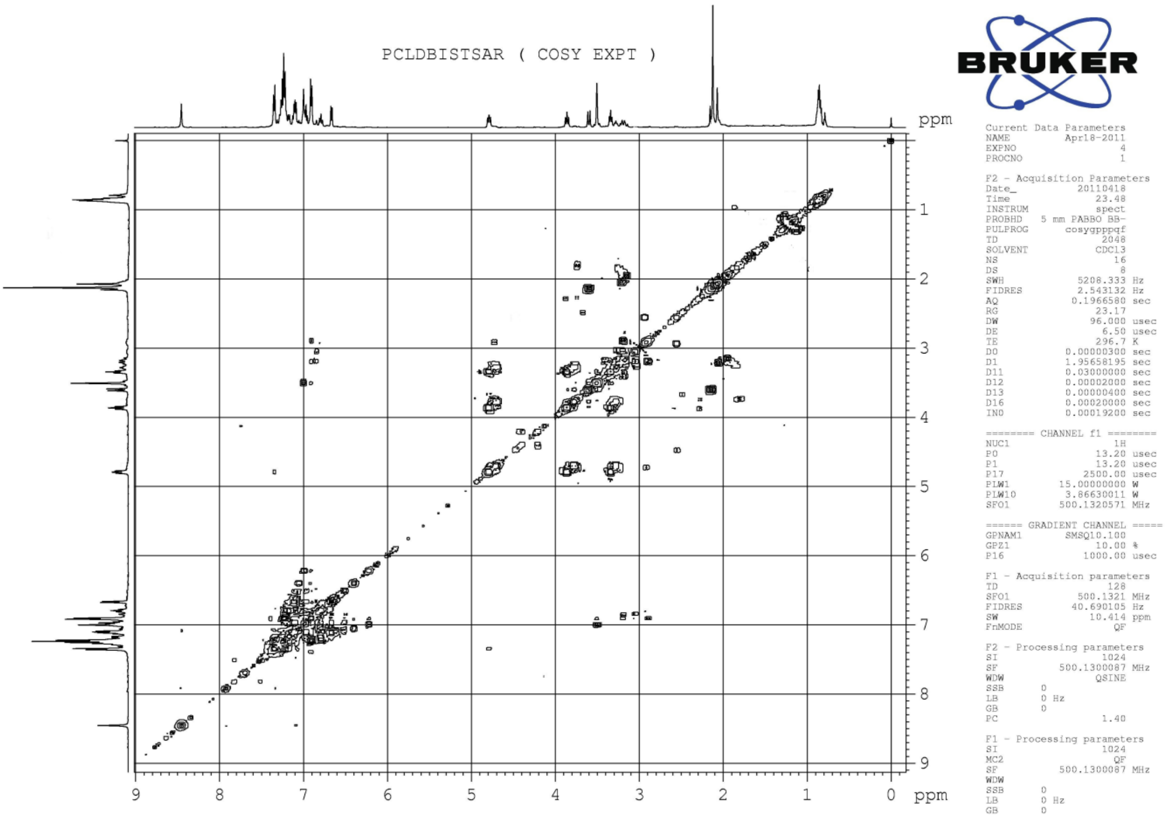

Figure S8. H,H-COSY spectrum of **4k**.

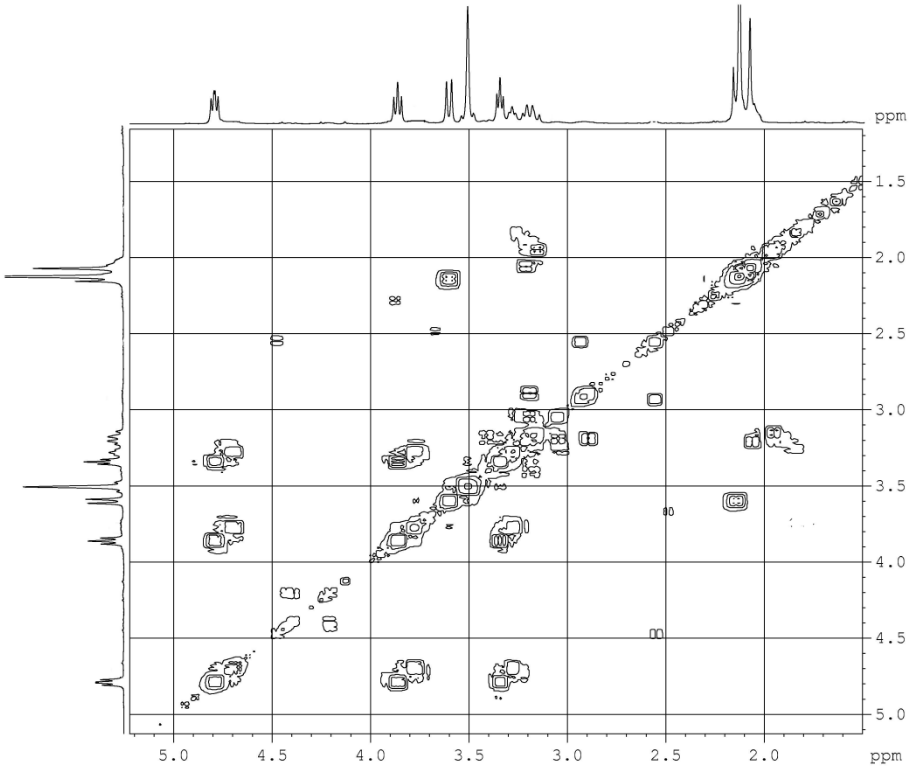

Figure S9. C,H-COSY spectrum of **4k**.

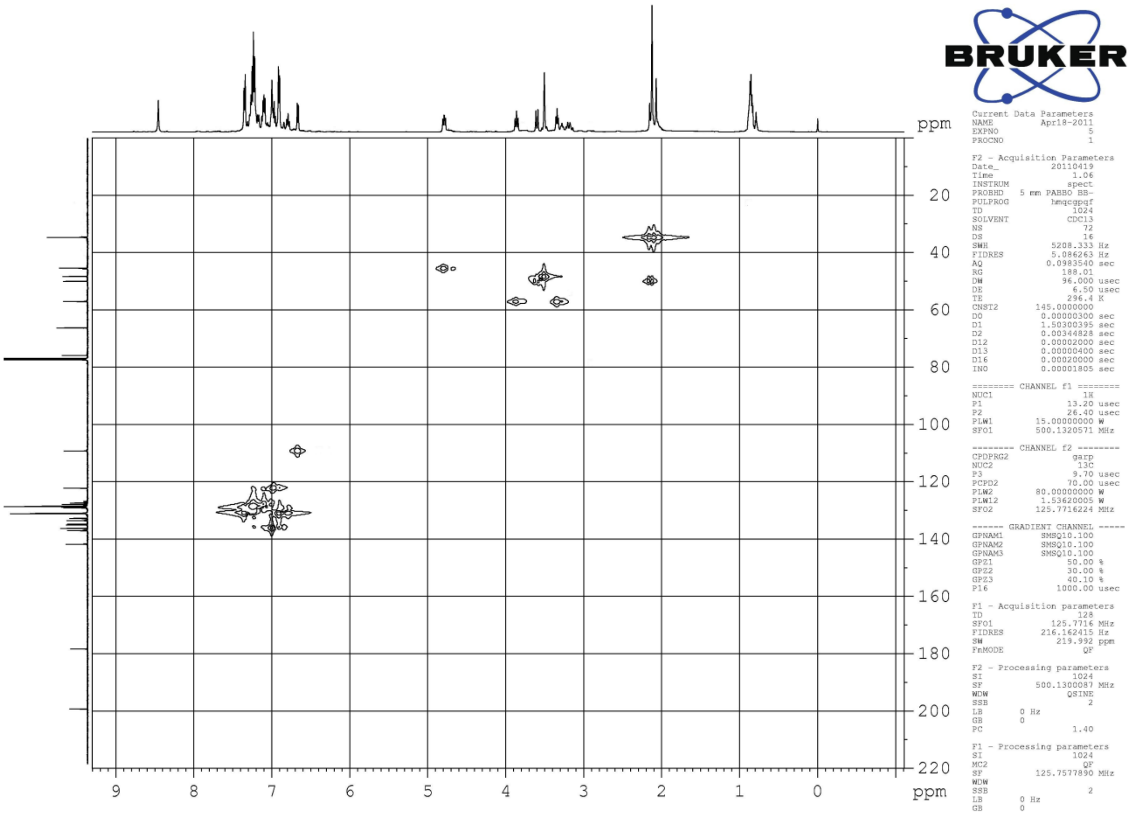

Figure S10. C,H-COSY spectrum of **4k**.

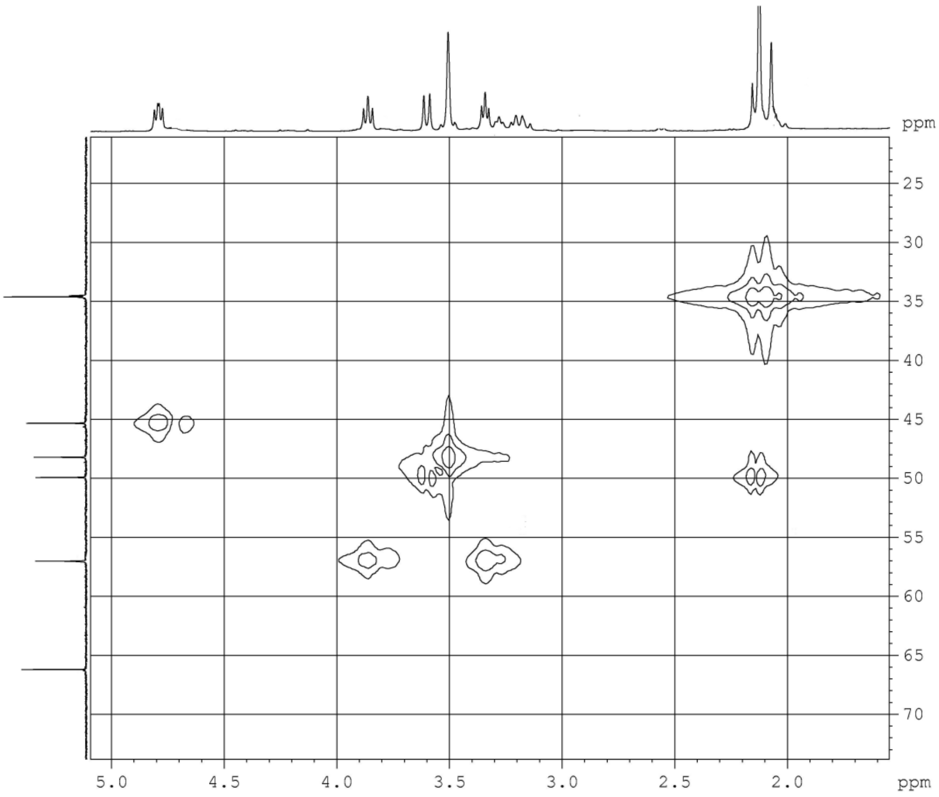

Figure S11. HMBC spectrum of **4k**.

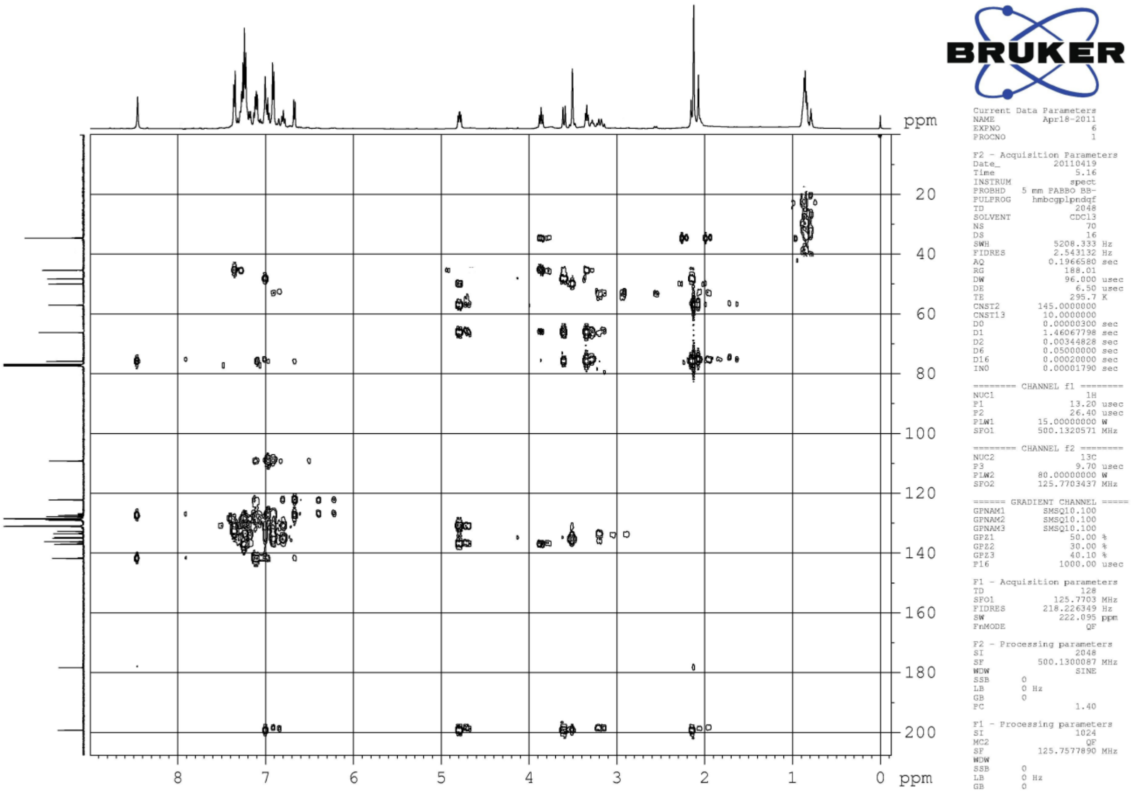

Figure S12. HMBC spectrum of **4k**.

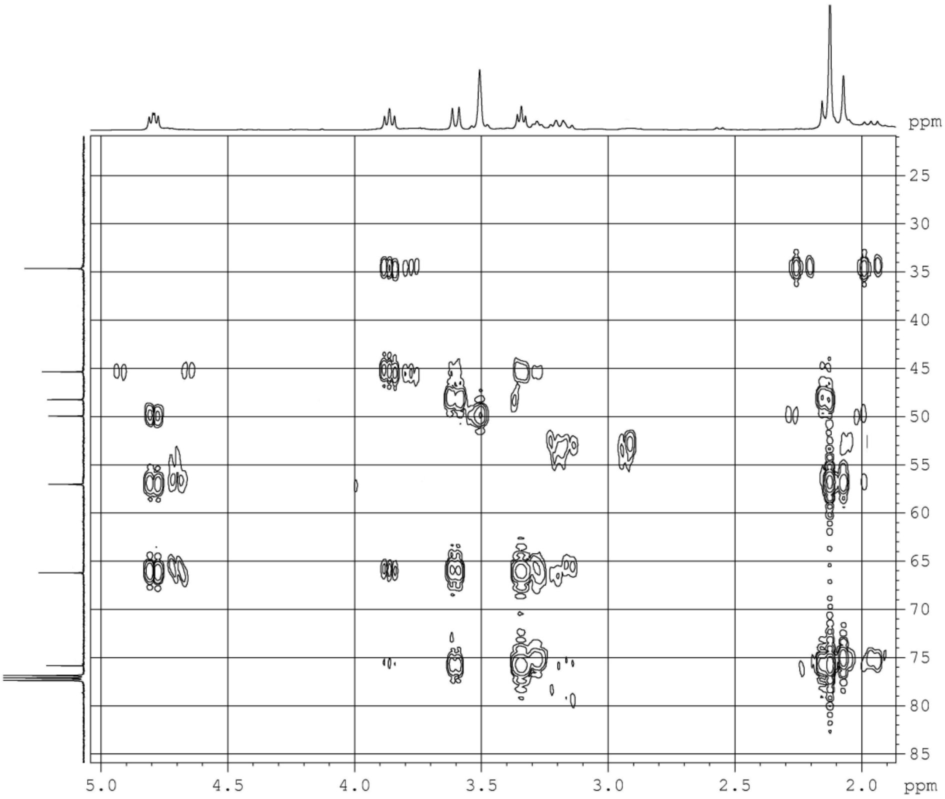

Figure S13. HMBC spectrum of **4k**.

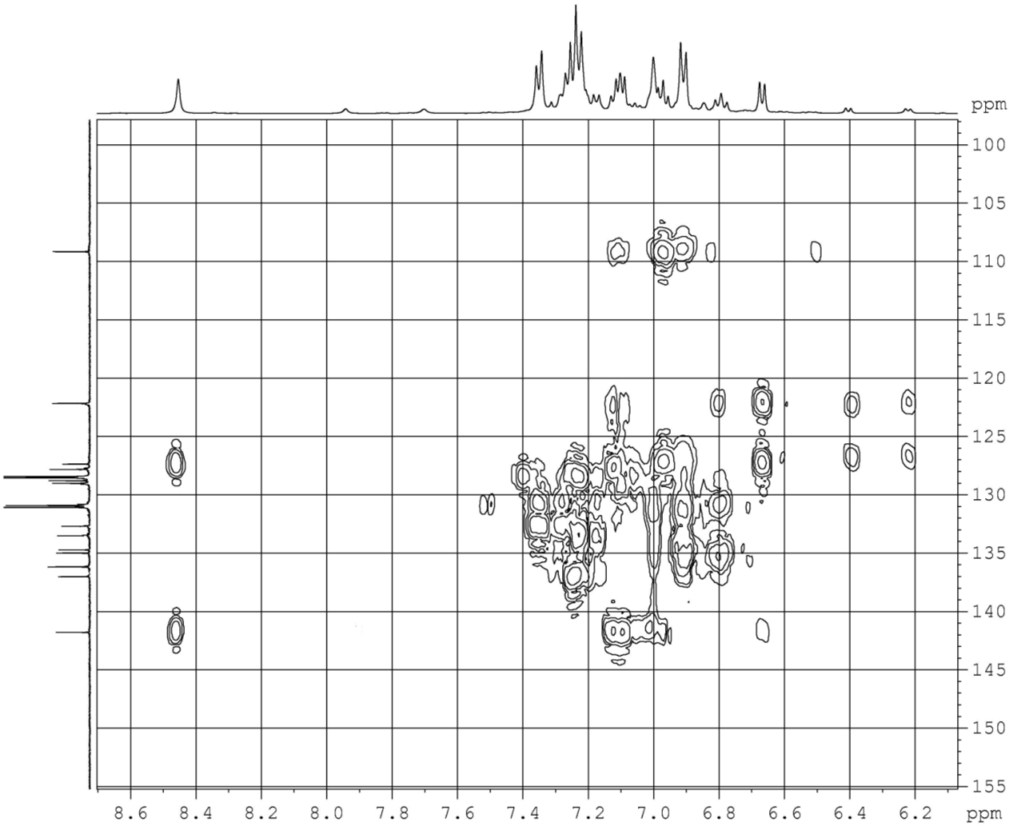

Figure S14. ^{1}H-NMR spectrum of **5a**.

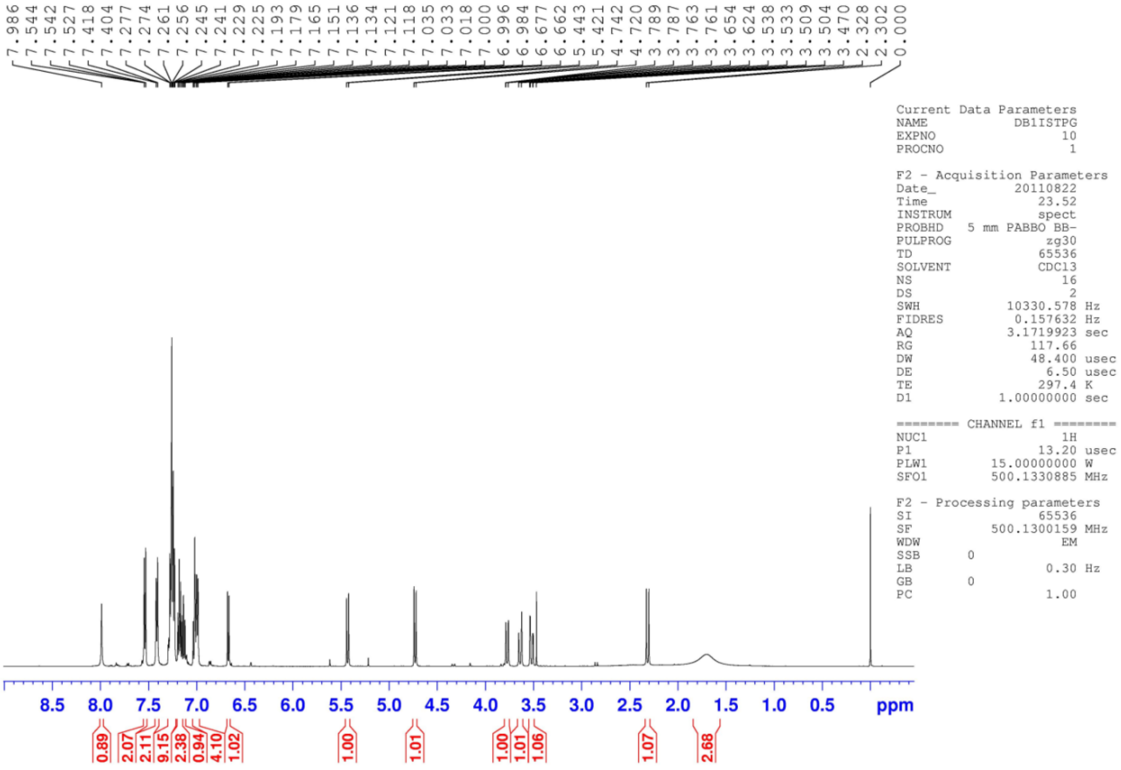

Figure S15. ^{1}H-NMR spectrum of **5a**.

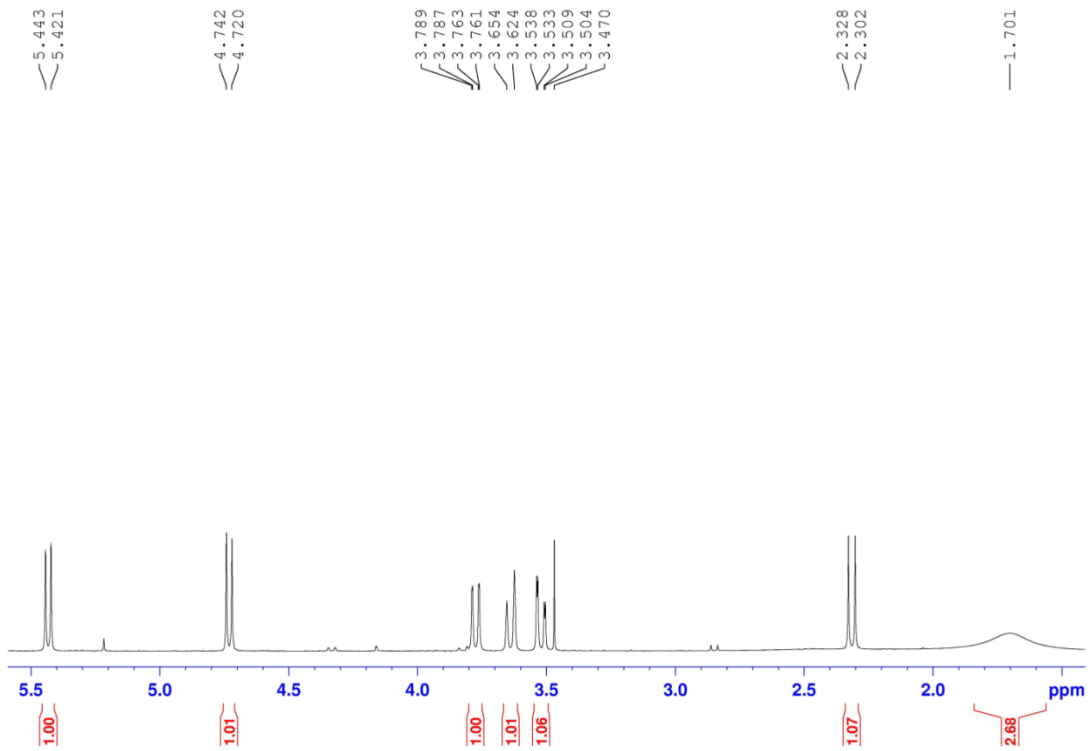

Figure S16. ^{1}H-NMR spectrum of **5a**.

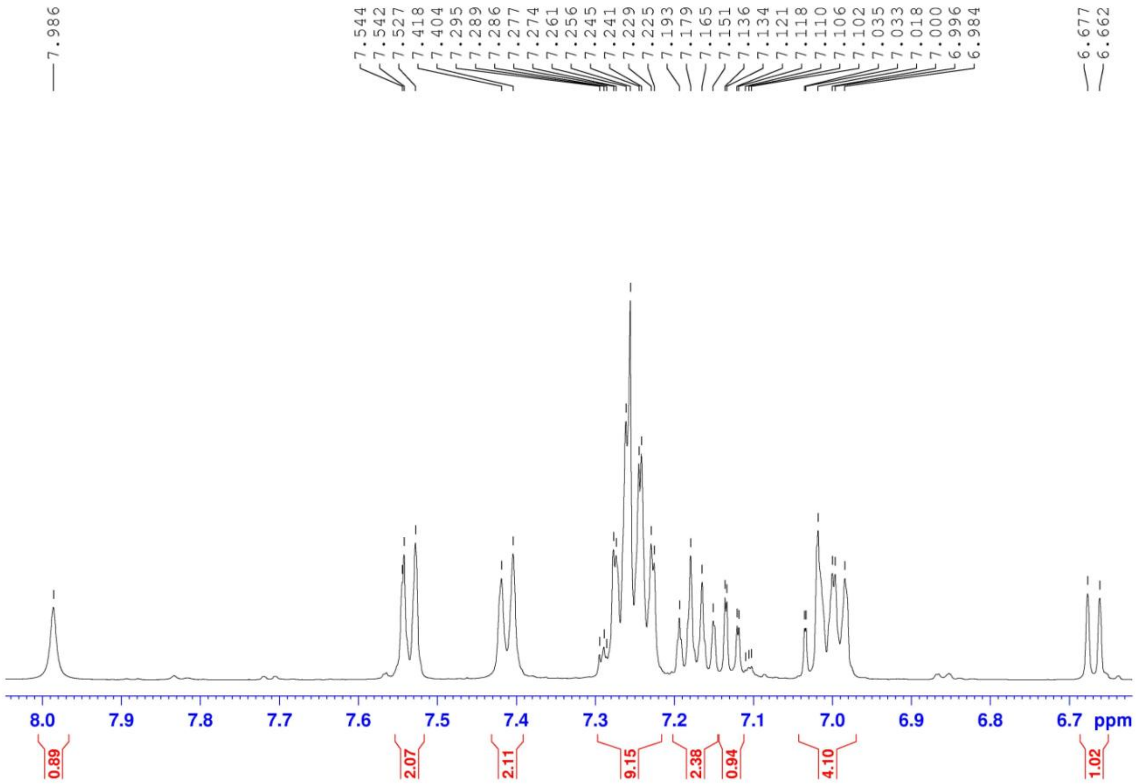

Figure S17. ^{13}C-NMR spectrum of **5a**.

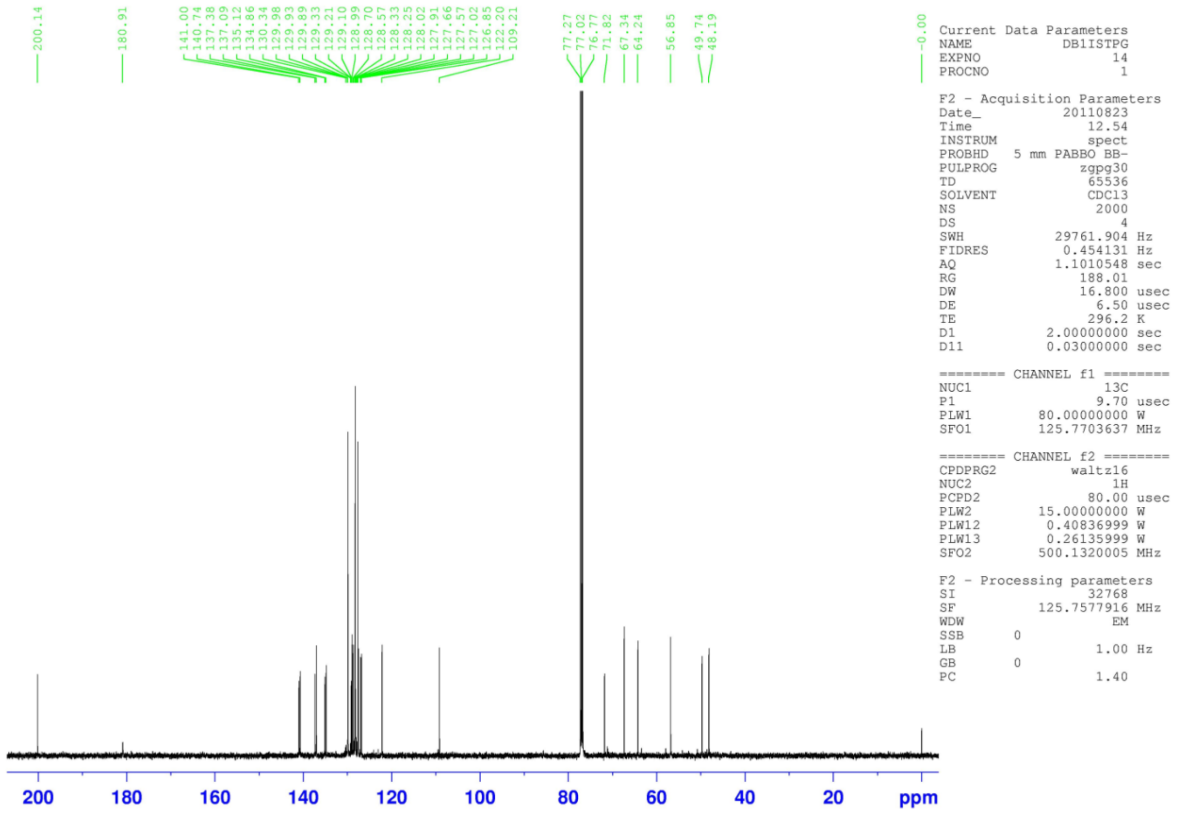

Figure S18. ^{13}C-NMR spectrum of **5a**.

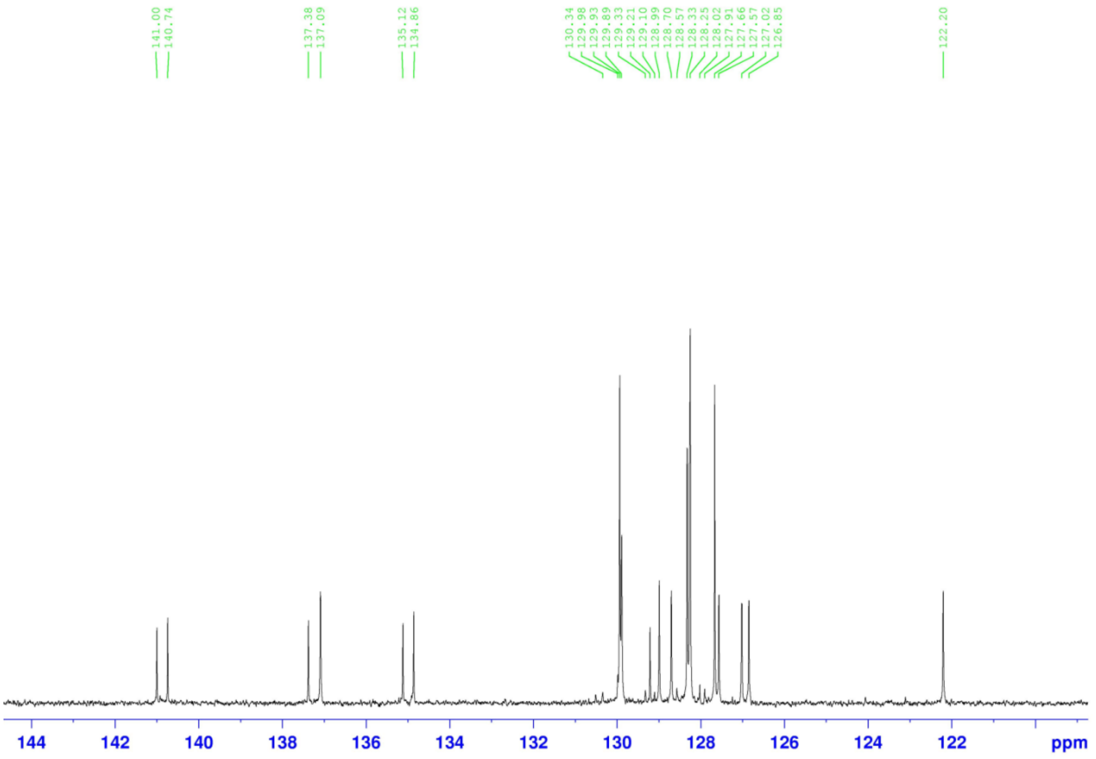

Figure S19. DEPT-135 NMR spectrum of **5a**.

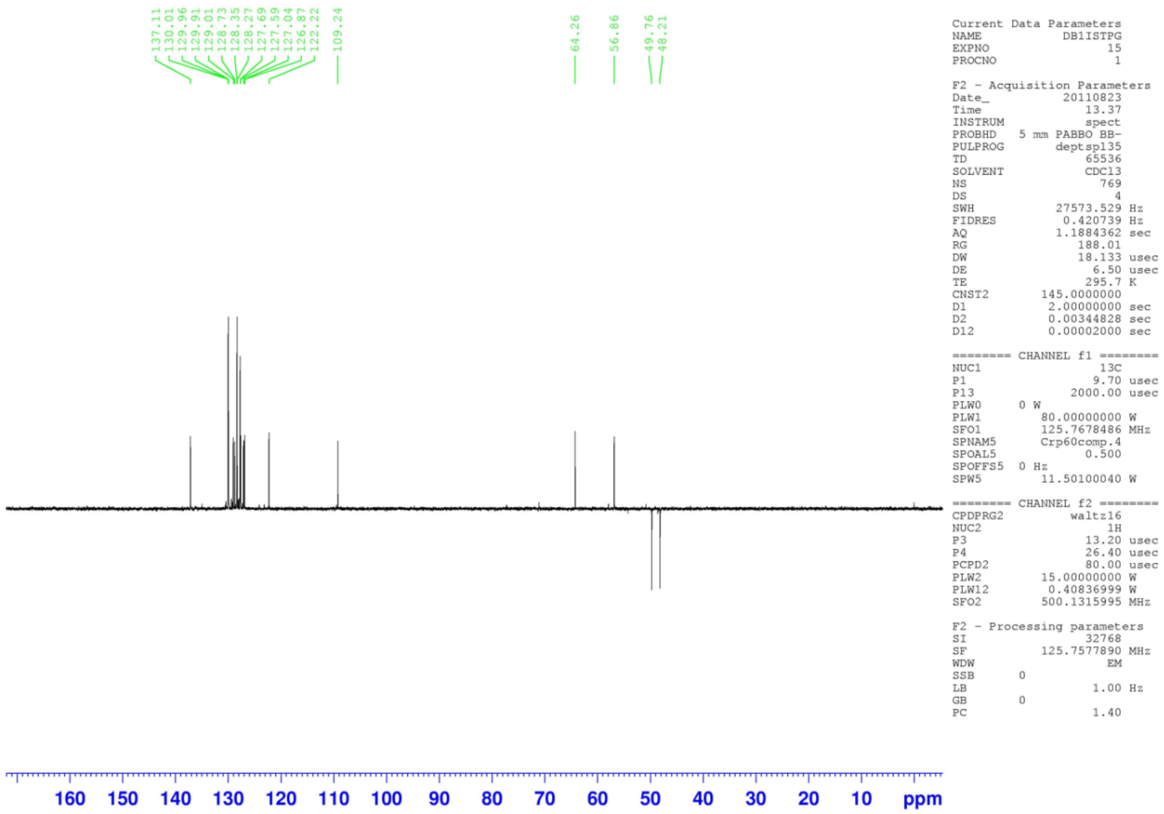

Figure S20. H,H-COSY spectrum of **5a**.

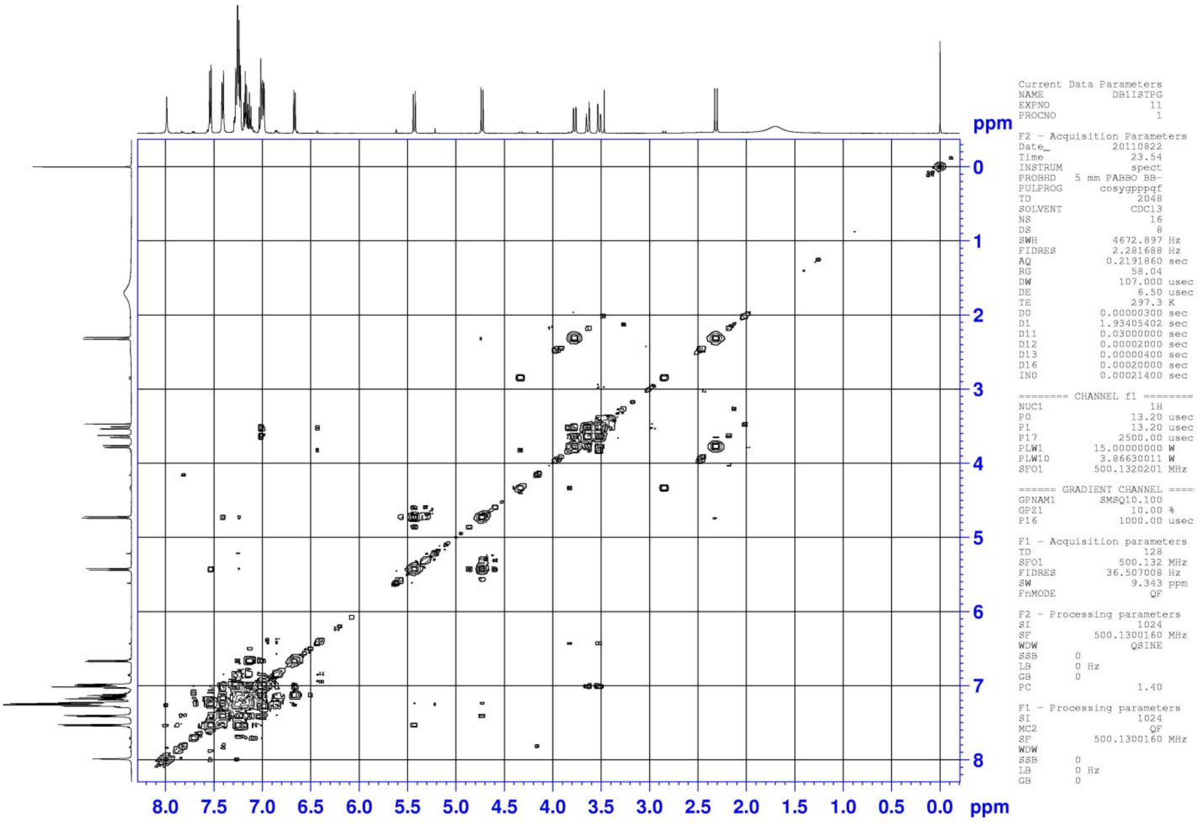

Figure S21. H,H-COSY spectrum of **5a**.

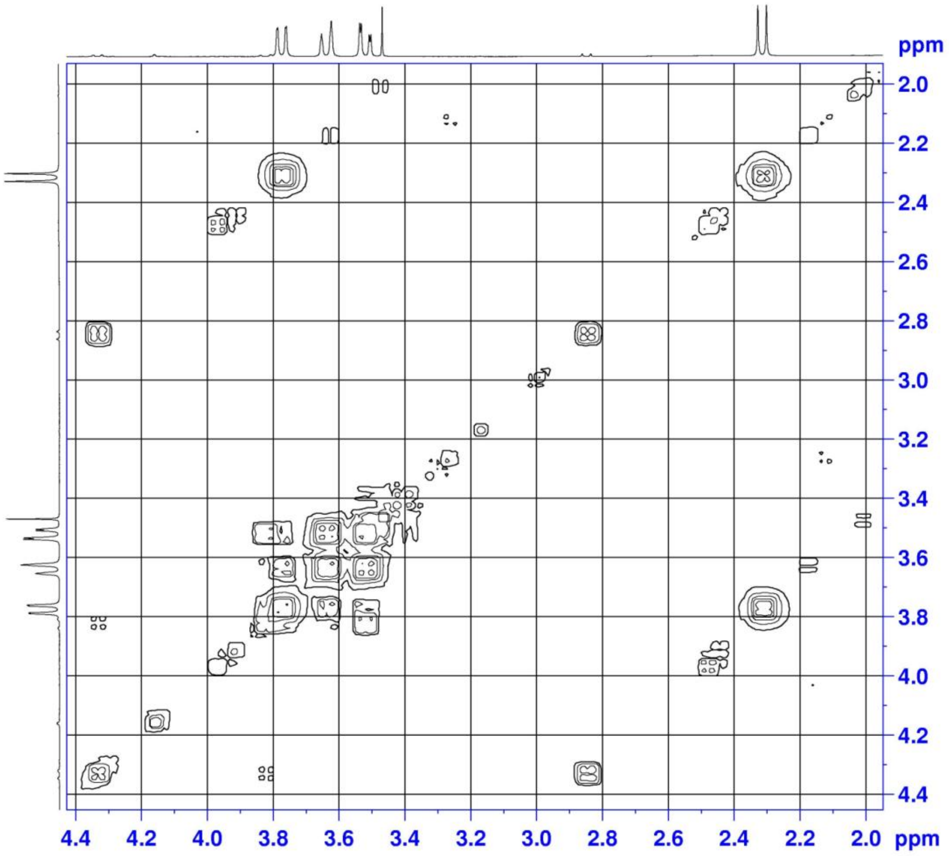

Figure S22. H,H-COSY spectrum of **5a**.

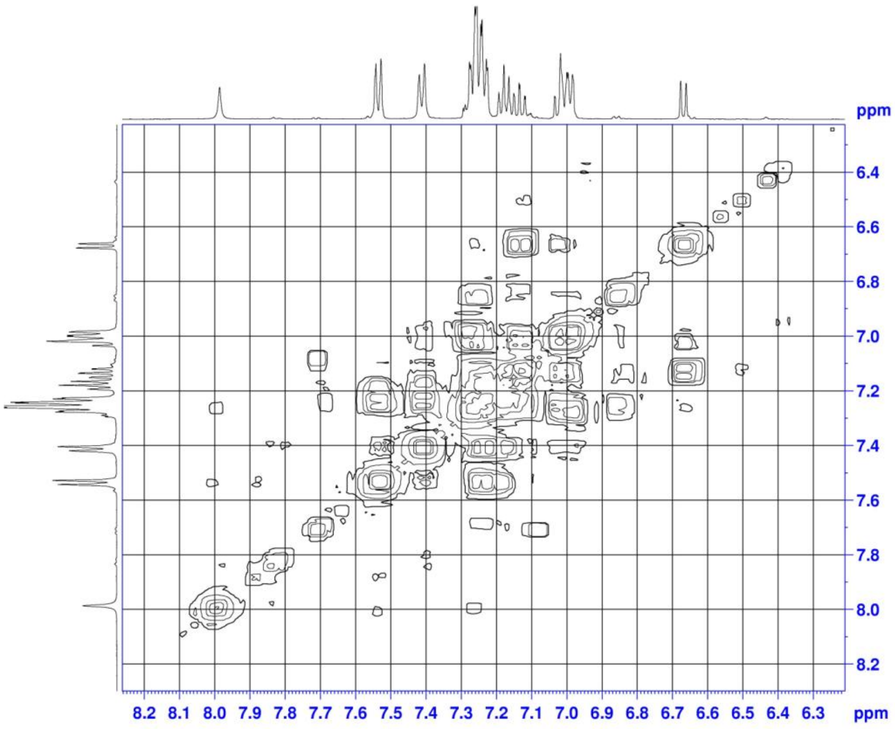

Figure S23. C,H-COSY spectrum of **5a**.

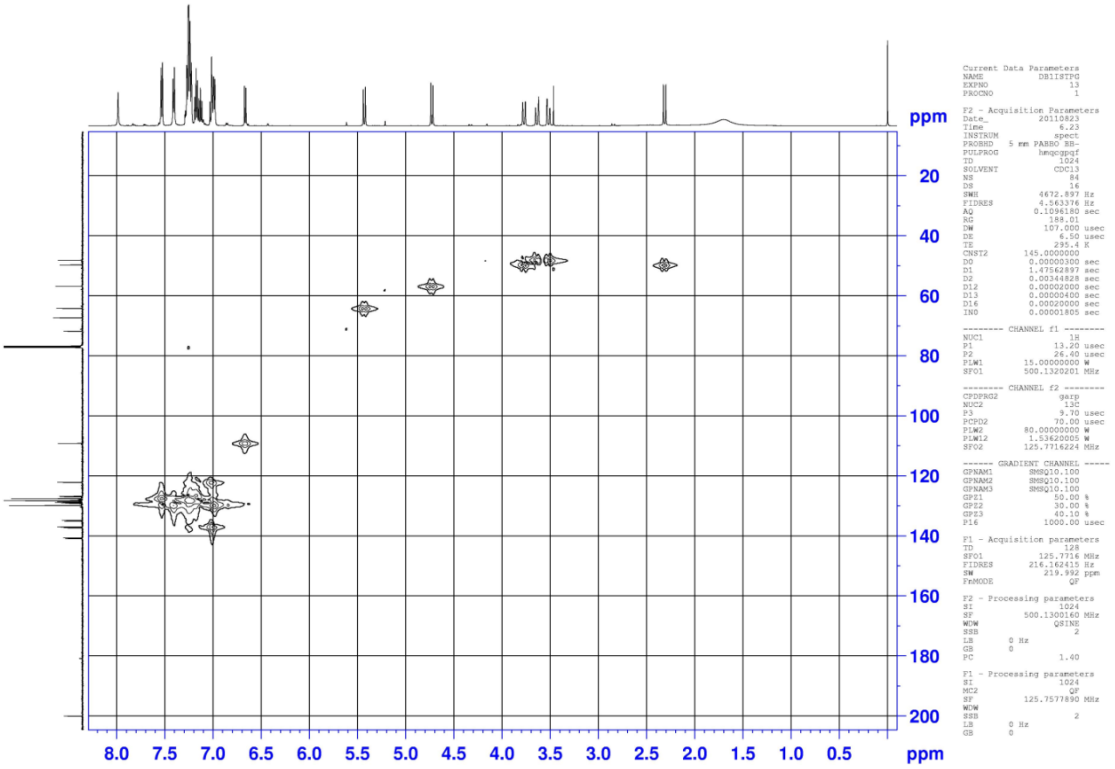

Figure S24. C,H-COSY spectrum of **5a**.

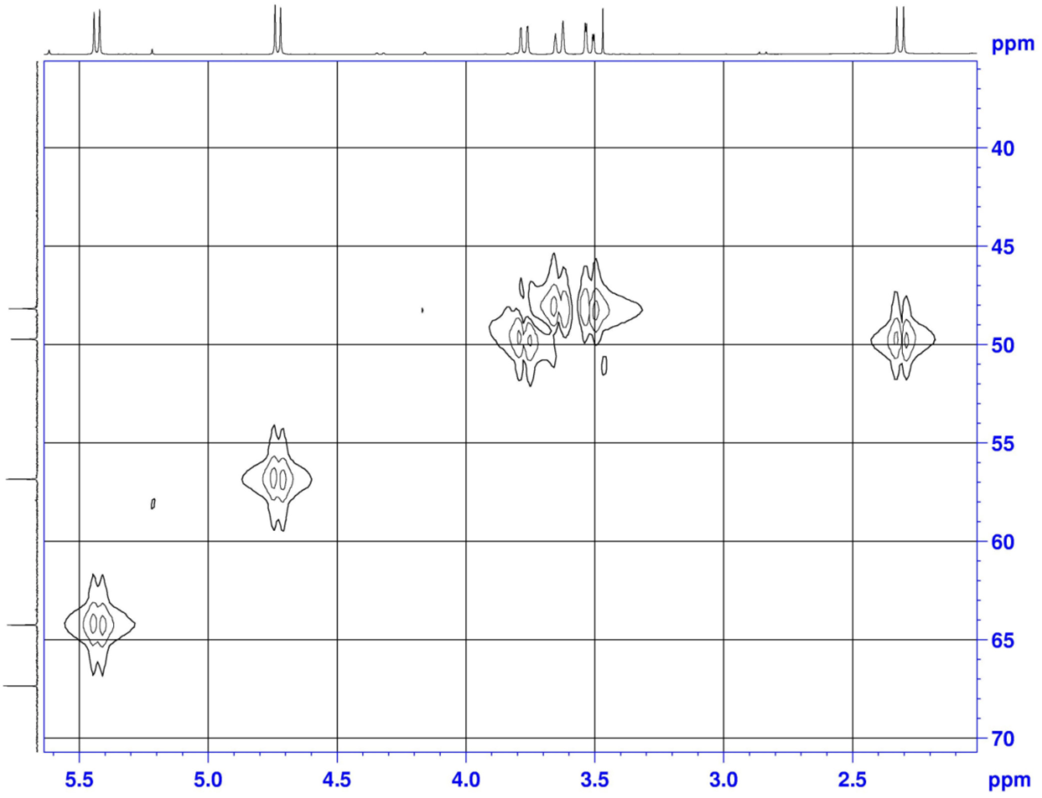

Figure S25. HMBC spectrum of **5a**.

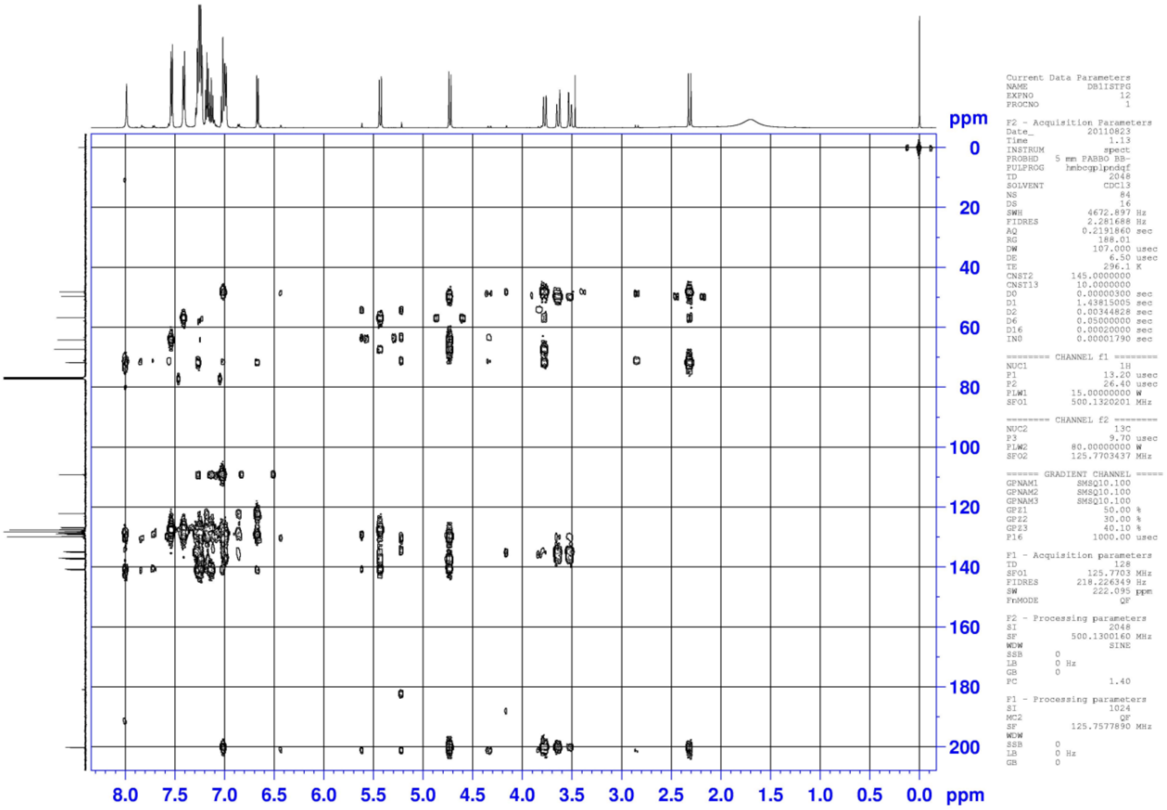

Figure S26. HMBC spectrum of **5a**.

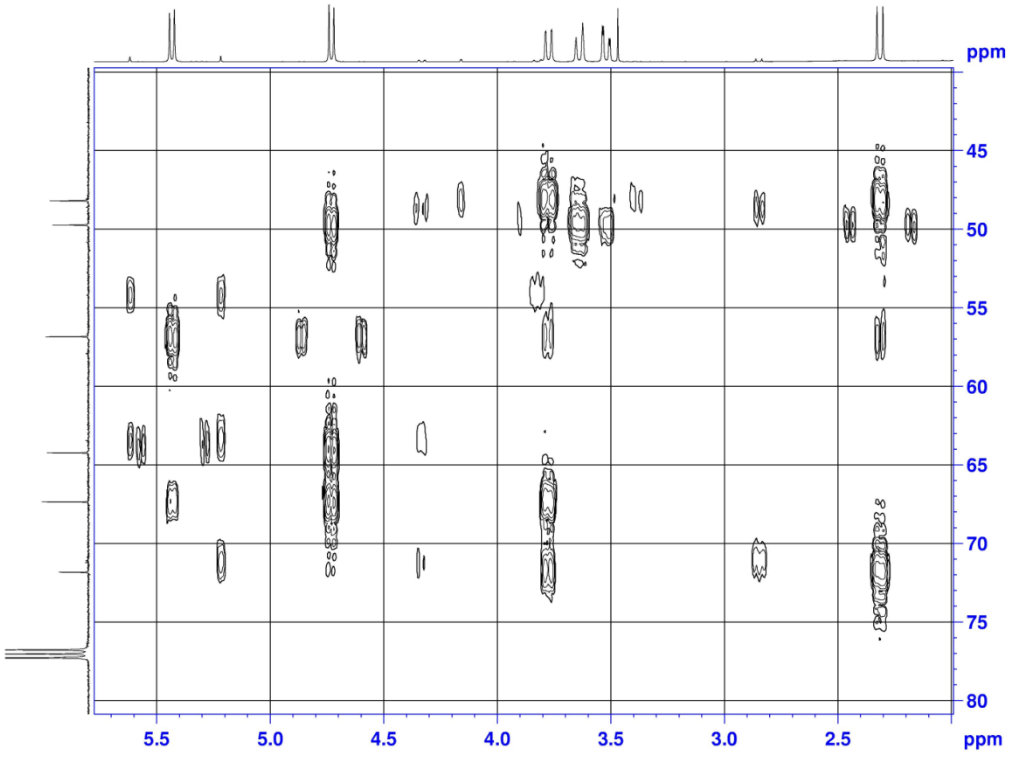

Figure S27. HMBC spectrum of **5a**.

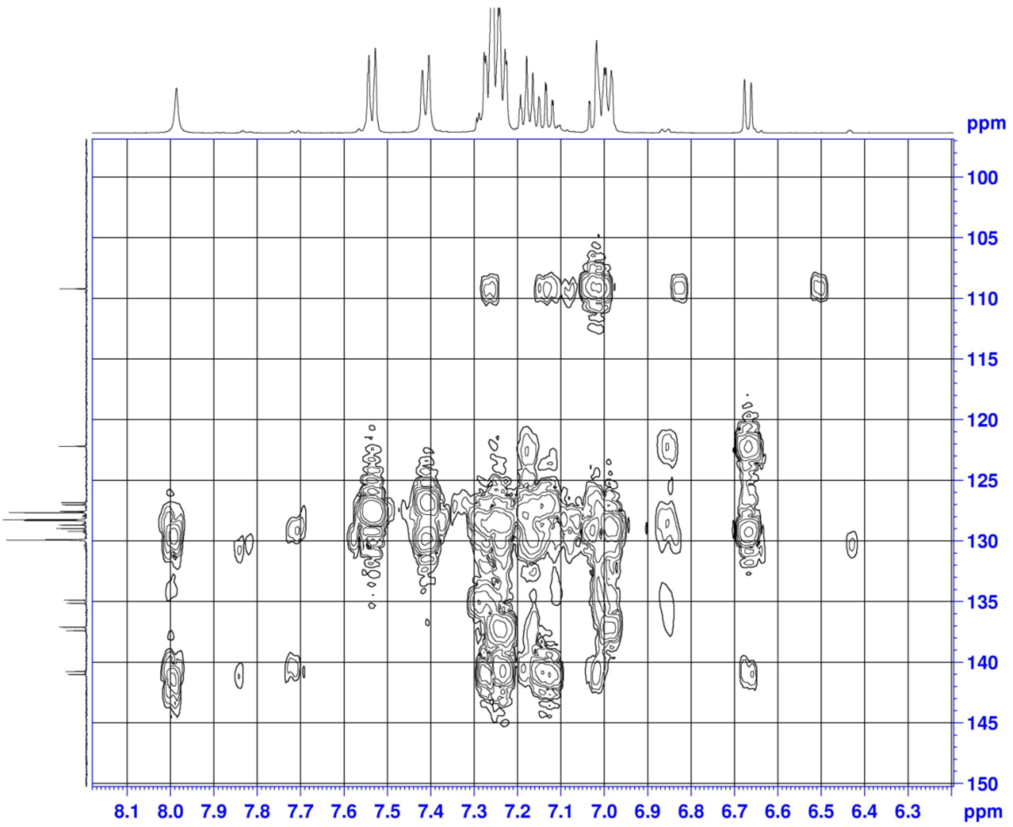

Figure S28. ^{1}H-NMR spectrum of **6f**.

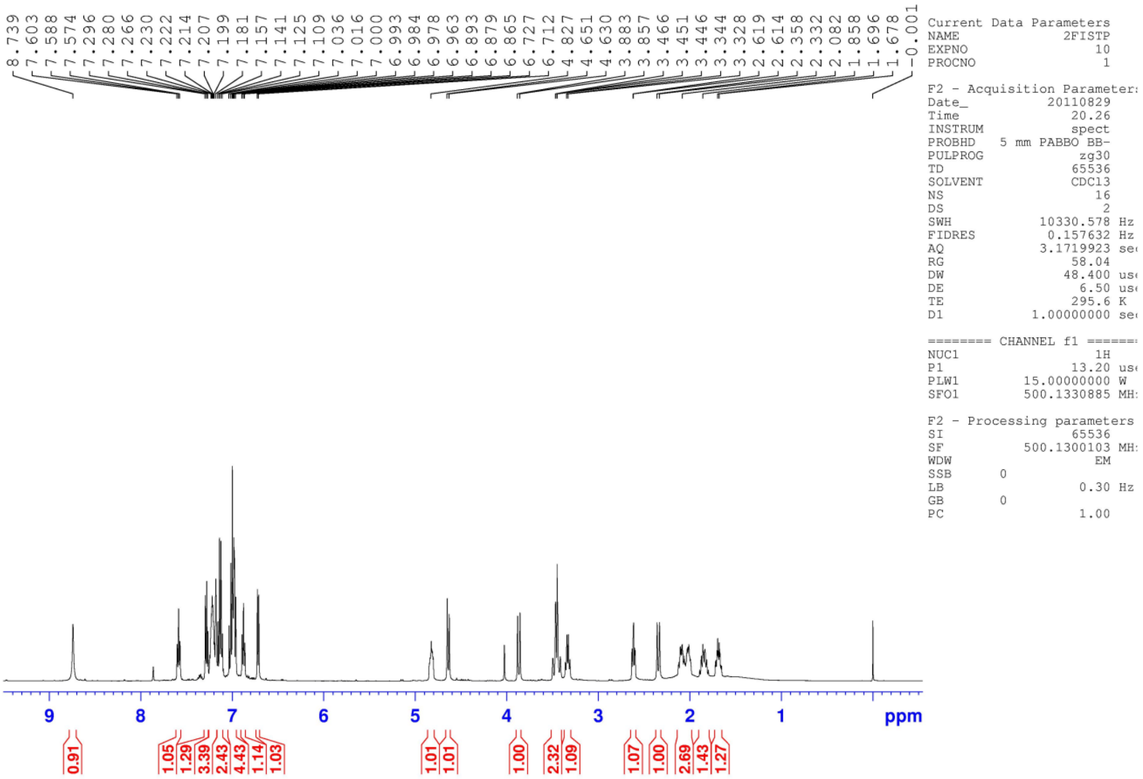

Figure S29. ^{1}H-NMR spectrum of **6f**.

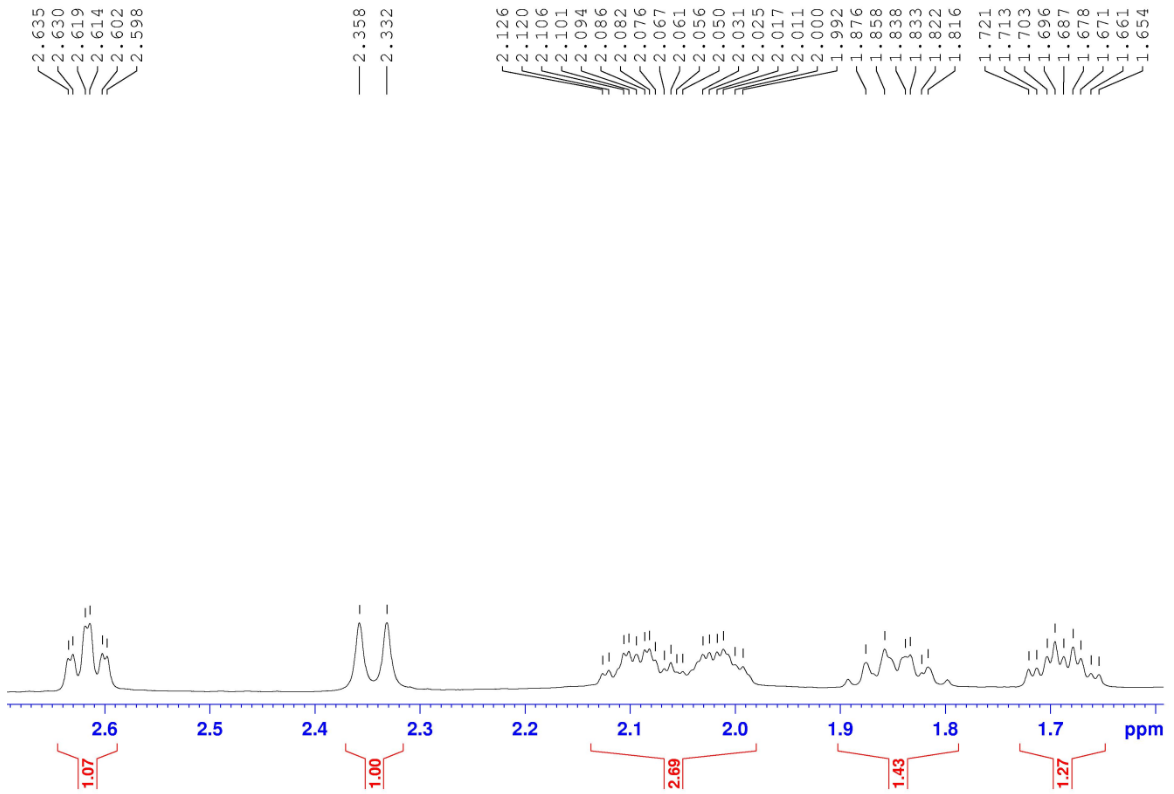

Figure S30. ^{1}H-NMR spectrum of **6f**.

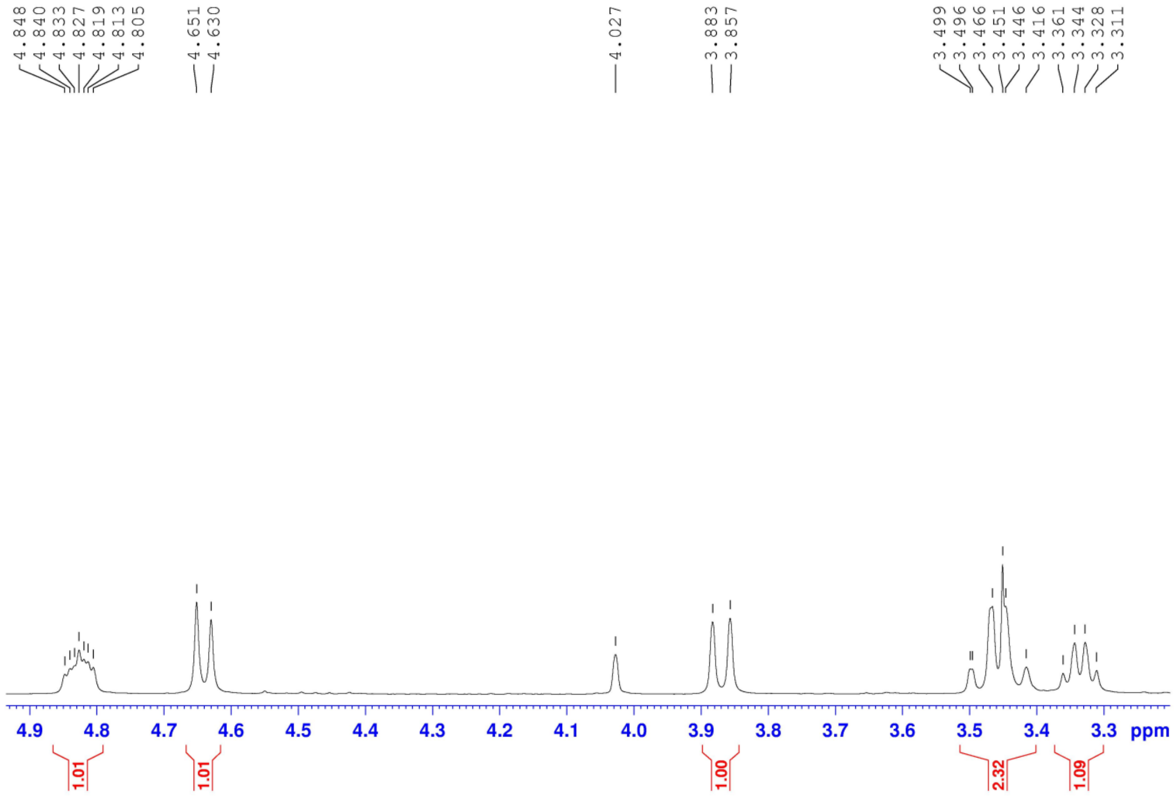

Figure S31. ^{13}C-NMR spectrum of **6f**.

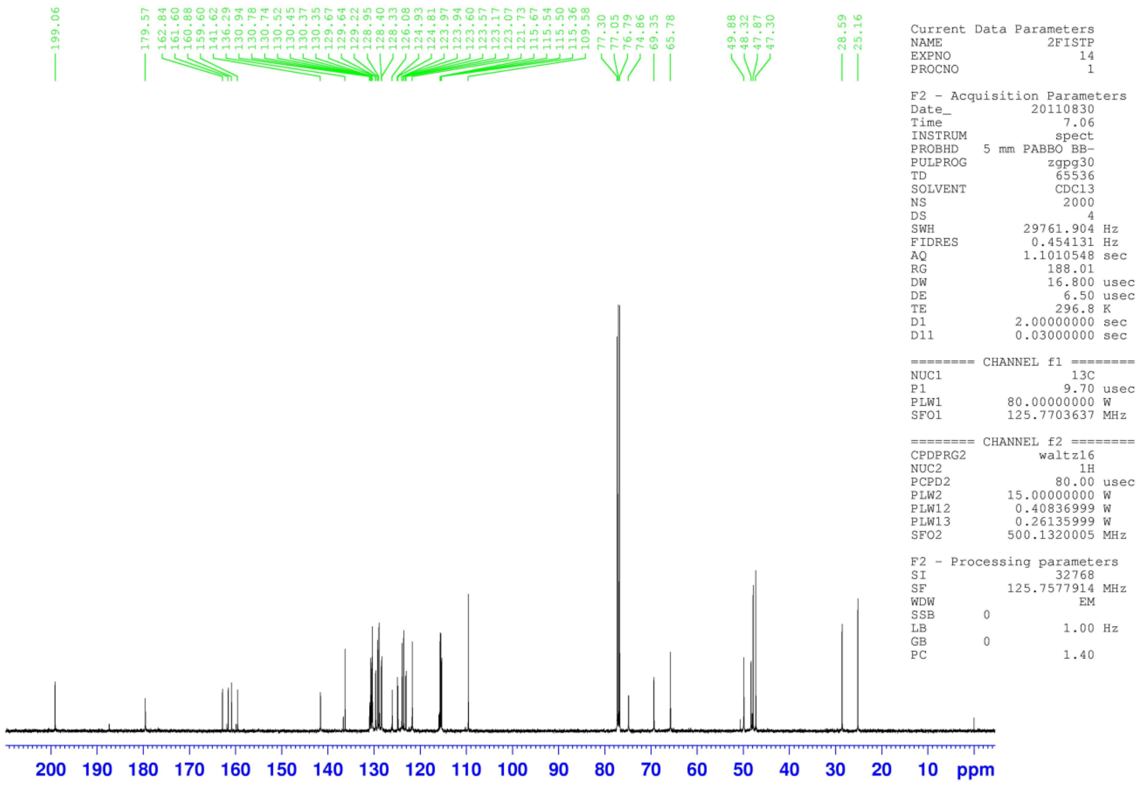

Figure S32. ^{13}C-NMR spectrum of **6f**.

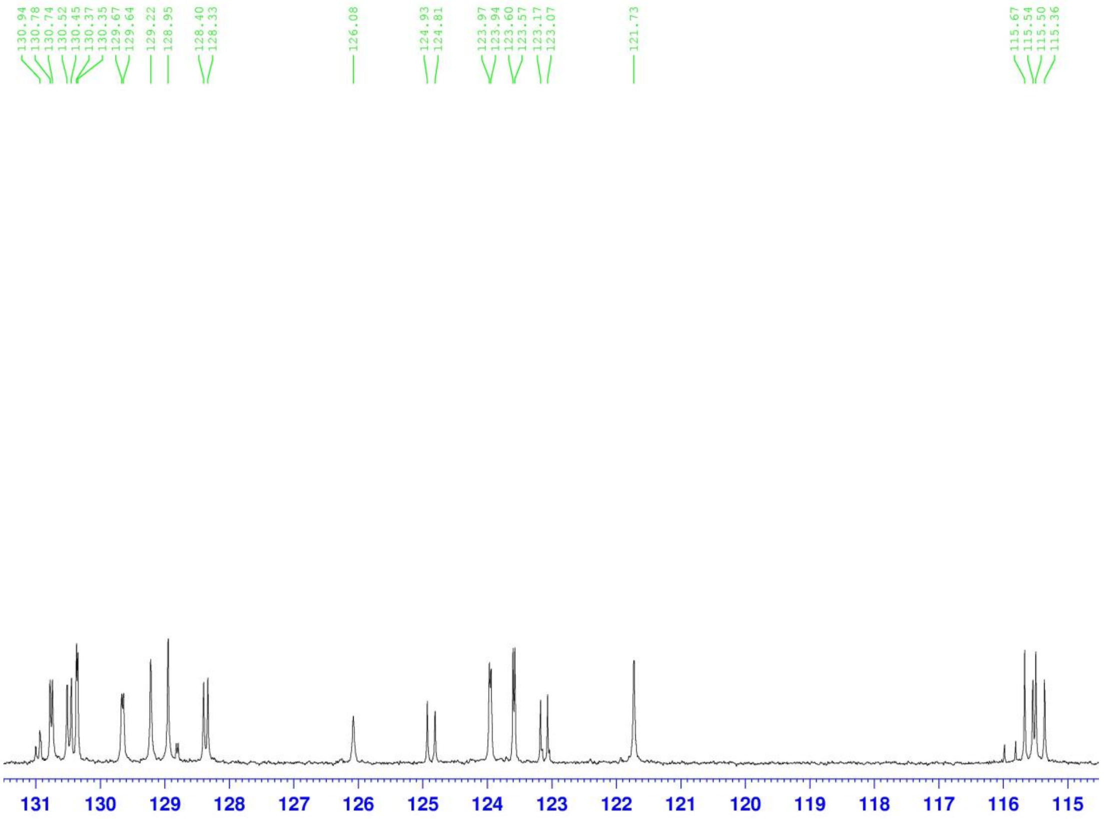

Figure S33. DEPT-135 NMR spectrum of **6f**.

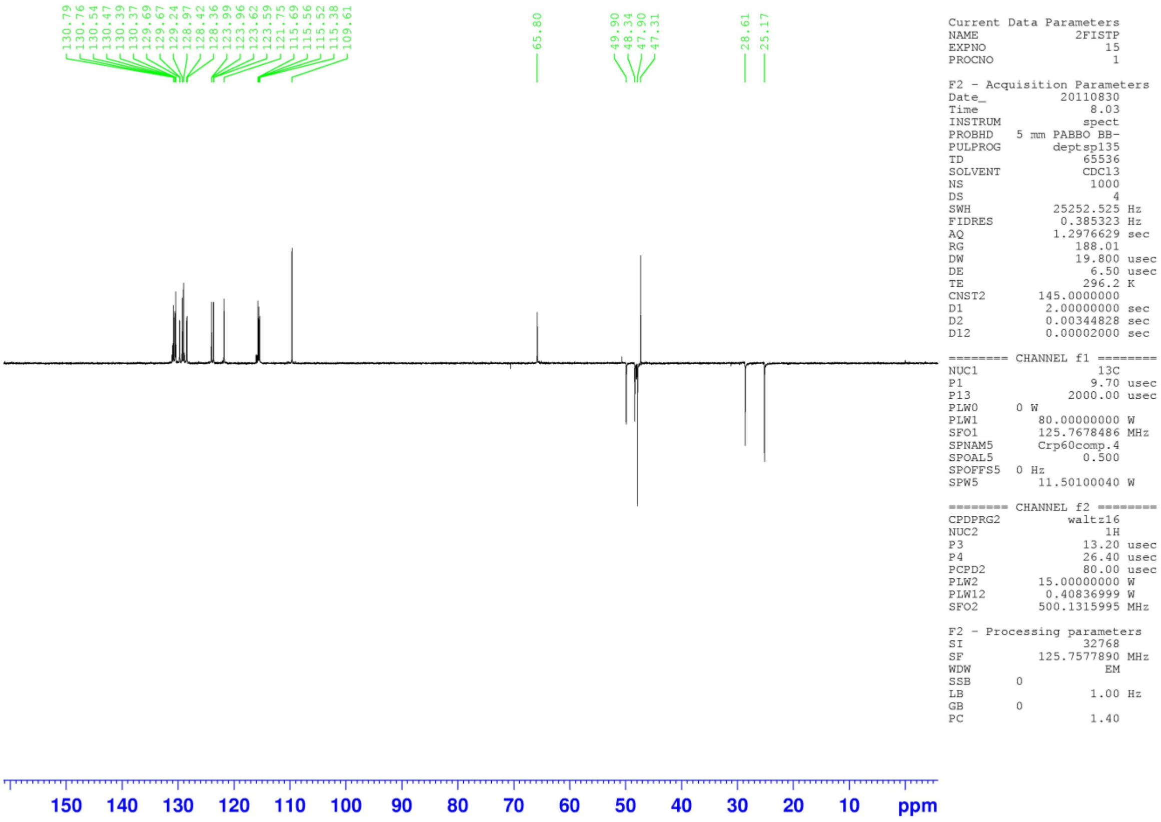

Figure S34. DEPT-135 NMR spectrum of **6f**.

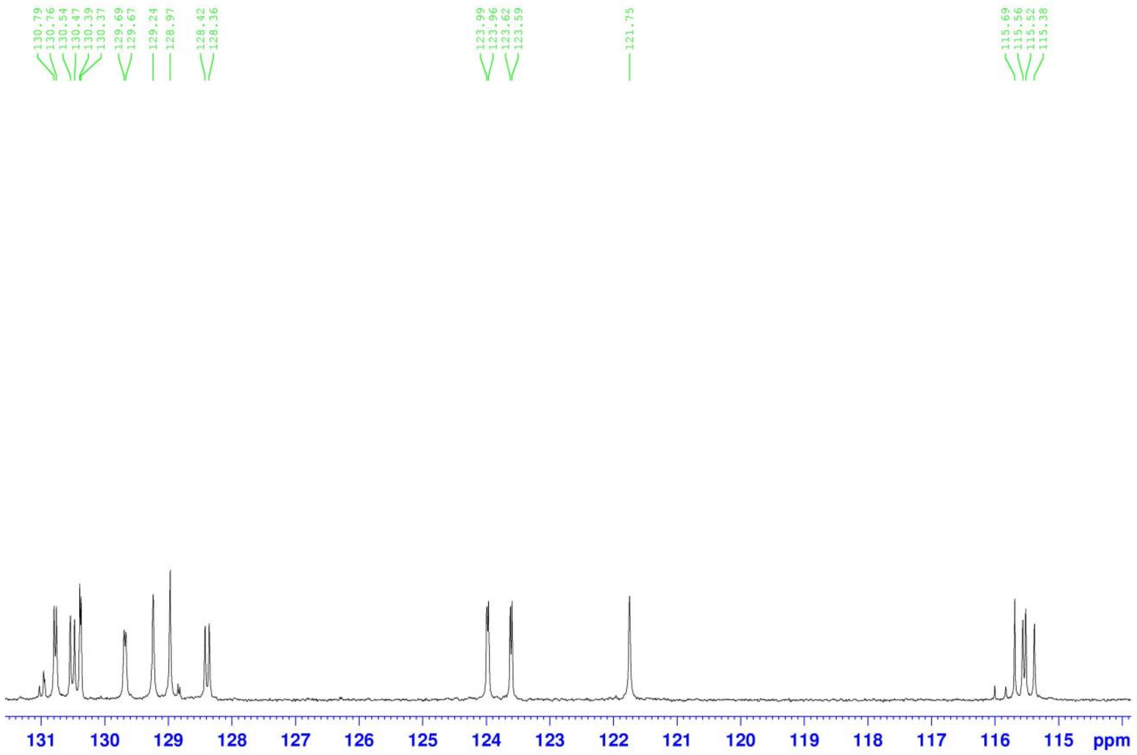

Figure S35. H,H-COSY spectrum of **6f**.

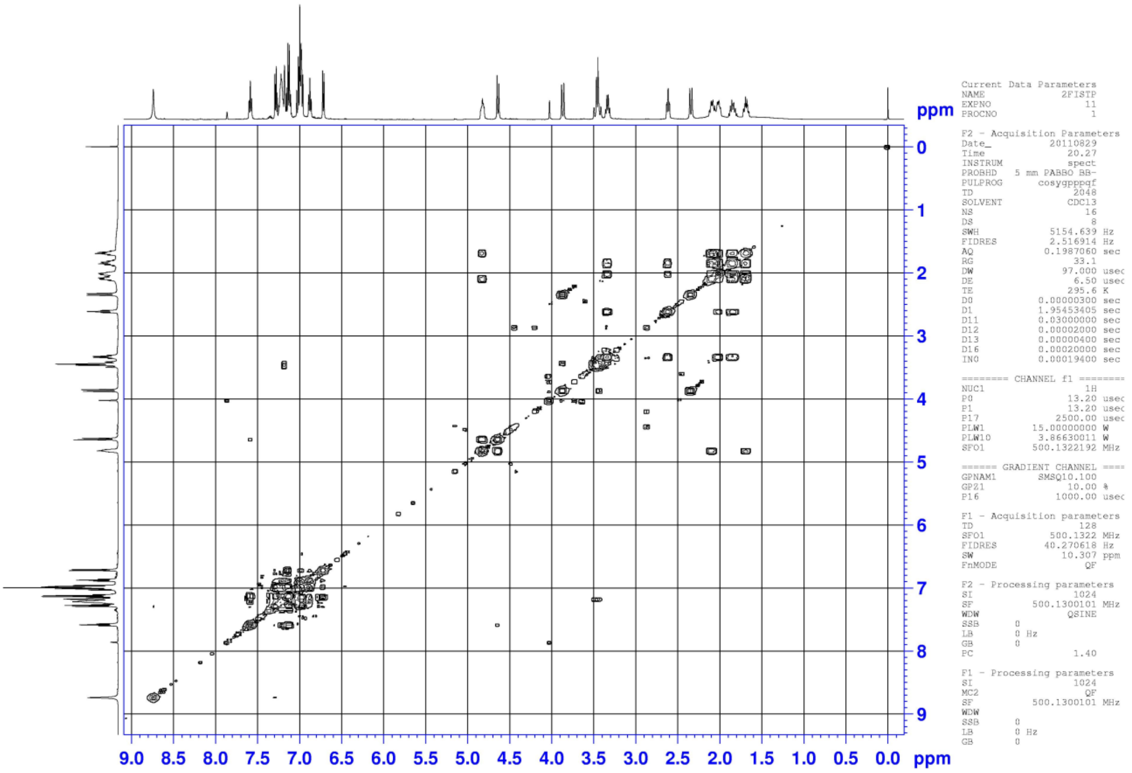

Figure S36. H,H-COSY spectrum of **6f**.

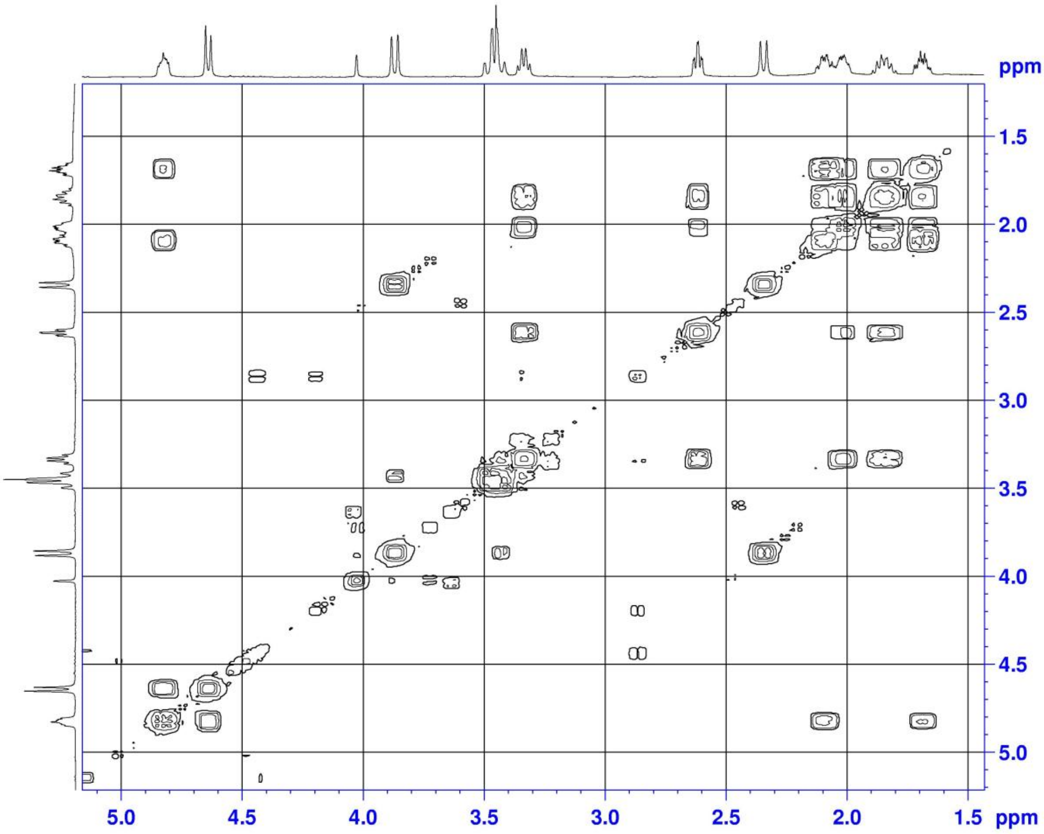

Figure S37. C,H-COSY spectrum of **6f**.

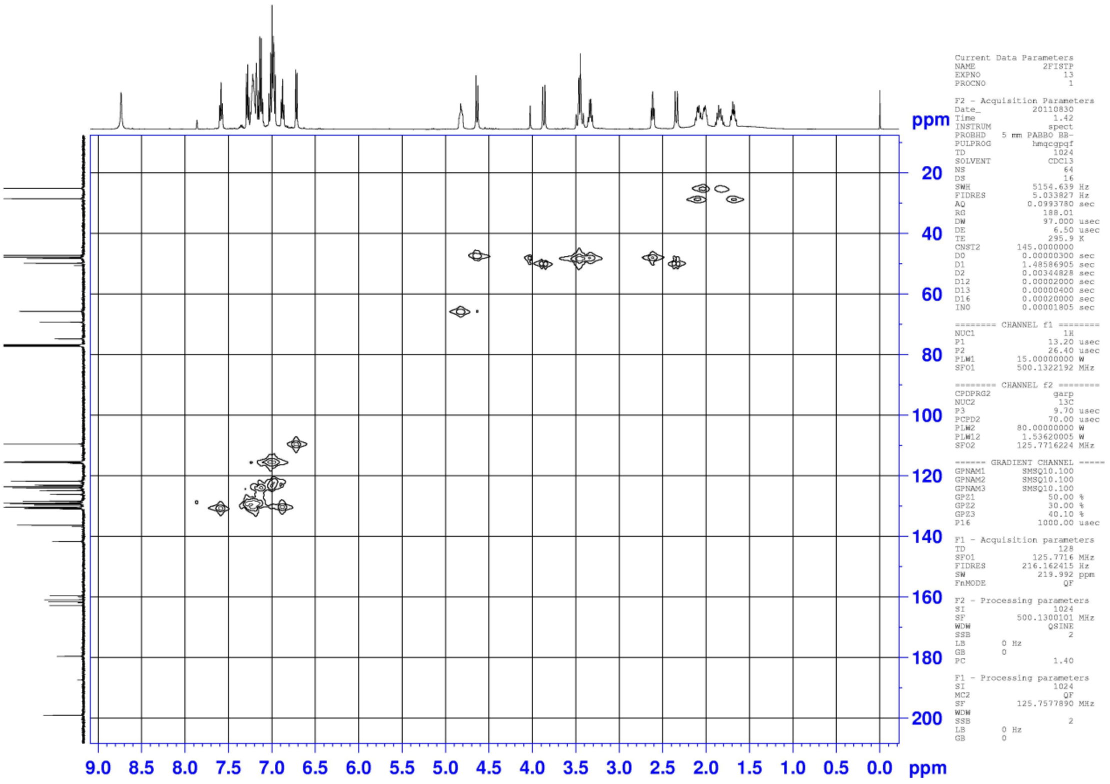

Figure S38. C,H-COSY spectrum of **6f**.

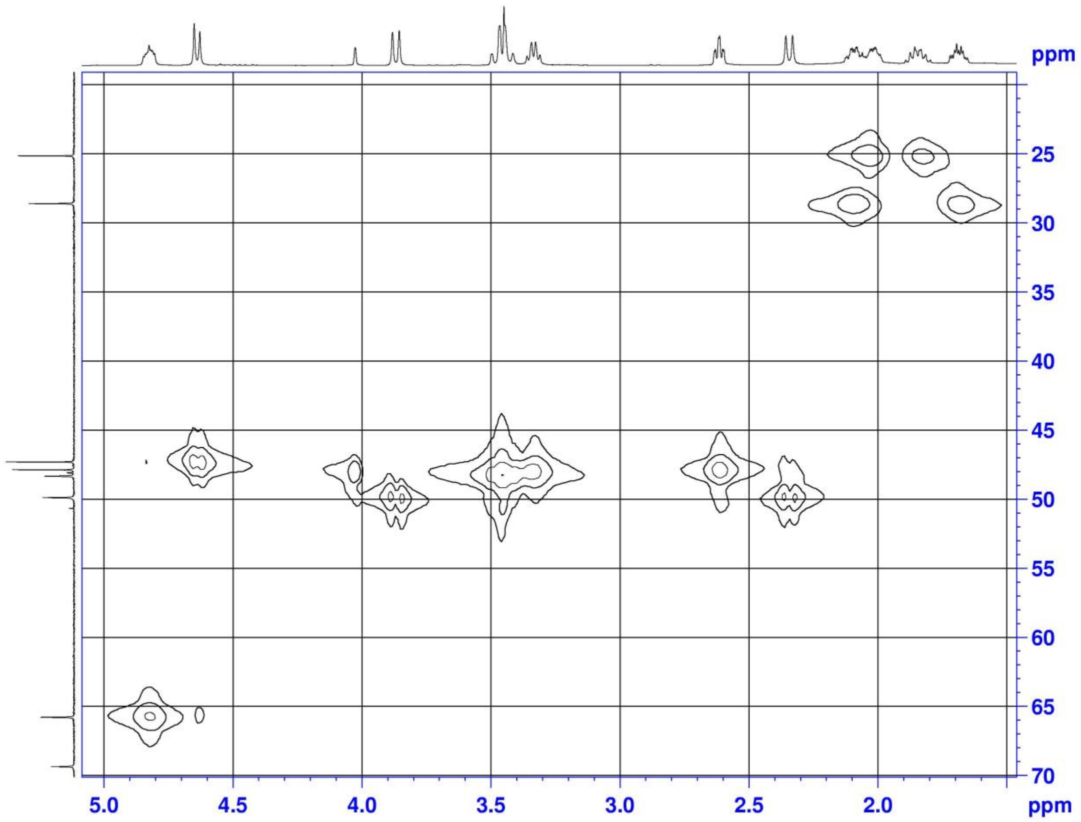

Figure S39. HMBC spectrum of **6f**.

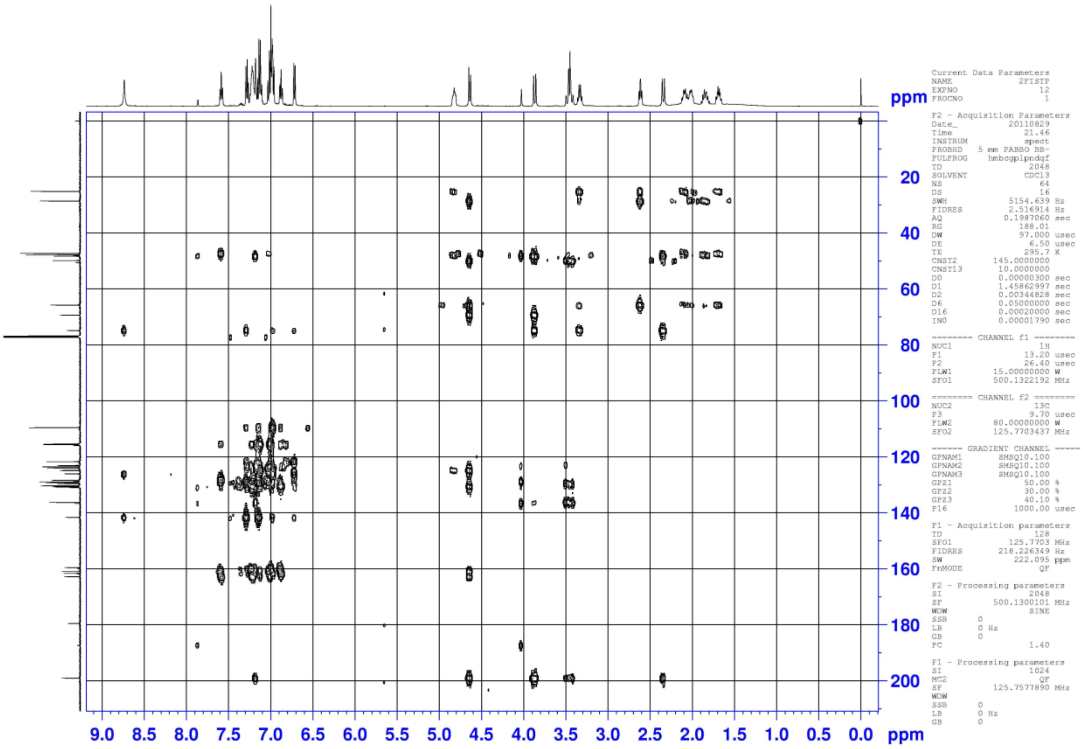

Figure S40. HMBC spectrum of **6f**.

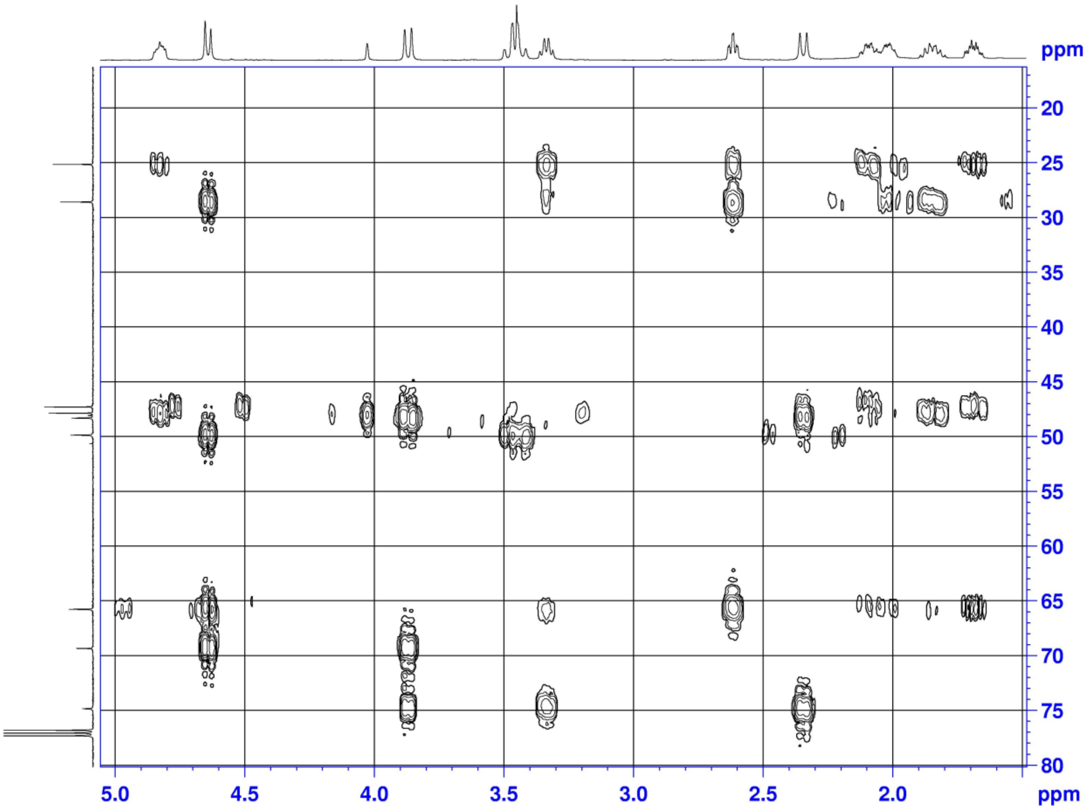

Table S1. Antiproliferative activity of **2a**–**n**, **4a**–**n**, **5a**–**n** and **6a**–**n** [1].

Ar	Comp	CCRF-CEM	MDA-MB-231	SK-OV-3	Comp	Yield (%)	CCRF-CEM	MDA-MB-231	SK-OV-3	Comp	Yield (%)	CCRF-CEM	MDA-MB-231	SK-OV-3	Comp	Yield (%)	CCRF-CEM	MDA-MB-231	SK-OV-3
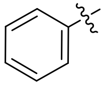	**2a**	51.68	85.51	73.60	**4a**	92	37.07	35.48	−21.46	**5a**	93	53.56	77.03	58.83	**6a**	90	61.01	87.01	72.54
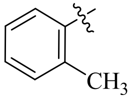	**2b**	51.81	87.72	73.91	**4b**	90	58.88	41.33	18.30	**5b**	90	52.22	83.16	72.02	**6b**	91	62.34	81.59	74.04
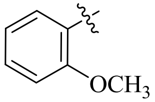	**2c**	56.74	88.72	73.11	**4c**	86	52.32	26.60	−10.61	**5c**	85	53.72	86.53	63.20	**6c**	83	60.92	85.47	71.11
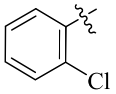	**2d**	51.61	87.87	−41.45	**4d**	91	51.14	0.00	−43.19	**5d**	92	45.03	74.88	51.68	**6d**	92	56.48	73.80	18.62
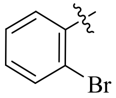	**2e**	62.06	86.45	56.17	**4e**	90	48.21	24.80	−24.73	**5e**	89	45.61	58.83	55.69	**6e**	90	57.85	72.67	23.91
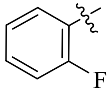	**2f**	53.34	85.94	−33.65	**4f**	92	49.50	45.67	−1.07	**5f**	90	46.89	81.67	40.56	**6f**	89	28.13	76.71	−13.31
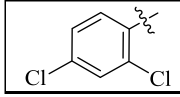	**2g**	47.40	87.41	−25.73	**4g**	90	40.99	53.30	3.69	**5g**	92	43.99	82.03	22.47	**6g**	91	29.56	60.52	−18.16

Table S1. *Cont.*

Ar	Comp	CCRF-CEM	MDA-MB-231	SK-OV-3	Comp	Yield (%)	CCRF-CEM	MDA-MB-231	SK-OV-3	Comp	Yield (%)	CCRF-CEM	MDA-MB-231	SK-OV-3	Comp	Yield (%)	CCRF-CEM	MDA-MB-231	SK-OV-3
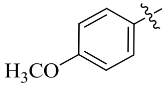	**2j**	53.75	75.69	50.53	**4j**	85	48.28	53.91	36.37	**5j**	86	64.15	84.90	61.03	**6j**	82	62.29	81.52	60.35
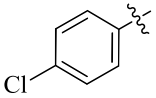	**2k**	49.31	76.84	56.05	**4k**	93	57.70	70.86	68.28	**5k**	90	56.57	84.07	67.80	**6k**	92	62.44	86.84	68.65
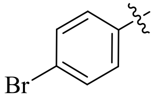	**2l**	47.33	74.93	43.78	**4l**	91	56.01	71.96	70.52	**5l**	92	57.45	81.95	70.28	**6l**	89	64.03	86.09	61.38
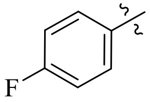	**2m**	48.44	74.63	50.25	**4m**	94	57.88	69.09	56.58	**5m**	93	57.91	84.75	73.89	**6m**	90	64.03	85.60	56.75
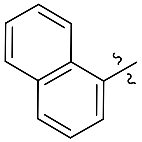	**2n**	46.70	84.03	12.23	**4n**	90	51.20	79.55	69.64	**5n**	87	57.35	73.62	71.35	**6n**	89	26.83	−0.57	−41.20

[1] The control was calculated to be 0% inhibition.
